# Mapping determinants of alternative protein food intake across 13 European countries: food system stakeholders’ perspectives

**DOI:** 10.1186/s12966-026-01891-3

**Published:** 2026-02-19

**Authors:** Hanna Zaleskiewicz, Ewa Kulis, Zofia Szczuka, Anna Banik, Gabriela Alcat, Concha Ávila, Isabel Cardoso, Feyza Başak Coşkun, Anna Kornafel, Lisa Mai, Lisa Kolden Midtbø, Jowita Misiakowska, Maja Oblak, Vesna Miličić, Pedro Queiroz, Giorgia Sabbatini, Britt Sandvad, Maria Siwa, Per Solibakke, Šárka Stejnarová, Katharina Stollewerk, Rosa Strube, Eleni Tsapa, Ariane Voyatzakis, Eirini Xaxiri, Aleksandra Luszczynska

**Affiliations:** 1 Institute of Psychology, SWPS University, 30B Ostrowskiego St, Wroclaw, 53-238 Poland; 2 Research Development and Innovation Department, FIAB - Spanish Food and Drink Federation, Calle Velázquez, 64. 3, Madrid, 28001 Spain; 3 Regulatory and Scientific Affairs Department, FIPA - Portuguese Food and Drink Federation, Rua da Junqueira, 39 – Edifício Rosa – 1º Piso, Lisbon, 1300- 307 Portugal; 4 SETBIR - Union of Dairy, Beef, Food Industrialists and Producers of Turkey, Mustafa Kemal Mah. 2126. Sok. No:4 Gökçen Prestij İş Merkezi, Kat:4 D:13, Çankaya, Ankara, Turkey; 5 CSCP - Collaborating Centre on Sustainable Consumption and Production, Hagenauer Strasse 30, Wuppertal, 42107 Germany; 6 Møreforsking AS, Ålesund, 6009 Norway; 7 CCIS-CAFE - Chamber of Commerce and Industry of Slovenia – Chamber of Agricultural and Food Enterprises, Dimičeva ulica 13, Ljubljana, 1000 Slovenia; 8 FEDERALIMENTARE – Federazione Italiana dell’Industria Alimentare, Viale Luigi Pasteur, 10, Rome, 00144 Italy; 9 Food & Bio Cluster Denmark, Agro Food Park 15, Aarhus N, 8200 Denmark; 10 CIAFRI - Czech Institute for Agri-Food Research and Innovation, Počernická 272/96, Prague, 108 03 Czech Republic; 11 LVA – Lebensmittelversuchsanstalt, Zaunergasse 1–3, Vienna, 1030 Austria; 12 SEVT – Federation of Hellenic Food Industries, Kifisias Av. 340, N. Psichiko, Athens, 154 51 Greece; 13 ANIA, French Food and Drink Federation, 9 Boulevard Malesherbes, Paris, 75008 France

**Keywords:** Alternative protein food, Stakeholder, System mapping

## Abstract

**Background:**

Using the system mapping approach, this study aimed to map the system of potential determinants of including alternative protein food (APF) into the daily diet across 13 European countries. We also aimed to identify key leverage points in the systems, which are determinants that are most interconnected with other determinants in the system. Similarities in leverage points and feedback loops found across the system maps were investigated.

**Methods:**

Food system stakeholders (*N* = 166; including food producers, food processors, policy makers, nutritionists, consumers, etc.) participated in 17 system mapping workshops, conducted in Austria, the Czech Republic, Denmark, France, Germany, Greece, Italy, Norway, Portugal, Slovenia, Spain, Turkey, and Poland. Group model building methods were applied to develop 17 maps. Centrality indices were calculated, feedback loops were identified, and similarities between countries were explored.

**Results:**

Key leverage points that were common across 6–8 maps included: consumer education and knowledge about APF, social norms, encouragement, advertising and influencers’ impact. Other common identified leverage points (present in 3–4 maps) were: health-related perceptions, curiosity/fear of novelty, national food culture, perceptions of ultra-processing, sustainability issues, and animal welfare issues. Overall, stakeholders emphasized consumers as central actors in the food system. Feedback loops identified in the study, revealed some common within-country pathways. For example, half of the feedback loops shown in the Italian maps included a potentially reinforcing upward spiral. Stakeholders perceived that higher APF product safety may facilitate advertising APF (e.g., as healthy), which in turn could increase consumers’ curiosity and/or beliefs that APF is healthy and sustainable. This was perceived as facilitating local development and job creation, food prices reduction, and further developments in food safety.

**Conclusions:**

The findings offer new insights into the complexities of European food systems and may contribute to the broader uptake of APF. Identifying leverage points and feedback loops may inspire food system stakeholders to design novel interventions. For example, these interventions could involve influencers to familiarize consumers with APF, promote knowledge about healthiness and safety of APF, as well as link APF consumption with positive emotions and social approval.

**Supplementary Information:**

The online version contains supplementary material available at 10.1186/s12966-026-01891-3.

## Background

Across European countries, animal products, such as eggs, dairy, and meat, are the most common, traditional protein choices [1]. However, nutritional choices based on these traditional proteins are not sustainable, as they are the main drivers of environmental degradation, ecosystem destruction, and climate change [1]. For example, compared to animal-based proteins, production of plant proteins is sustainable due to lower water consumption, lower fossil energy use, and lower waste generation, whereas microorganism-based protein production utilizes minimal resources and offers a method for reducing agricultural and industrial waste [2]. *Alternative protein food (APF)* products include protein concentrates obtained from processing plants (e.g., fava beans), as well as insects, krill, microbial biomass, and fungi [3]. Furthermore, the term “alternative” may refer to sources of proteins with a lower environmental impact compared to traditional protein sources [3]. Thus, APF may currently exclude cultured meat due to its high energy-demanding production [3, 4].

As evidence of sustainability-related benefits of APF continues to accumulate, research should also aim to identify the determinants of APF choice. These determinants should be perceived by stakeholders as important “leverage points”—that is, determinants which, if modified, may trigger substantial changes across the system of determinants, because they are highly interconnected with other operating factors within the complex food system. Since it remains unknown how food system stakeholders perceive the system of APF determinants, this study aims to explore this issue. Understanding stakeholders’ perspectives is crucial, as it allows the content of APF-promoting interventions to be aligned with stakeholders' views, thereby securing their approval and support for the implementation of such actions.

Changes in consumers’ nutrition behaviors, such as selecting more sustainable types of proteins, may shape the actions of other food system stakeholders and, thus, open pathways toward sustainable food systems [1, 5, 6]. Although previous research on stakeholders transforming European food systems into more sustainable ones indicates that food producers are considered the most important stakeholders [7], recent studies observe a power shift toward consumer influence on the food system. Consequently, there is an increased interest in research on determinants of consumer actions [7]. Furthermore, according to the *complex system approach models applied to food systems* (cf. [8–12]), a change initiated by one type of system actor (e.g., consumers) may cause changes reverberating throughout the system, mobilizing responses from other actors. One open question is how various food system stakeholders (e.g., food producers, food processors, nutrition specialists, etc.) perceive the key determinants that potentially enable consumers to choose alternative protein food.

According to socio-ecological models of human behavior [13–15], determinants of nutrition behaviors can be combined into broader categories of: (i) individual-level characteristics (e.g., beliefs, emotions); (ii) meso-level factors encompassing characteristics of family, local community, and workplace; and (iii) macro-level factors referring to characteristics of broader environment (e.g., national policies). Research on sustainable food choices, including APF, provides tentative support for socio-ecological models. A systematic review by Hoek et al. [16] on sustainable nutrition choices indicates that important types of determinants include individual-level factors such as “beliefs” and “attitudes,” as well as socioeconomic characteristics, taste, convenience, and familiarity. Environmental determinants include the availability of APF in local shops, social norms shared by family and friends, and family preferences, geographical characteristics (such as urbanization) and information in the environment (including advertising) [16]. Finally, macro-environmental factors may include national dietary guidelines and public policies [16]. Other systematic reviews have used the socio-ecological model to provide lists of specific individual-level determinants of APF intake such as neophobia, perceived environmental benefits or perceived health risks [17–19]. Unfortunately, it remains unclear which of these determinants of APF choices may be the most relevant.

Socio-ecological models and the research that uses them to map the determinants of APF choices lack specific predictions about how these determinants may be interconnected. However, closer analysis of these models suggests that they are aligned with a complex system perspective, assuming multi-level interdependent determinants. In particular, *complex systems thinking* considers interdependent relationships between determinants of behavior and views a problem as a dynamic, interdependent, and ongoing process [20, 21]. A change in one element is assumed to affect other parts of the system. Furthermore, these connections form feedback loops, which are non-linear associations that can lead to the growth or decline of a respective action (i.e., reinforcing loops), or they can have a stabilizing effect and may result in the system maintaining its status quo [20, 21]. System thinking is used to identify a set of determinants and their connections in the context of a specific target behavior. In other words, a set of determinants and their connections are specific to the target behavior such as the intake of APF [22].

Applying a complex system lens, this study aims to better understand how different factors within the food system are connected and how they influence people’s decisions to include alternative protein food in their daily diets. In particular, advancement of prior research on determinants of APF consumption [16–19] may be achieved through the use of the *system mapping approach*, which represents an innovative participatory action research process to advance theory development and sets the targets for public health interventions [23]. To date, system mapping has been used to explain the determinants of food sector development and food acceptability or availability in the context of energy-dense and ultra-processed foods, cocoa, and traditional proteins [24, 25]. However, maps of the system of determinants of sustainable food uptake, such as APF, are lacking.

A system mapping approach allows to connect stakeholder-identified determinants by showing how they intersect within a food system, while accounting for their interdependence. System mapping uses a participatory approach, which involves a group of stakeholders exploring how a system works using a structured, step-by-step process to create a map of proposed causal-loop diagrams of a complex system. Subsequently, these system maps help to inform responses to an investigated issue [26].

System maps allow for the identification of so-called leverage points - determinants that are most likely to trigger a change in the whole system [27, 28]. When altered, these leverage points have ripple effects throughout the system and can therefore evoke substantial changes [27]. The leverage points may be the main target of interventions, promoting higher uptake of APF by consumers.

### Study aims

The study aimed at mapping the system of determinants of an individual’s nutrition behavior, including the incorporation of APF into daily diets, and exploring the associations between these determinants. First, we focused on discovering the types of determinants that constitute common leverage points – identified across more than one country. Specifically, we aimed to identify determinants from the perspective of the major food system stakeholders, including food producers, food processors, retailers, health and education professionals, policymakers, and consumers. Second, the study investigated the interconnections between these determinants. Specifically, we aimed to find the common sequences of determinants that occur in the feedback loops proposed by the stakeholders.

We mapped the determinants and their relationships across 13 countries representing all major European regions: Austria, the Czech Republic, Denmark, France, Germany, Greece, Italy, Norway, Poland, Portugal, Slovenia, Spain, and Turkey.

## Methods

### Materials and general procedures

The *community-based system dynamics* (CBSD) [20] approach was applied as the overarching model, guiding the study design and procedures. This approach actively engages stakeholders in addressing the challenge at hand, positioning them as “experts” on how the system works before identifying potential actions that could be taken to improve the food system [20]. One of the structured formats used in CBSD is Group Model Building [29]. Group Model Building employs system mapping to create *causal loop diagrams* (CLDs), which provide a graphic representation of the complexity of a problem’s drivers (or determinants), from the food system stakeholders’ perspective [29, 30]. GMB guides stakeholders to collectively map their perceived drivers of a complex issue [20, 26] and encompasses a group’s collective ideas in a CLD (a system map). When developed using the CBSD model, these system maps are part of an iterative consensus process of examining a dynamic hypothesis to identify and revise postulated causal links [29, 30].

### Group model building workshops

Seventeen workshops were conducted to develop 17 system maps, with one workshop held in each of the following 12 countries: Austria, the Czech Republic, Denmark, France, Germany, Greece, Italy, Norway, Portugal, Slovenia, Spain, and Turkey. Five workshops were held in Poland. The study was approved by the Ethics Committee at SWPS University in Wroclaw, Poland (approval no. 01/E/03/2023). Additional approval was obtained in Norway in accordance with the MOREFORSKING institutional guidelines (approval no. 301103, SIKT).

### Workshop material development and facilitator training

The research team (ZS, EK, AL) developed a step-by-step workshop manual for use by the workshop facilitators (see Additional File 1). The manual explained the roles of facilitators, provided general rules of engagement, followed the principles of participatory research, specified the overall research questions, clarified the order and the timeline of the workshop preparations, and provided step-by-step instructions on how to conduct the workshop. Two facilitators – representing local non-governmental organizations – were selected by the local collaborating teams to lead the workshops in each of 13 countries. The facilitators participated in a one-day training session and received a recording of the workshop.

### Participant recruitment and pre-workshop information

The local teams recruited stakeholders of the food system, representatives of food producers and processors, policymakers, consumers, healthcare and education professionals working in the context of healthy diet promotion, representatives of NGOs operating in the food system, etc. At least two types of key food system stakeholders were recruited in each country. Another guiding rule was to ensure a balanced participation to avoid the dominance of one type of stakeholder over another type (e.g., avoiding one policymaker facing ten representatives of food producers).

The invitation was sent via email or delivered in person. It included standardized information about the LIKE-A-PRO project (https://cordis.europa.eu/project/id/101083961), information about the workshop content, procedures, dates, times, and location, as well as informed consent forms.

Two weeks prior to the workshop, participants who agreed to take part received a set of preparatory materials (see Additional File 1) and were asked to reflect critically on the information provided. The preparatory materials included: (i) definitions and examples of APF; (ii) definitions of system mapping and food systems; (iii) a framework used to categorize potential determinants of APF consumption; and (iv) an evidence-based list of examples of factors influencing consumers’ APF choices. Participants were instructed to review the content critically and consider which determinants may be the most important based on their own knowledge and professional experience. Similar pre-workshop strategies—designed to ensure a shared vocabulary and consistent access to evidence-based information among participants—have been used in previous system mapping studies [22, 29].

### Workshop conduct

The workshops took place from October to December 2023. Across 13 countries, the same workshop procedures (see Additional File 2) were followed. Socio-demographic data were collected in a post-workshop anonymous online survey. The post-workshop survey also included additional questions, addressing other aims of the study. Four workshops were organized in-person, one used a mixed delivery format combining online participation and in-person attendance, and the remaining 12 workshops were conducted using online teleconferencing.

The standardized workshop procedures involved four stages: (i) identifying the key determinants and defining them, as well as reaching consensus regarding the list of included determinants of APF intake; (ii) identifying the causal links between the determinants (A facilitates B) and reaching the consensus regarding the causal links; (iii) transforming the list of determinants and connections into an initial version of a system map, representing the consensus views of the group resulting from the discussion; (iv) reviewing the system map and refining the connections (e.g., adding any missing connections) by the stakeholders; and (v) a group consensus process and approval of the final system map. As the organizing principle, the intake of APF was not included in the system map, assuming that all determinants are connected to this variable [22, 29]. The support team was organized to ensure that any technical or organizational problems were resolved. For details see the workshop manual (Additional File 1).

STICKE 3.0 software (System Thinking in Community Knowledge Exchange; [31]), developed to facilitate workshop-based visualizations of system maps and conduct network analysis, was applied to draw the maps. This software was applied in previous stakeholder research in the context of nutrition, obesity, and other public health issues [29, 30].

### Participant characteristics

Altogether, 166 stakeholders took part in the workshops (for descriptive information see Table 1). Participants represented various food system stakeholders, including the food processing industry (22.7%), the scientific research sector (16.0%), the healthcare sector (12.7%, particularly clinical nutrition), food industry companies engaged in both production, technology development and food research (12.0%), young consumers aged 18–19 (8.7%), the food ingredients industry (6.7%), industrial agriculture and aquaculture (6.0%), retail and catering (5.2%), non-governmental organizations supporting consumer rights (3.3%), governmental agencies (3.3%, such as food industry regulators, commerce chambers, and consumer rights protection organizations), marketing (2.0%), and other sectors (1.4%). In each country, the workshops included representatives from at least three different sectors, except in Italy, where only food production sector stakeholders participated, and in Poland, where only young consumers participated in two workshops.

**Table 1:** Characteristics of workshops' participants in 13 countries

Country	*N*	*N* of participants who provided their data (% of original *N*)	Gender	Distribution of age (in years)	Years of work experience^a^
All countries	166	150 (and 93%)	39 M (26%); 111 F (74%).	18–25–13% 25–35–32% 36–45–27% 46–65–26% > 65–3%.	1–5–31% 6–15–31% ≥ 16–29% not provided – 9%
Austria	11	11 (100%)	5 M (45.4%); 6 F (54.5%)	26–35–27% 36–45–36% 46–55–36%	1–5–9% 6–10–9% 11–15–27% 16–20–45% > 20–9%
the Czech Republic	12	7 (58.3%)	1 M (14.2%); 6 F (85.7%)	18–25–14% 26–35–28% 36–45–28% 46–55–14% 56–65–14%	< 1–42% 1–5–14% 11–15–14% 16–20–14% > 20–14%
Denmark	13	6 (46.2%)	3 M (50.0%); 3 F (50.0%)	26–35–16% 36–45–33% 56–65–16% > 65–33%	1–5–33% 6–10–33% 11–15–16% > 20–16%
France	16	16 (100%)	16 F (100%)	18–25–6% 26–35–12% 36–45–50% 46–55–6% 56–65–25%	1–5–25% 6–10–12% 11–15–6% 16–20–25% > 20–31%
Germany	8	6 (75.0%)	2 M (33.3%); 4 F (66.6%)	18–25–16% 26–35–50% 36–45–33%	1–5–66% 6–10–16% 16–20–16%
Greece	10	10 (100%)	4 M (40.0%); 6 F (60.0%)	26–35–40% 36–45–20% 46–55–10% 56–65–30%	1–5–40% 6–10–10% 16–20–20% > 20–30%
Italy	13	13 (100%)	5 M (38.4%); 8 F (61.5%)	26–35–30% 36–45–38% 46–55–15% 56–65–7% > 65–7%	
Norway	9	9 (100%)	3 M (33.3%); 6 F (66.6%)	26–35–44% 36–45–22% 46–55–22% > 65–11%	< 1–11% 1–5–22% 6–10–33% > 20–33%
Poland	38	37 (97.3%)	5 M (13.5%); 32 F (86.4%)	18–25–43% 26–35–32% 36–45–13% 46–55–10%	< 1–21% 1–5–29% 6–10–21% 11–15–24% > 20–2%
Portugal	11	10 (90.9%)	2 M (20.0%); 8 F (80.-%)	36–45–20% 46–55–50% 56–65–30%	1–5–10% 11–15–10% 16–20–20% > 20–60%
Slovenia	7	7 (100%)	2 M (28.5%); 5 F (71.5%)	26–35–28% 36–45–57% 46–55–14%	1–5–42% 11–15–28% > 20–28%
Spain	10	10 (100%)	5 M (50.0%); 5 F (50.0%)	26–35–60% 36–45–10% 46–55–20% 56–65–10%	1–5 -60% 6–10–10% 11–15–10% > 20–20%
Turkey	8	8 (100%)	2 M (25.0%); 6 F (75.0%)	26–35–50% 36–45–25% 46–55–25%	1–5–25% 6–10–37% 11–15–37%

17 workshops were conducted, including 5 in Poland and 12 in each of the 12 remaining countries

*F* female gender, *M* male gender

a – the distribution excluding young consumers (18–19 years old; all students, not employed) participating in 1st and 2nd workshops conducted in Poland

### Data analysis: identifying common leverage points across 17 system maps

In the first step, the analysis aimed to identify leverage points, which are defined as system determinants that exhibit high structural influence and, therefore, have a high potential for future intervention. Network analysis, conducted with STICKE 3.0 software, was used to calculate values of four types of centrality indicators: (i) *eigenvector*, with high values representing the leverage points in the system; (ii) *degree*, with high-degree elements indicating the system elements that are sensitive to change; (iii) *closeness*, with high values representing resilient elements; and (iv) *betweenness*, with high values representing bottlenecks/gateways into the system [28]. The analysis focused on identifying the leverage points, that is determinants which are characterized by high eigenvector values [27, 28], accompanied by high values of closeness and betweenness (for similar approach see [22, 28]). Next, the leverage points (determinants with the highest values of eigenvector, but also high values of betweenness and closeness) were grouped into broader categories, representing higher-level shared characteristics.

### Analysis of feedback loops in system maps

In the second step, feedback loops were identified using KUMU software [32]. Specifically, we focused on identifying loops that included at least one leverage point and its associations with other determinants in the system. After the initial identification of all feedback loops, two approaches were taken: (i) summarizing all feedback loops in maps with < 50 loops and (ii) a reduction procedure applied in case of ≥ 50 loops. The reduction procedure aimed at decreasing the number of loops by extracting all loops that involved the ten variables with the highest values of centrality indicators (eigenvector, closeness, and betweenness), and by removing the variables with centrality indicator values lower than the ten highest values for any of the centrality indicators. This strategy resulted in obtaining feedback loops that provided information on how the leverage points, identified in each country, are linked with other determinants in the system map. Common patterns in the feedback loops were identified.

## Results

### Descriptive results

**Fig. 1:**
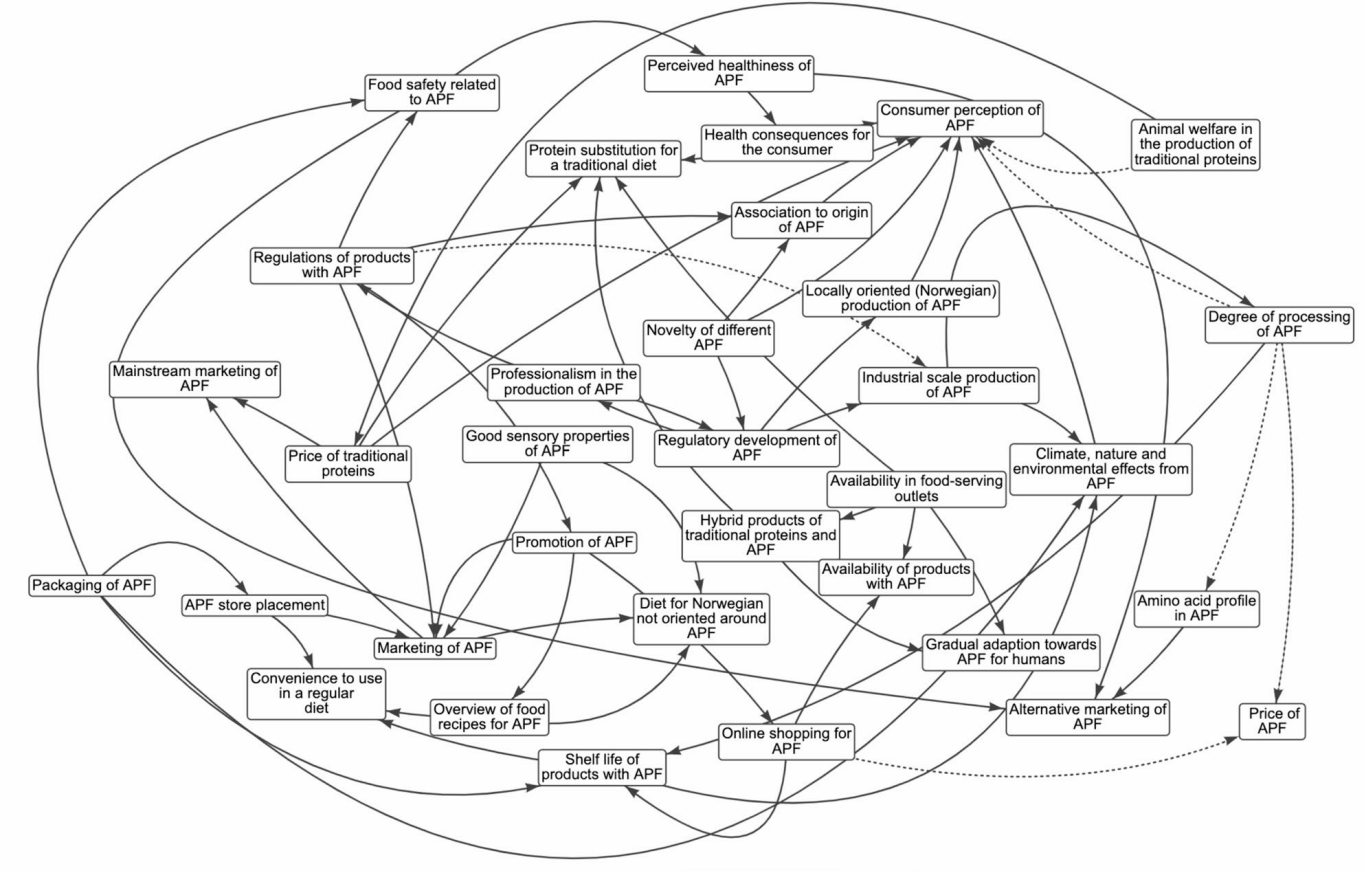
The system map of determinants of alternative protein food intake: the mapping workshop in Norway Note. Solid lines represent the positive edge; Dotted lines represent the negative edge. APF = alternative protein food. Definitions of determinants identified during system mapping workshops: Overview of food recipes for APF = recipes and tutorials on how to prepare APF meals; Association to origin of APF = implicit associations about the source of APF (e.g., insect powder -> unpleasant insects); Good sensory properties of APF = taste, smell and texture; Availability in food-serving outlets = availability of APF in food serving locations (e.g., restaurants); Alternative marketing of APF = tailoring marketing of APF for specific groups e.g., athletes; Regulatory development of APF = regular updates of APF product regulations

**Fig. 2:**
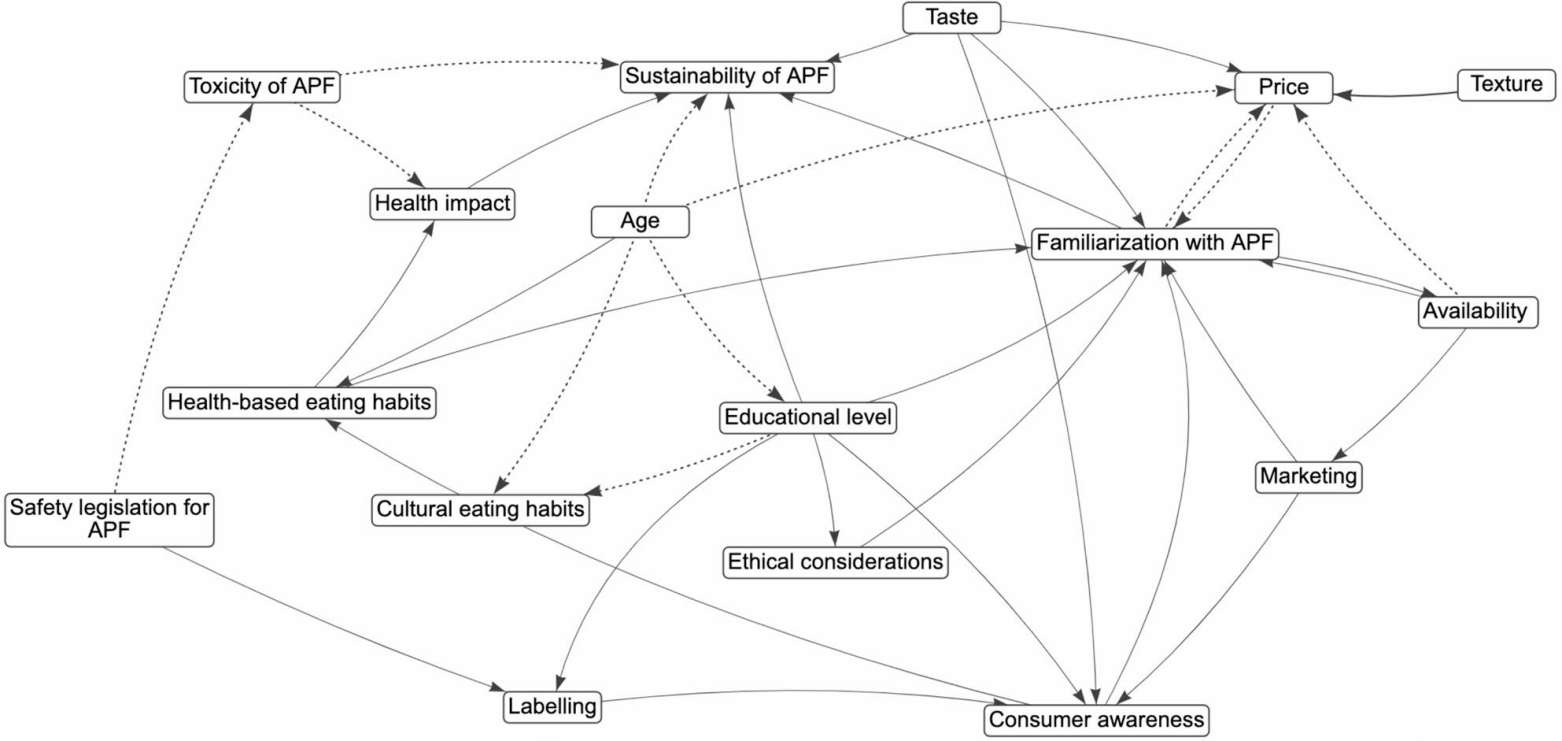
The system map of determinants of alternative protein food intake: the mapping workshop in Greece Note. Solid lines represent the positive edge; Dotted lines represent the negative edge. APF = alternative protein food; Ethical considerations = ethical considerations for animal welfare and environmental protection; Marketing = visibility of product promotions/attempts to overcome stereotypes; Familiarization with APF = the level of familiarity with APF products, in the context of cultural background; Sustainability of APF = the level of sustainability of the final APF product; Consumer awareness = consumers’ awareness of sustainability and health effects of APF; Health-based eating habits = eating restrictions that can limit individual’s food choices; Educational level = education about food; Labelling = information about sustainability on the label

**Fig. 3:**
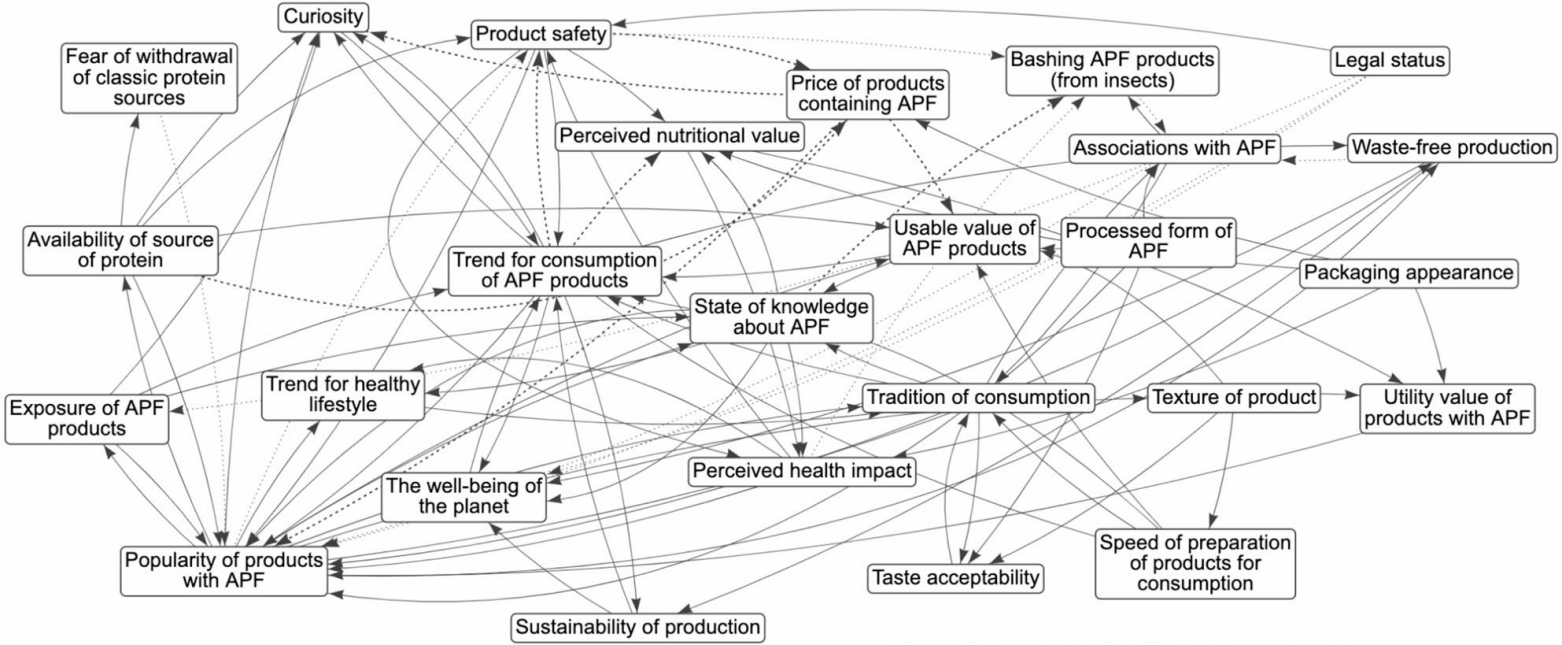
The system map of determinants of alternative protein food intake: The 2nd mapping workshop in Poland Note. Solid lines represent the positive edge; Dotted lines represent the negative edge. APF = alternative protein food. Definitions of determinants identified during system mapping workshops: Tradition of consumption = regional/national culinary traditions; Availability of source of protein = the availability of components to produce APF; Associations with APF = implicit associations about APF, e.g. insects -> pests; Popularity of products with APF = one person’s consumption of APF encourages others; Processed form of APF = un/processed sources of APF; Usable value of APF products = dishes that can be prepared from APF; Trend for healthy lifestyle = associating APF products with healthy lifestyle; Trend for consumption of APF products = the effect of influencers on the desire to buy APF; Packaging appearance = attractiveness of packaging; Texture of product = preference for a specific texture of APF product; Utility value of products with APF = knowledge of recipes with APF; Fear of withdrawal from classic protein sources = the concern that if APF becomes popular, it will limit the availability of traditional protein products; Bashing APF products (made from insects) = negative opinions/hateful comments regarding APF on social media; Exposure of APF products = exposure in TV/social media; Legal status = laws affecting supply, popularity, and safety of APF; Waste-free production = limited waste in APF production

**Fig. 4:**
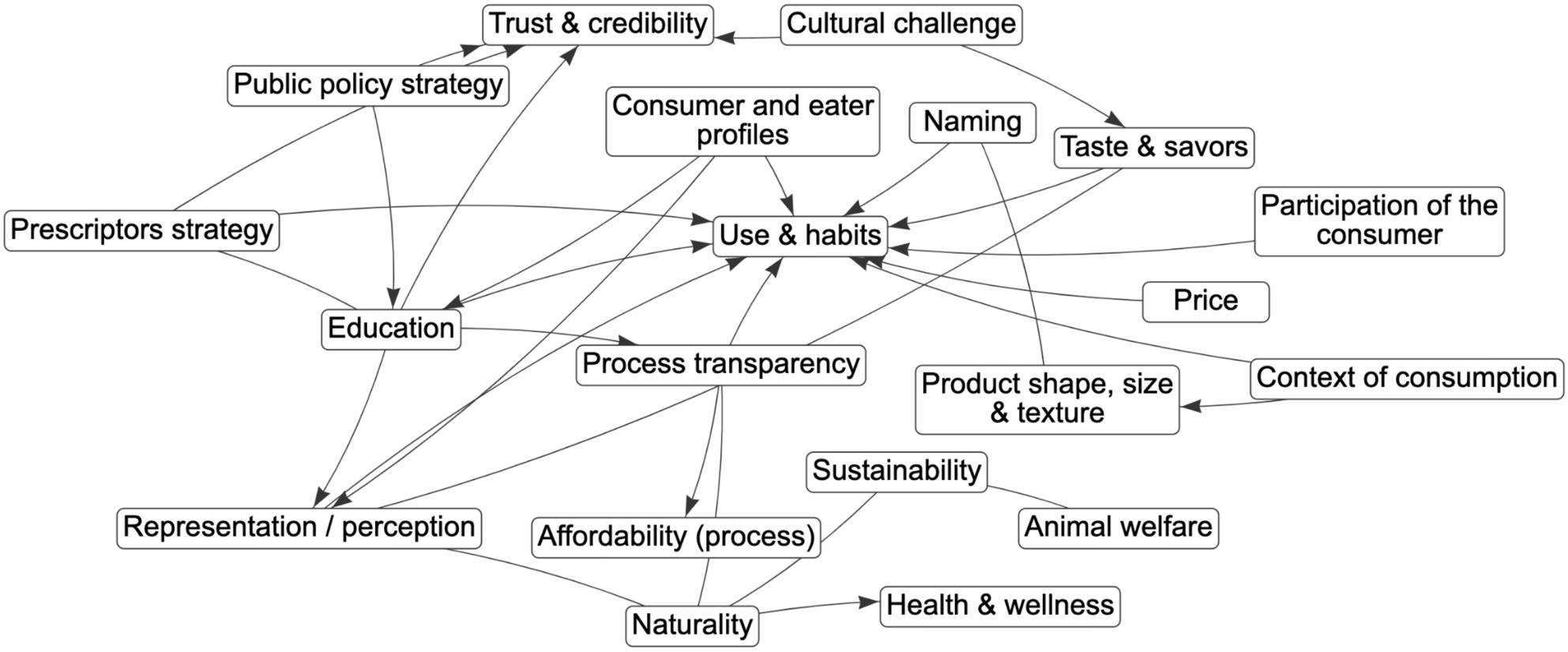
The system map of determinants of alternative protein food intake: the mapping workshop in France Note. Solid lines represent the positive edge; Dotted lines represent the negative edge. APF = alternative protein food, Definitions of determinants identified during system mapping workshops: Process transparency = explanation of how products are made step by step; Cultural challenge = knowledge of APF products within cultures favoring traditional proteins; Naming = names of APF products (e.g., a vegan sausage); Price = affordable prices; Affordability (process) = cost of the technology used in APF production; Public policy strategy = policies promoting APF/educating about APF; Use and habits = culinary habits; Education = knowledge on how to cook with APF; Sustainability = environmental impact of APF; Health and wellness = nutritional benefits of APF; Category management = shelf strategy for product placement; Trust and credibility = trust in producer/product; Participation of the consumer = consumer participation in a product creation; Context of consumption = places where APF are consumed; Consumer and eater profiles = specific consumer profiles, e.g., young people; Representation/perception = visual/perceptual representation of an APF product

The *k* = 17 maps of food system determinants of consumers’ APF choices (see Additional file 2) included 10–34 variables (total *N* = 336 determinants; *M* = 19.76; Austria = 12 determinants; the Czech Republic = 10; Denmark = 23; France = 23; Germany = 19; Greece = 17; Italy = 14; Norway = 34; Poland, first workshop = 23; Poland, second workshop = 17; Poland, third workshop = 26; Poland, fourth workshop = 20; Poland, fifth workshop = 17; Portugal = 21; Slovenia = 23; Spain = 23; Turkey = 14). Table S1 in Additional File 2 provides a complete list of determinants, their definitions, and system map. Examples of maps developed in Northern Europe (Norway), Southern Europe (Greece), Eastern Europe (Poland 2), and Western Europe (France) are presented in Figs. 1-4, respectively.

### Common leverage points identified across system maps

The determinants that were identified as leverage points are reported in Table 2 (for more details see Additional file 2 in Table S2). Each of the *k* = 17 maps included a unique set of determinants. However, several broader categories of leverage points were common across ≥ 3 system maps.

**Table 2:** Results of network analysis for system maps of determinants of consumers’ choices of alternative protein food: determinants included in 17 system maps with the highest eigenvector values (i.e., leverage points)

Country	Determinants with the highest values of the eigenvector centrality measure included in the system maps
Austria	Ingredient overload in APF products = 0.49; health effects of APF products = 0.49; nutritional factor of plant proteins (PER) = 0.41; ultra-processed foods = 0.41
the Czech Republic	Fear of the unknown = 0.58; price = 0.49; promotion and advertisement of APF = 0.45
Denmark	Family suitability = 0.49; Danish food culture = 0.38; knowledge of preparation = 0.38
France	Use and habits (cultural familiarity and tradition, family habits) = 0.51; education (on how to cook) = 0.45; visual representation /presentation/ perception of the APF product = 0.35
Germany	Familiarity (opposite to fear of new products) = 0.32; availability = 0.36; social norms = 0.36
Greece	Familiarization with APF (culture-related factor) = 0.47; educational level (low cognitive ability and rigidness in food choices) = 0.37; age = 0.26
Italy	Advertising = 0.43; curiosity = 0.38; environmental impact = 0.32; APF intake perceived as good and balanced diet = 0.32
Norway	Regulations of products with APF = 0.28; climate, nature and environmental effects of APF = 0.28; degree of processing of APF = 0.26
Poland (1st workshop)	Nutritional diversity (dietary habits rich in various types of proteins) = 0.35; popularity of APF (social encouragement, social norm) = 0.35; positive attitude towards (e.g., ethics of not eating meat) APF = 0.35
Poland (2nd workshop)	Educating consumers about APF = 0.44; norms of APF in terms of USP* = 0.37; social approval = 0.37
Poland (3rd workshop)	Popularity of products with APF (social encouragement, social norm) = 0.47; trend for consumption (due to influencers) = 0.32; knowledge about APF = 0.30
Poland (4th workshop)	Readiness for culinary variety = 0.37; disgust = 0.33; availability of APF = 0.32
Poland (5th workshop)	Dissemination of knowledge about APF = 0.33; normalization of the APF topic in mass media/social media = 0.32; concern for welfare of livestock = 0.26
Portugal	Trust (of consumers in APF) = 0.44; social trends (popularity of trying APF) = 0.40; literacy about APF = 0.36; nutritional profile of APF = 0.35
Slovenia	Taste = 0.39; influence of influencers = 0.30; advertising = 0.32
Spain	Promotion of consumption = 0.50; lack of knowledge = 0.37; perception of ultra-processed foods = 0.28
Turkey	Preference of APF due to health, environment and ethics = 0.49; marketing influencing consumer perception = 0.35; taste = 0.30

*APF* alternative protein food

*USP - a uniqueness of a product (unique selling proposition; the essence of what makes your product better than competitors)

#### Consumers’ knowledge and educating consumers about alternative protein food as leverage points

Stakeholders suggested that various aspects of knowledge about APF may influence its intake. In general, knowledge can be categorized as conceptual, procedural, and experiential [33, 34]. Conceptual knowledge refers to cognitive understanding of a product and its effects [34]. In the case of APF, it includes the ability to correctly identify APF, understand its nutritional value, and recognize the health consequences of consuming it, consistent with existing scientific evidence. Procedural knowledge, a subtype of conceptual knowledge, relates to the methods of using a product [34]. For example it refers to knowing how to cook or prepare a meal with APF. Experiential knowledge, in contrast, pertains to personal experience with a product, including its attributes or the consequences of its consumption [33]. This type of knowledge may encompass sensory memories, such as the smell or taste of a specific APF product that an individual has recently tried.

Determinants from the category referring to conceptual or experiential knowledge and educating the consumers were identified in *k* = 8 system maps: Denmark, France, Germany, Greece, 3x Poland, and Portugal (see Table 2 and Additional file 2). The definitions of these determinants indicate that stakeholders referred to limited APF literacy, low knowledge of how to prepare/cook APF, and low dissemination of knowledge about APF.

Familiarity with the product, which encompasses knowledge defined as personal experience with the attributes of a specific food, was also indicated on the maps developed in Germany and Greece. Knowledge of how to cook or prepare APF (e.g., as indicated in the Danish map), may be a determinant that encompasses both formal and experiential knowledge of APF. Actions aiming at dissemination of knowledge by means of education policies were identified as leverage points in two system maps in Poland.

#### Social encouragement and social norms as leverage points

This category includes determinants related to the approval of APF by important others (e.g., family, friends, admired models), beliefs that APF choices are important to family/friends, as well as perceptions of APF as popular and “trendy” among important others. These determinants were found in *k* = 7 system maps (Denmark, France, Germany, 3 x Poland, Portugal) (see Table 2 and Additional file 2). Beliefs about family habits were indicated in France and Denmark; social norms – in Germany; trends, defined as the popularity of trying APF among important others, were identified in the map developed in Portugal. Social encouragement to choose APF or perceiving eating APF as a social norm (a behavior popular among important others) was indicated in *k* = 3 maps developed in Poland.

#### Advertising, promotion, and actions of influencers as leverage points

The determinants included in this category refer to campaigns that encourage APF consumption (other than educational campaigns), delivered by individuals (e.g., influencers), organizations, institutions, or governments/local authorities. They were identified in *k* = 7 system maps (the Czech Republic, Italy, 2 x Poland, Slovenia, Spain, and Turkey). *K =* 4 maps included marketing, advertising, and/or promotion of APF as the key leverage points (the Czech Republic, Italy, Spain, and Turkey; Table 2 and Additional file 2). The promotion of APF by influencers was determined as a leverage point in *k =* 3 maps (Slovenia, and 2x Poland).

#### Perceptions of healthy, balanced, and safe alternative protein food as leverage points

The respective category of determinants refers to various health and safety issues, including consumers’ beliefs about healthiness, the actual nutritional content, and the regulations regarding the content of APF (*k* = 5 system maps; Austria, Italy, Portugal, Norway, and Turkey). Four system maps included leverage points related to the content of APF, which can be perceived as an example of choosing a healthier and balanced diet (Austria, Italy, Portugal, and Turkey). A lack of harm related to the product’s healthiness and safety may also be a key component of trust in producers of APF, which was classified as a leverage point in Portugal. Regulations around APF were indicated in a map from Norway.

#### Positive and negative emotions as leverage points

Leverage points referring to complex emotions were identified in four countries: the Czech Republic, Germany, Italy, and Poland. In particular, neophobia (the fear of novel or unknown foods) and curiosity about unknown foods were identified as leverage points (see Table 2 and Additional File 2).

#### Other common types of leverage points

*National food culture* and a presence or lack of APF in local cuisines were identified as leverage points in *k* = 3 system maps (Denmark, France, and Greece; Table 2 and Additional File 2). Categorizing APF products *as ultra-processed foods* was identified as a leverage point in *k* = 3 system maps, developed in Austria, Norway, and Spain. Leverage points referring to the *environmental benefits* of including APF in the daily diet were identified in *k* = 3 system maps (Italy, Norway, and Turkey; Table 2 and Additional File 2). Finally, the leverage points referring to *concerns about animal welfare or ethical issues* (e.g., animal suffering) related to the consumption of traditional proteins were identified in *k* = 3 system maps (2 x Poland and Turkey).

### Results of feedback loops analysis

The maps developed in the five countries (the Czech Republic, Denmark, France, Portugal, and Slovenia) included unidirectional connections only, with no feedback loops. In the *k* = 3 system maps (Norway, 1 x Poland, and Spain), only one feedback loop per country was identified, each with two connections only (i.e., from A to B and from B to A). The remaining system maps, developed in six countries (Austria, Germany, Greece, Italy, 4 x Poland, and Turkey), included at least three feedback loops. Across the feedback loops identified in six countries (Austria, Germany, Greece, Italy, Poland, and Turkey), we found that specific chains of associations were common to at least half of the feedback loops.

The full list of the feedback loops identified in each of 13 countries is reported in Additional file 2, Tables S3–S14. All feedback loops include at least one leverage point (see Table 2 and Additional File, Table S2) and other determinants, as well as connections between them, as proposed by stakeholders in each respective country.

#### Feedback loops in Austrian map

The majority (4 out of 5) of feedback loops included variables (overload with ingredients, ultra-processed character, healthiness, and being curious about novel food) linked in a specific order. A high number of ingredients in APF was perceived by the stakeholders as related either to (i) perceiving APF as ultra-processed foods, and/or (ii) perceiving the food as of low health value. These two determinants (ultra-processed and unhealthy character) were, in turn, perceived as associated with lower curiosity and interest in trying something new. Next, stakeholders believed that low curiosity about novel foods could be related to perceiving APF as overloaded with ingredients and low interest in trying new foods. The feedback loops were reinforcing (two negative connections, with the remaining connections positive). Reinforcing feedback loops represent a mechanism of a downward spiral that hinders consumer interest in APF and reinforces low intake of respective foods.

#### Feedback loops in German map

The system map involved feedback loops in which availability and social norms (or the presence of social pressure to try APF) were linked to acceptance of APF. In particular, German stakeholders suggested that higher availability of APF may prompt consumers to consider that eating APF may be a social norm (both approved and popular among important others). Stakeholders also noted that these social norms could, in turn, contribute to greater acceptance of APF. Across the feedback loops identified in the German system maps, 50% included the respective sequence (availability-> social norm-> acceptance), representing a reinforcing feedback loop that may promote APF intake.

#### Feedback loops in Greek map

3 out of 4 feedback loops identified in the Greek system map suggest three sequentially connected variables. Greek stakeholders linked greater familiarity with certain products, such as specific types of legumes, with greater availability of alternative protein products based on respective plants. This, in turn, was seen as supporting product marketability and efforts to popularize the products. Marketing efforts were perceived as related to higher consumer awareness of a variety of APF products or consumers’ familiarity with APF. Finally, Greek stakeholders indicated that such awareness and familiarity could promote the increased availability of various APF products. These connections formed reinforcing feedback loops that represent a potential upward spiral, which contributes to an increase in APF intake.

#### Feedback loops in Italian map

Half of the feedback loops in Italian maps involved a potentially reinforcing upward spiral that promoted increased consumption of APF. According to Italian stakeholders, higher food safety of APF products may facilitate advertising of APF (e.g., as valuable foods). This, in turn, may increase consumers’ curiosity or prompt beliefs that APFs are the healthy choices, and promote consumers’ preference for food choices that may have a lower impact on the natural environment and/or a higher impact on local development and job creation. According to stakeholders, these dynamics may subsequently contribute to lower food prices and further advances in food safety.

#### Feedback loops in Poland’s map (workshop #2)

10 out of 12 feedback loops included yet another reinforcing upward spiral that was identified as a determinant prompting APF intake. In particular, Polish stakeholders suggested that further research on APF products, addressing safety and nutrition qualities, for instance, may increase product shelf life and/or facilitate marketability. A long shelf life and/or higher marketability could, in turn, positively affect influencers’ interest in APF and/or consumers’ curiosity and desire to try novel, alternative, protein-based foods. Finally, stakeholders suggested that consumers’ and/or influencers’ interest, in turn, may intensify research to further develop/improve APF products.

#### Feedback loops in Turkish map

All feedback loops included in a system map developed in Turkey featured a reinforcing upward spiral, which could potentially increase APF intake. The participants connected well-developed marketing and promotional strategies to changes in social and cultural narratives around APF products. These shifts were, in turn, linked to changes in individual beliefs about the benefits of an alternative, protein-based diet, specifically with regard to health, environmental sustainability, and animal welfare. Participants also noted that favorable consumer perceptions could facilitate continued marketing and promotional efforts, creating a reinforcing cycle.

## Discussion

This study provides novel insights into the network of determinants of APF intake. These insights were obtained by conducting system mapping workshops with food system stakeholders from 13 European countries. The findings allow us to draw conclusions regarding the key leverage points in the system of determinants of APF choices, as well as the chains of associations that were common components of feedback loops, identified in the respective countries.

The maps developed across countries are characterized by a high degree of heterogeneity among determinants, differences in connections between determinants, and a variety of feedback loops that may either balance the system or push the respective systems towards a change in APF uptake. These system maps can be considered case studies that provide information about the interplay of determinants which represent certain stakeholder perspectives in a given country. However, it should be noted that some types of leverage points were common across several system maps and appeared across different geographical regions of Europe. In particular, the three most frequently indicated leverage points –knowledge, social norms/social encouragement, and advertising approaches – were found in maps developed in Northern, Eastern, Southern, and Western European countries.

### Leverage points: experiential knowledge, “normalizing” and advertising APF intake

Regarding knowledge, it is important to note that, in addition to cognitive understanding of what APF is and its health and environmental consequences, stakeholders emphasized experiential and procedural knowledge of APF. In particular, knowing how to cook or prepare a meal with APF emerged as a relevant leverage point. Cooking with APF reflects both procedural knowledge (knowing how to use the product) and experiential knowledge (personal exposure through trying and tasting APF). This finding aligns with systematic reviews indicating that familiarity with APF and cooking/preparation skills are significant individual-level determinants of consumer choices, whereas conceptual knowledge plays a less substantial role [17, 35, 36].

The second type of leverage points refer to positive social norms, social approval of APF intake, and encouragement by important others (i.e., important role models, friends, and family who approve of APF and are willing to consume APF themselves). These social determinants highlight the importance of behaviors and beliefs of members of close social networks. Systematic reviews [17, 36] provided strong evidence of the links between social norms and consumers’ choices of APF. For example, encouragement from an important other (e.g., a romantic partner) may prompt unwilling young consumers to try new APF in restaurants [37].

The third type of leverage points, which obtained the most support in our study, refers to encouraging APF intake through advertising, promotion, and influencer marketing. The focus on the need to advertise APF products, along with the focus on knowledge-related factors and the social normalization of APF (perceiving others as APF consumers), may indicate that stakeholders’ assumed awareness of APF as viable protein source is low among European populations. Research on several types of APF (e.g., algae-based products) suggests that many Europeans may be unaware of this type of food [38]. Although awareness is not a central concept in health behavior change research, recent theoretical approaches and studies propose that *being aware* – observing oneself and noticing internal and external stimuli while allowing oneself to experience them – may serve as a focal point for shifting behavior from automatic to novel, autonomously motivated actions [39].

The findings regarding knowledge of APF also have practical implications. APF awareness campaigns and educational programs may need to include activities that facilitate procedural and experiential knowledge, that is, information on how to prepare a meal with APF, combined with APF sensory experience (observing and trying APF). These campaigns should also demonstrate approval of the APF intake by local influencers and relevant role models. Such interventions may have the potential to trigger a change in food system determinants.

### Feedback loops: linking individual-level, social, and environmental micro-, meso-, and macro-level determinants

One of the key characteristics of the chains of associations identified in this study is the connection of determinants from different levels. The feedback loops included determinants referring to either the social or physical environment, or both, as well as individuals’ characteristics. Several feedback loops are also linked to determinants from the micro-, meso-, and macro-levels. For example, feedback loops found in the system map developed by the Italian stakeholders linked individual, micro-level factors (such as consumer curiosity and orientation towards sustainable and local economy reinforcing products) with macro-level social factors (such as market/industry/retail responses of lowering prices, intensified advertising), as well as physical food environment factors (such as changes in food safety).

A similar example of cross-level links and the inclusion of social and physical environmental factors was identified by Polish stakeholders: individual-level factors (consumers’ curiosity) were linked to meso-level social factors (research on APF safety and quality), which were then linked to physical environmental factors (changes in food shelf life). These cross-level links of psychological, social, and physical environment-related determinants support the core assumptions of socio-ecological models, highlighting that a valid prediction of a health behavior should account for multi-level social and physical environment context, not just within-individual processes [13–15].

The results regarding feedback loops suggest that the availability of APF is a crucial element of potential upward spirals, which may increase intake of APF. Feedback loops identified by Greek stakeholders link consumers’ experience of familiarity with APF (a psychological variable at the individual level) and with the availability of APF (a factor of the physical environment), which, in turn, increases marketability of APF and marketing efforts (a social-environmental factor at the meso-level). Similarly, German stakeholders link the availability of APF with meso-level social-environmental factors, such as social pressure, approval, and popularity of APF, among others. These factors, in turn, were related to higher acceptance of APF (an individual-level factor). Previous research on the characteristics of the physical environment indicates that the actual availability of APF in local groceries, supermarkets, and restaurants is a crucial determinant of the intention to try, buy, and include APF into one’s daily diet [37]. The present study is the first to propose a chain of associations that link availability to individual-level beliefs and meso-level social factors.

The majority of the feedback loops identified in the present study were classified as reinforcing feedback loops, or the upward spirals, that have the potential to cause positive changes in the food system in terms of the broader uptake of APF. Across the European countries, stakeholders participating in system mapping workshops identified potential ways forward that could be pursued by various actors within food systems, including policy makers (e.g., by proposing food availability regulations), non-governmental organizations (e.g., by using social pressure instruments in social media), food marketing corporations (e.g., by intensifying APF marketing efforts), and novel food developers (e.g., by researching determinants of food safety).

Two system maps (Austria and the first map developed with adolescents in Poland) included downward spirals indicating how determinants operating within the food system may lead to a decline in APF intake, APF production, and APF availability. Furthermore, although most feedback loops were formulated by stakeholders as chains of determinants that promote APF intake, they can also be interpreted as explaining why APF is currently widely *not* adopted by consumers. For example, the chain of associations identified in the German system map suggest that lower availability of APF may be associated with a further decline in social approval of APF and perceptions that consuming APF is not something significant others would do (i.e., weak social norms), which in turn may contribute to lower acceptance of APF. This interpretative approach may help explain challenges faced by the food sector with respect to APF production and sales. For example, it may clarify how narratives promoted by high-profile individuals—suggesting that APF is something that “nobody important would eat” or expressing disapproval of APF—may contribute to lower consumer acceptance of APF and consequently reduced pressure from stakeholders to make APF more widely available.

The Austrian and Spanish system maps included perceptions of APF as ultra-processed foods as leverage points, hindering APF intake. Plant-based, microbial-based, and other APFs undergo extensive refinement and include processed ingredients to replicate texture, taste, and appearance of traditional protein food [40]. Therefore, some food classifications consider APF an example of ultra-processed food, which may result in consumers’ reluctance to include APF into a daily diet. However, recently, food researchers suggested that classifying APF as a typical ultra-processed food is incorrect [40]. Many APF products, such as plant-based meat and dairy alternatives, offer high nutrition quality, similar or superior to unprocessed animal products [40]. The notion of ultra-processed food was originally developed as a category encompassing fast food, sweet and salty snacks high in saturated fat and sugar, or ultra-processed beverages, all of which have been proven to be associated with poor cardiometabolic health outcomes [40]. Due to their nutritional and health value, APF should not be included in the original category of ultra-processed foods. Instead, different types of processed foods should be grouped separately depending on their nutritional and health profiles. Respective educational actions targeting consumers and other stakeholders are needed.

The findings related to the identified feedback loops may support food system stakeholders in the development of policy-relevant interventions. For instance, these interventions could incorporate the strategic use of influencers to increase consumer familiarity with APF, raise awareness of their health and safety attributes, and frame APF consumption as socially acceptable and positively valued. APF promotion campaigns that are endorsed and discussed by popular influencers may consequently contribute to increased consumer demand for APF and, in turn, incentivize retailers to expand the availability of APF in local grocery stores and supermarkets.

Our study does not provide evidence regarding which stakeholders may be best positioned to act on the identified leverage points. However, previous research on interventions and policies aimed at changing dietary patterns suggests that multi-stakeholder engagement increases the likelihood of successful implementation, broad reach (e.g., in vulnerable communities), and long-term sustainability [41]. Therefore, diverse food system stakeholders—such as policymakers, consumers, retailers, clinical nutrition specialists, and producers—should collaborate to develop a consensus on how to act on key leverage points, that have a potential to trigger a broader uptake of APF.

### Between-country differences in system maps

As the study was conducted in 13 countries, the findings might initially be interpreted as reflecting cross-country differences. However, it is likely that the observed differences across system maps primarily result from the composition of workshop participants. Each workshop had a distinct participant profile: for example, stakeholders in Italy were predominantly producers, whereas the first and second workshops in Poland included mainly young consumers. The content of the system maps, including the identified leverage points and feedback loops, is influenced by the participants’ professional experience. Consumers are likely to identify different leverage points than producers: the latter may be most likely to identify barriers related to developing new safe APF, meeting all respective food safety regulations in a country.

Differences between maps are also shaped by participants’ personal experiences. Even within the same country and among participants with similar professional backgrounds, variation can be substantial. For instance, the third, fourth, and fifth workshops conducted in Poland involved mostly clinical nutritionists, yet the resulting maps differed significantly in terms of identified leverage points and feedback loops.

Therefore, between-map differences should be interpreted with extreme caution, as multiple factors may contribute to these variations. Instead, interpretations and conclusions may more reliably focus on similarities observed across maps, such as recurring leverage points.

### Limitations

The system maps developed in this study were of remarkably high heterogeneity in terms of the included determinants, their connections, and the identified leverage points. Within-country differences were also present, as suggested by the findings obtained from the five workshops conducted in Poland. The chosen approach of system mapping data analysis, which focused on calculating eigenvector centrality indices, has its limitations and provides no insight into more complex associations. These limitations were partially reduced by a qualitative analysis of feedback loops, which better represent the system characteristics and its non-linear, circular associations [42].

The system mapping approach reflects the knowledge, beliefs, and experiences of the stakeholders who participated in the workshops. Small samples and a lack of representativeness of the stakeholders (for the overall national networks of food system stakeholders) are another limitation that hinders any generalizations. However, system mapping may be considered a first step toward more complex research that could ultimately inform policy and intervention practices. The findings for 17 system maps presented in this study focus on similarities between countries. However, national-level policies and interventions should be informed by within-country findings, which were not analyzed systematically in this study.

Our study focuses on the empirical analysis of a complex system, using APF intake as the key entry point. This allows us to understand the interrelations between the various elements of the food system. However, this approach has limitations because other relevant “entry points”, such as the actions of food producers, were not investigated. Future research should broaden this perspective by investigating determinants of producers’ choices to increase the production of APF, for example. Furthermore, translating our findings into practice requires further research to elucidate the key actions of other food system stakeholders (e.g., food producers and processors, retailers, marketing specialists, and policy makers), that may constitute leverage points to facilitate a system change. Addressing the leverage points that represent the actions of *all* food system stakeholders may substantially increase the likelihood of a food system transformation. This transformation is characterized by a broader uptake of APF by everybody and everywhere, followed by maintenance of new food habits (represented by common choices of APF) among consumers.

## Conclusion

This study provides new insights into the complex systems of the determinants of APF choices. 17 system maps highlight the role of individual-level consumer characteristics, as well as meso-level and macro-level social and physical environmental determinants of APF intake. Changes in these determinants may trigger reverberating effects occurring throughout the system. Therefore, the leverage points identified in this study are potentially the most promising targets for interventions promoting APF. Stakeholders participating in this system mapping study identified several feedback loops that may encourage incorporation of alternative protein foods into daily diets. These feedback loops may inspire food system stakeholders, including food producers, marketing specialists, researchers, and consumers across Europe, to develop novel interventions and policies that drive changes in APF intake.

## Supplementary Information

Supplementary Material 1.

Supplementary Material 2.

## Data Availability

Data (system maps and definitions of included determinants) are included in the supplementary files.
