# Supplementary material for: Mapping determinants of alternative protein food intake across 13 European countries: food system stakeholders’ perspectives
Source: Int J Behav Nutr Phys Act. 2026 Feb 19;23:28. doi: 10.1186/s12966-026-01891-3 (PMC13032592; doi:10.1186/s12966-026-01891-3)
Supplement: Supplementary file 1 — Supplementary Material 1. [file 12966_2026_1891_MOESM1_ESM.docx]

**Mapping Determinants of Alternative Protein Food Intake Across 13 European Countries: Food System Stakeholders’ Perspectives**

**Additional File 2**

Additional File 2 includes the following sections:

- Maps developed during system mapping workshops in 13 countries (Figures S1–S17)
- Definitions of all determinants included in maps from 13 countries (Table S1)
- The leverage points identified in network analysis in respective countries (Table S2)
- Feedback loops in system maps identified in each country (Tables S3–S15)

**Figure S1**

*Map Developed During System Mapping Workshops in Austria*


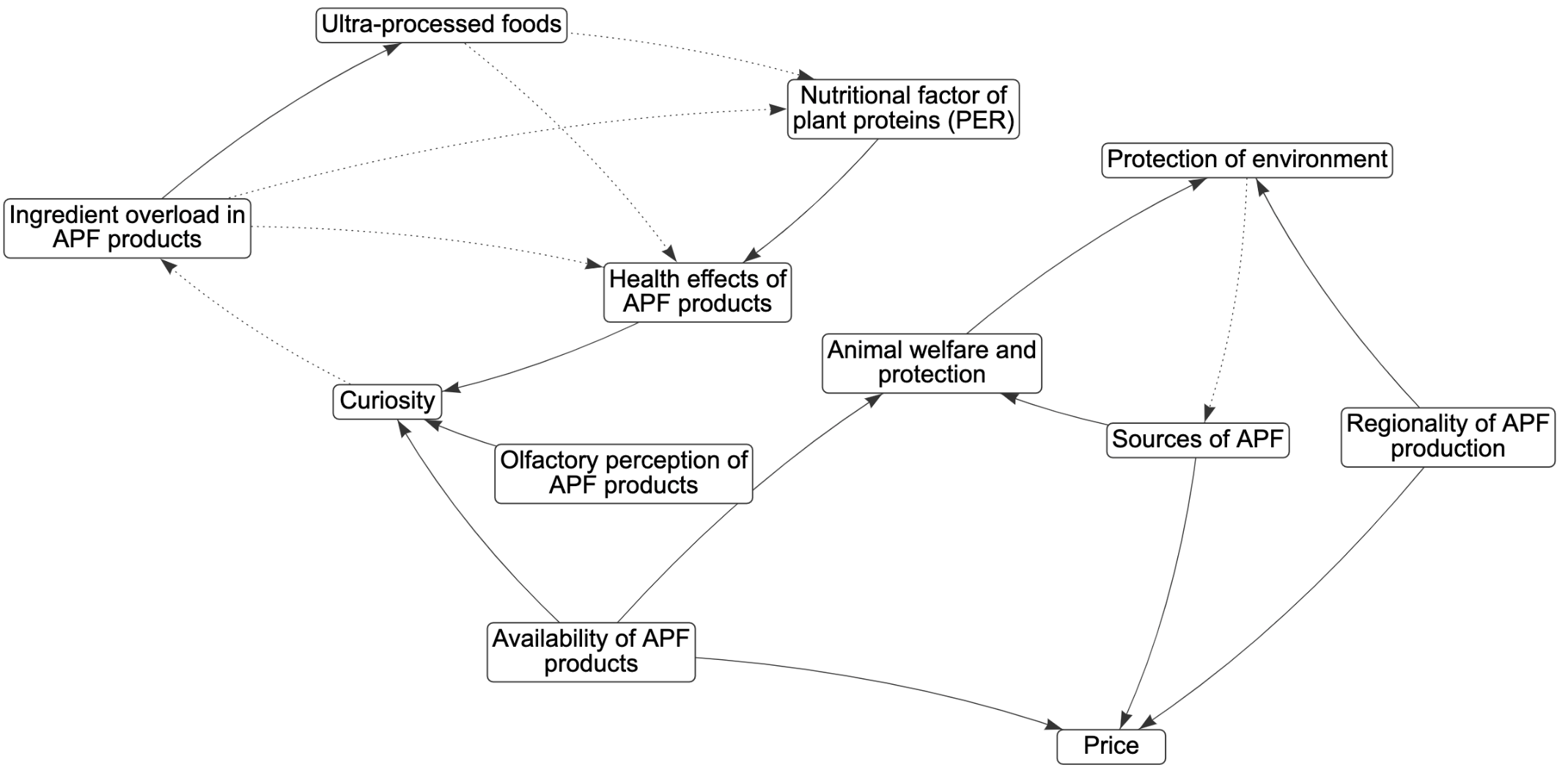


*Note.* Solid lines represent the positive edge; dotted lines represent the negative edge.

**Figure S2**

*Map Developed During System Mapping Workshops in the Czech Republic*


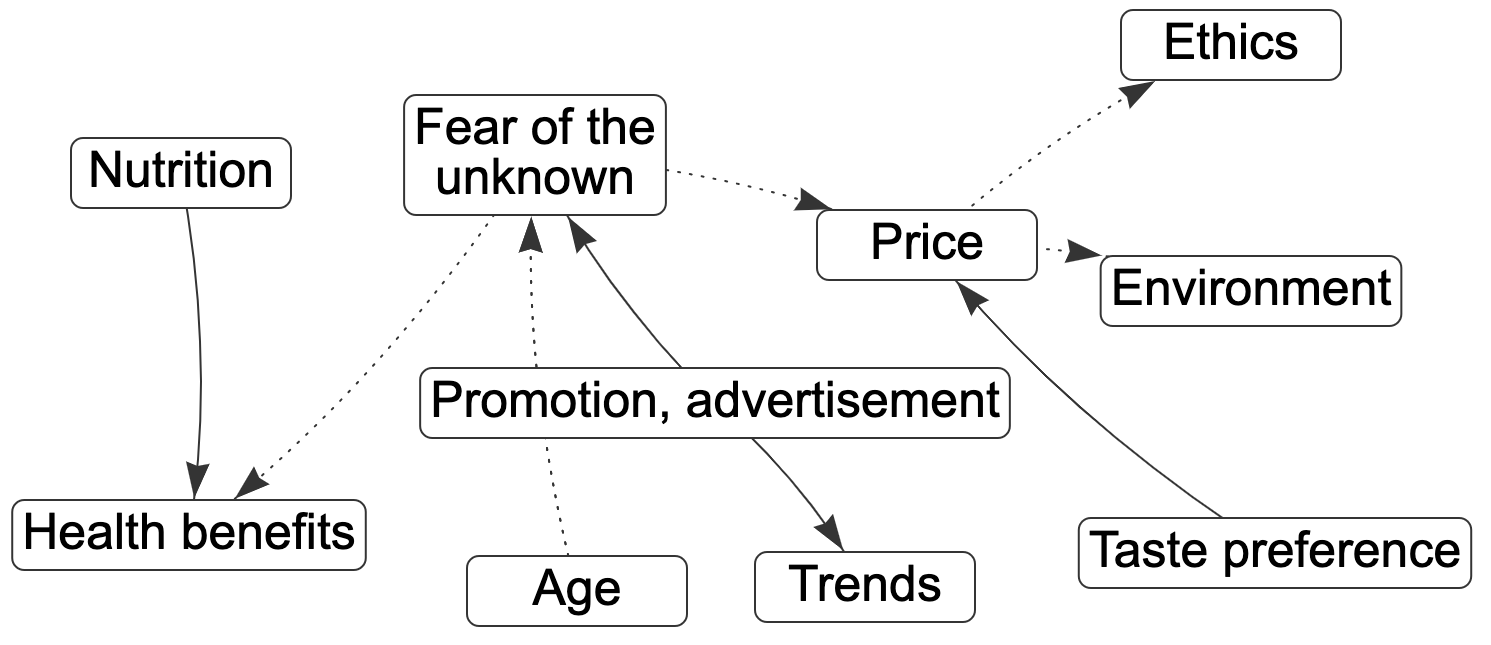


*Note.* Solid lines represent the positive edge; dotted lines represent the negative edge.


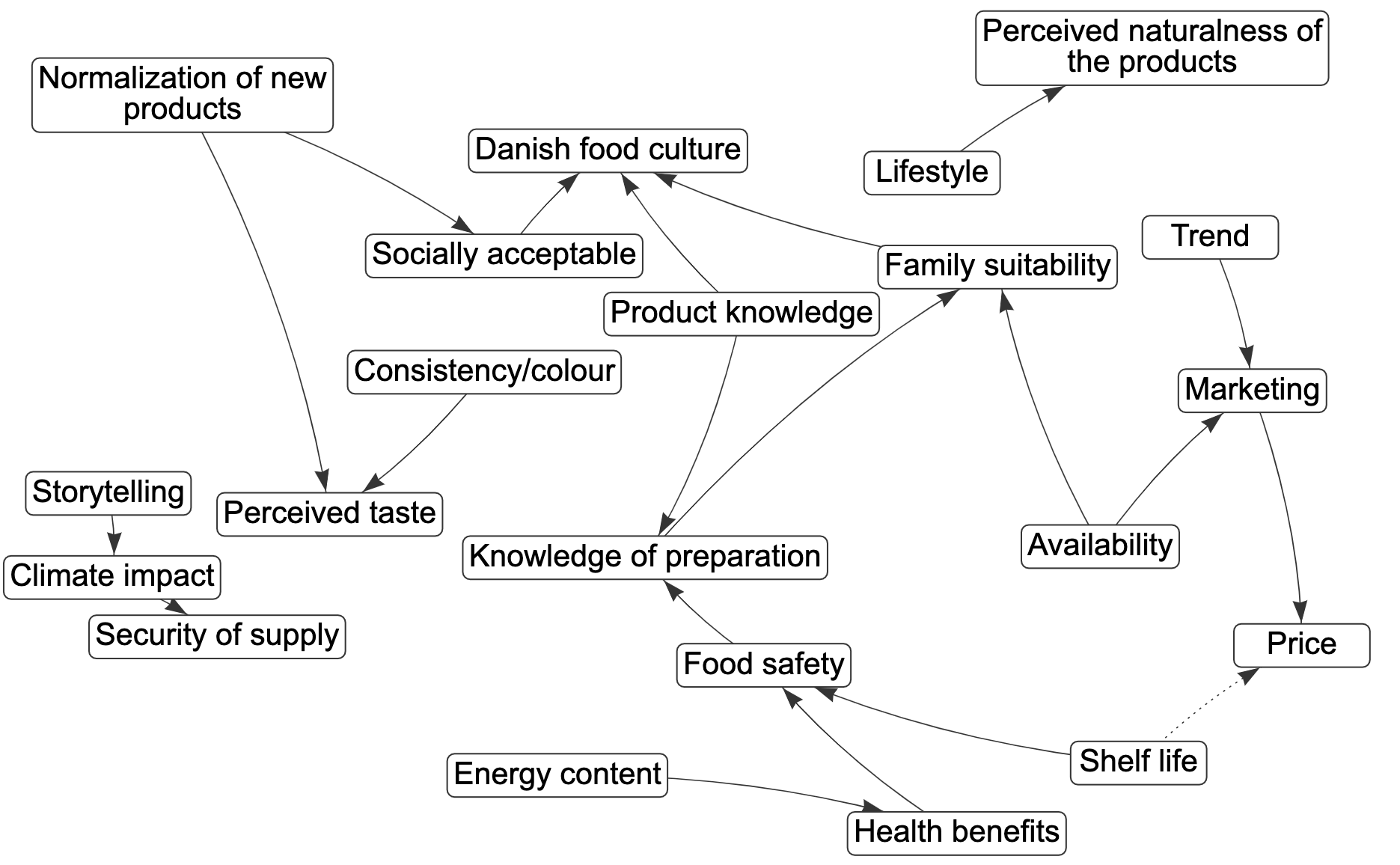
**Figure S3**

*Map Developed During System Mapping Workshops in Denmark*

*Note.* Solid lines represent the positive edge; dotted lines represent the negative edge.

**Figure S4**

*Map Developed During System Mapping Workshops in France*

*
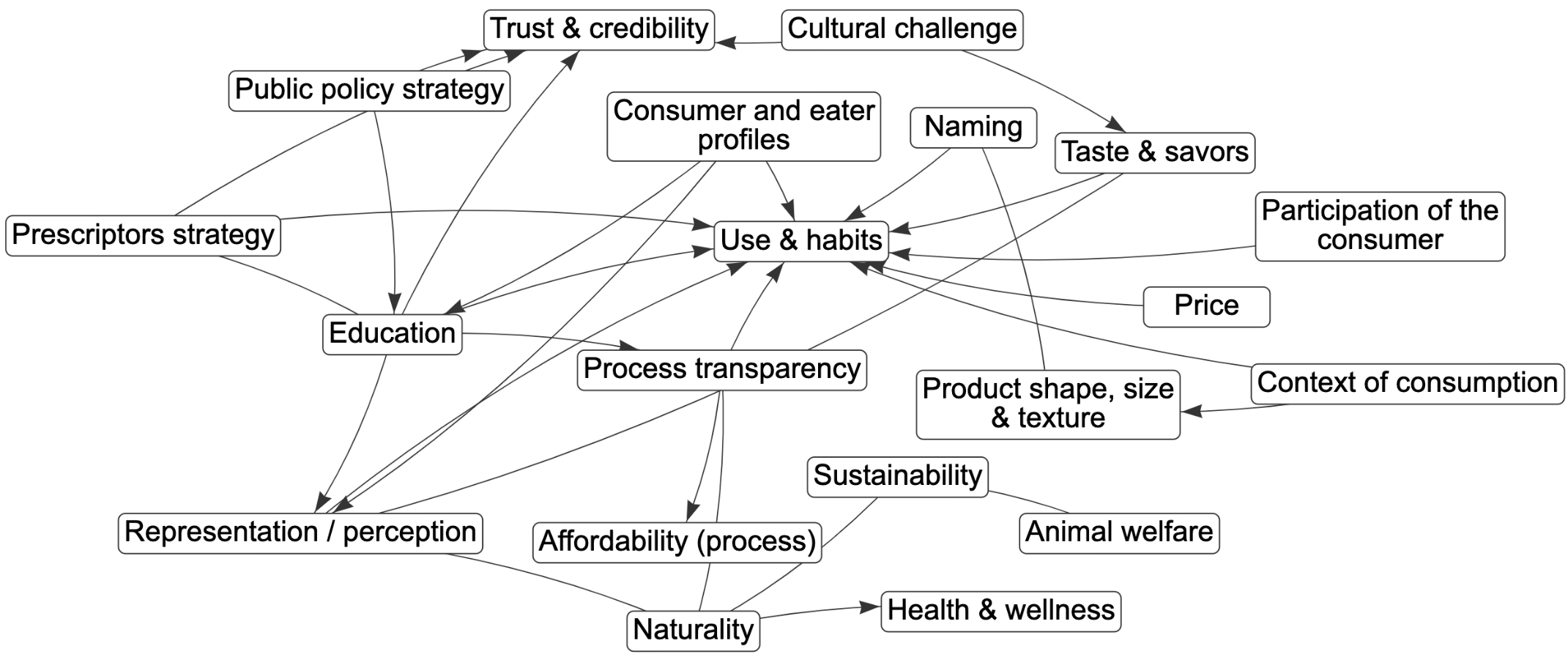
*

*Note.* Solid lines represent the positive edge; dotted lines represent the negative edge.

**Figure S5**


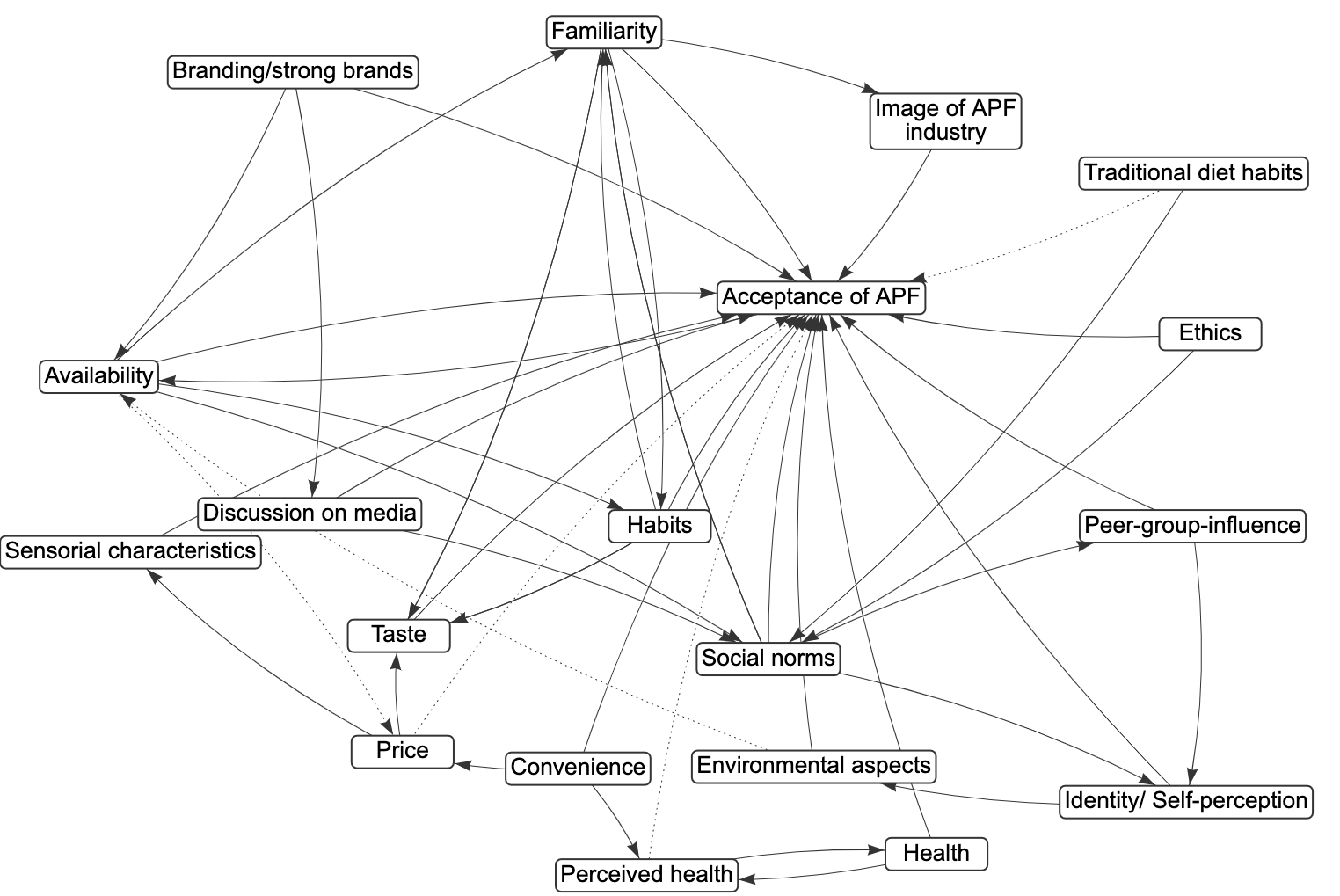
*Map Developed During System Mapping Workshops in Germany*

*Note.* Solid lines represent the positive edge; dotted lines represent the negative edge.

*Note.* Solid lines represent the positive edge; dotted lines represent the negative edge

**Figure S6**

*Map Developed During System Mapping Workshops in Greece*


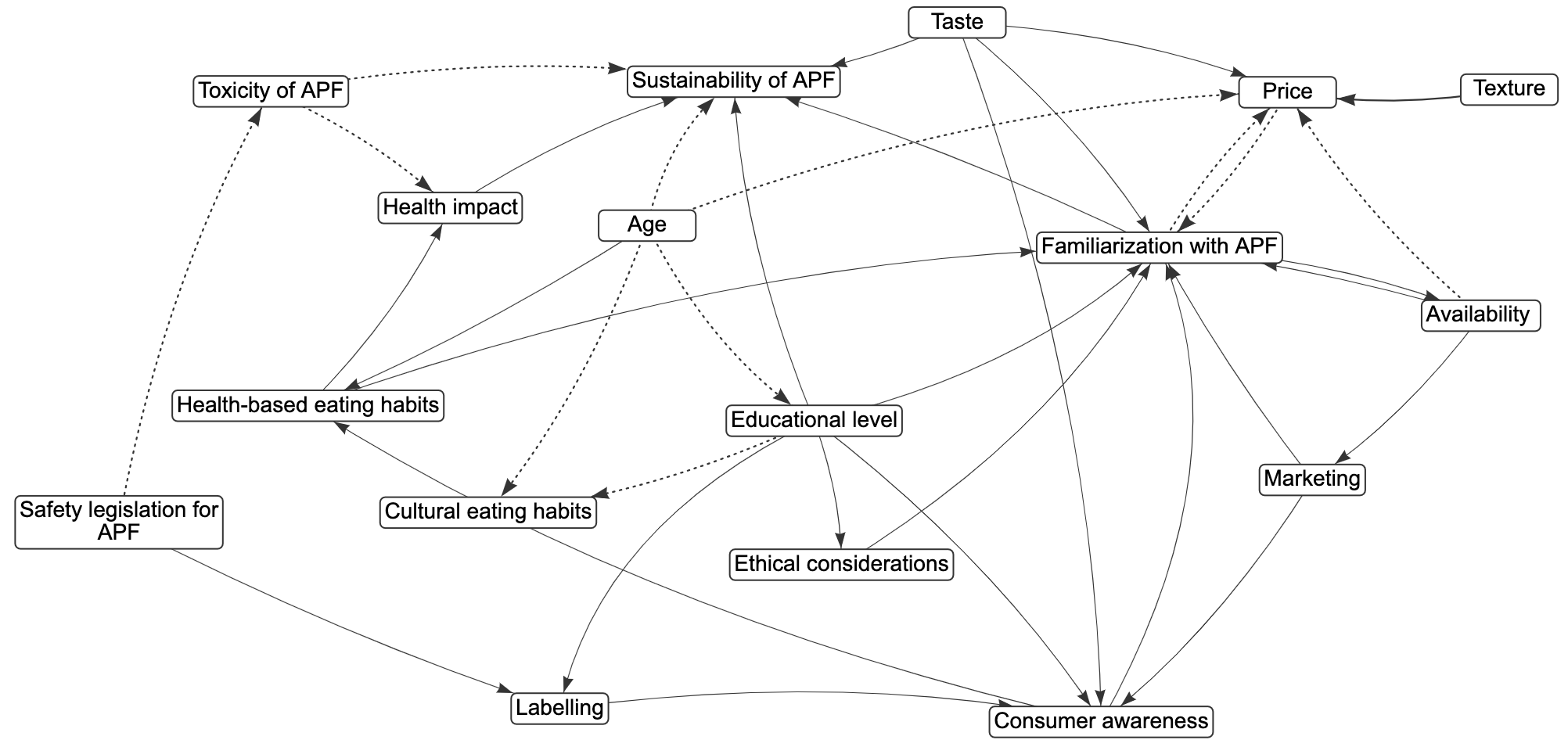


*Note.* Solid lines represent the positive edge; dotted lines represent the negative edge.

**Figure S7**

*Map Developed During System Mapping Workshops in Italy*


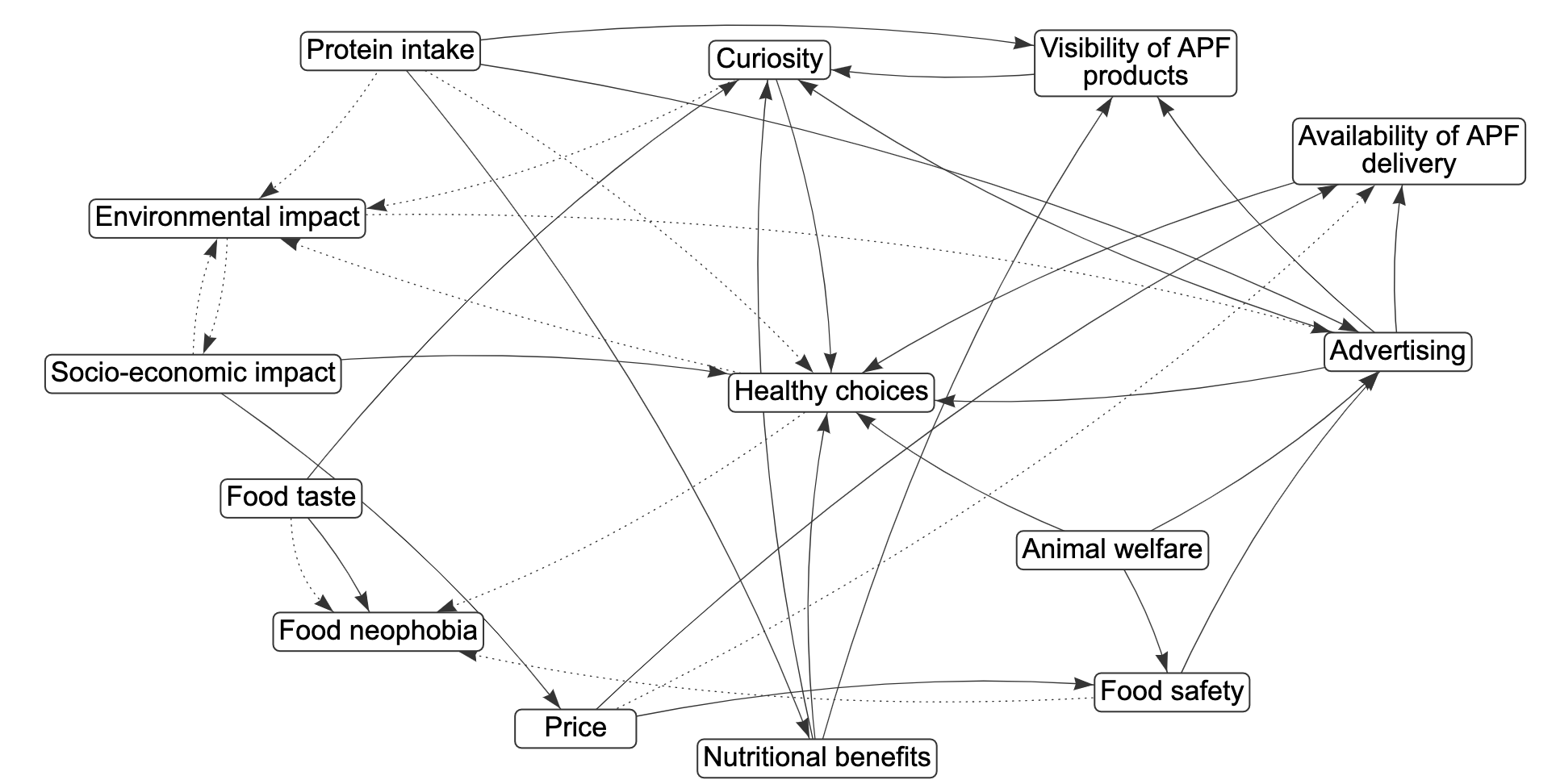


*Note.* Solid lines represent the positive edge; dotted lines represent the negative edge.

*
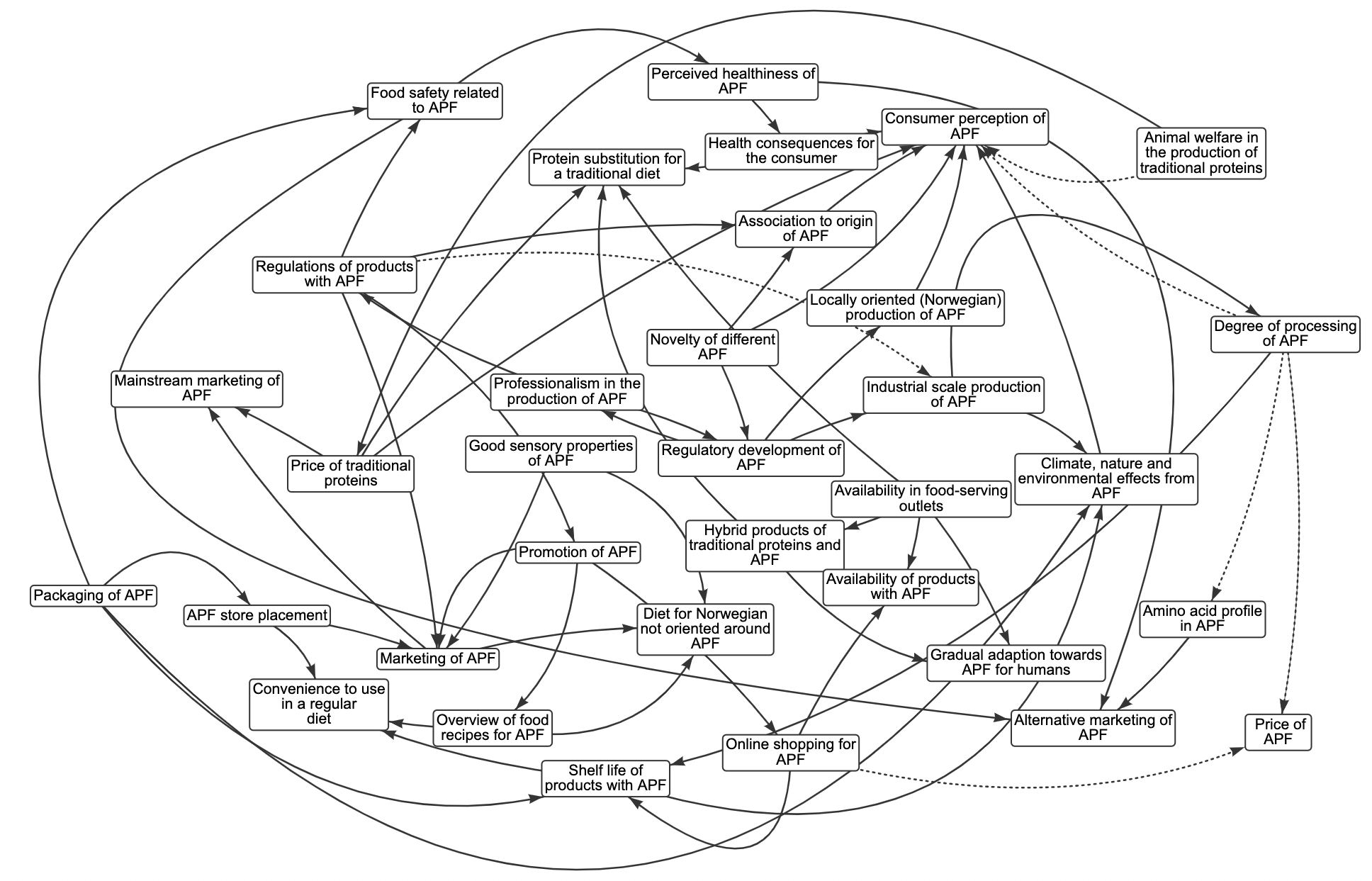
***Figure S8**

*Map Developed During System Mapping Workshops in Norway*

*Note.* Solid lines represent the positive edge; dotted lines represent the negative edge.

*Note.* Solid lines represent the positive edge; dotted lines represent the negative edge

**Figure S9**

*Map Developed During 1^st^ System Mapping Workshops with Adolescents in Poland*


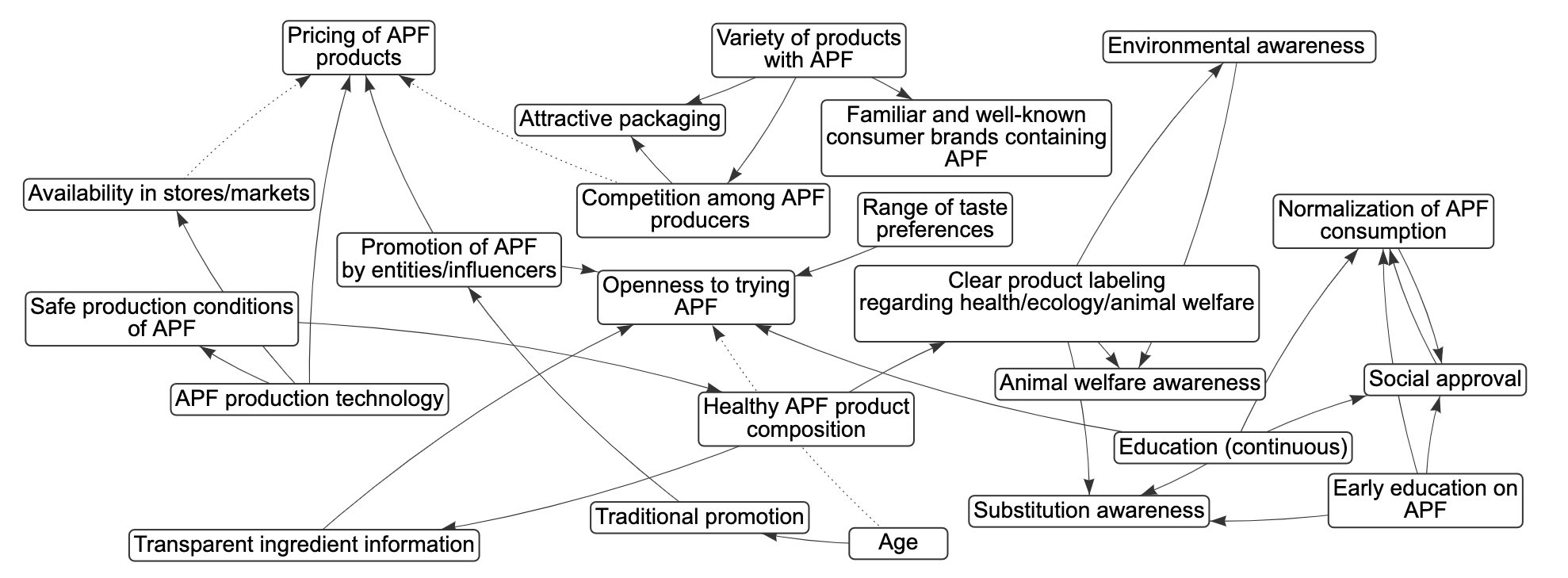


*Note.* Solid lines represent the positive edge; dotted lines represent the negative edge.

**Figure S10**

**Figure S10**

*Map Developed During 2^nd^ System Mapping Workshops with Adolescents in Poland*


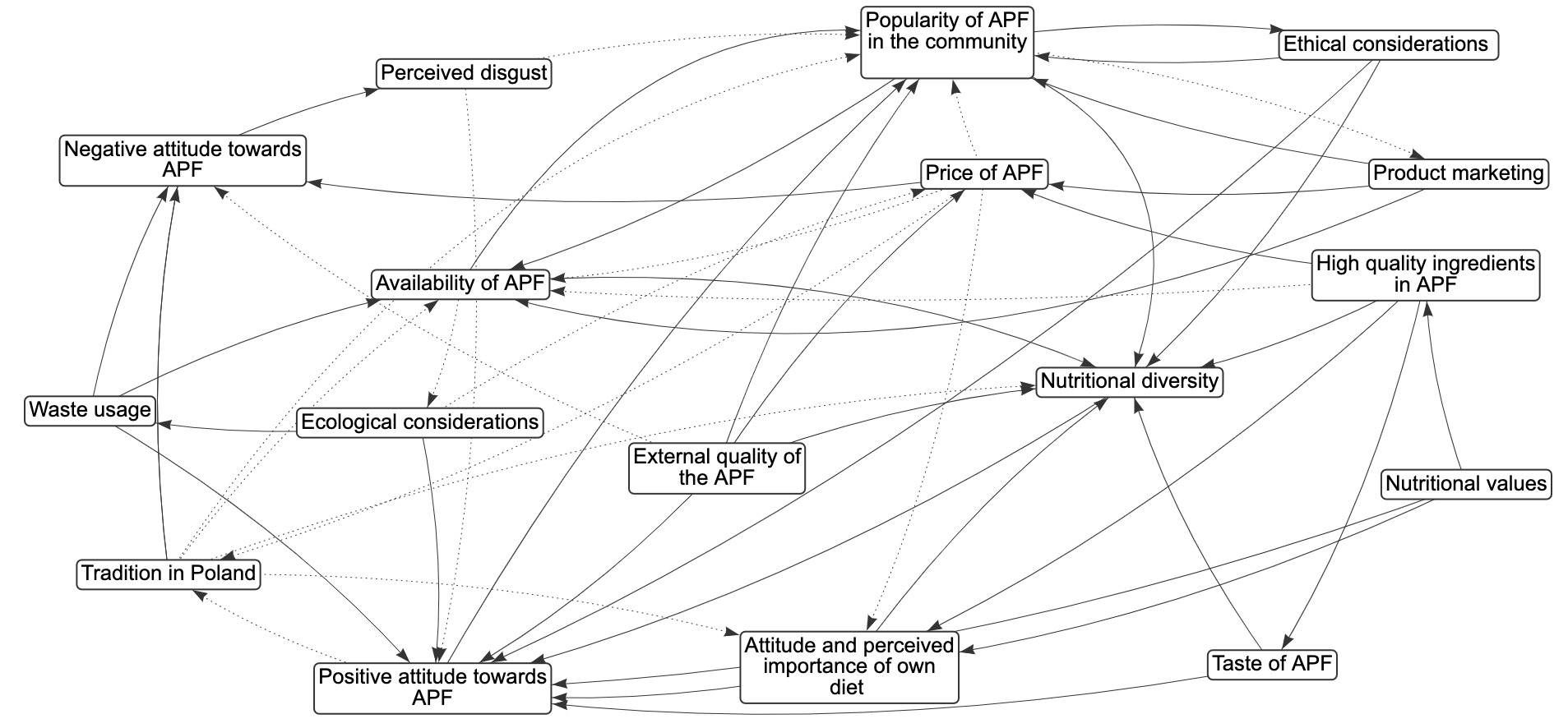


*Note.* Solid lines represent the positive edge; dotted lines represent the negative edge.

*Note.* Solid lines represent the positive edge; dotted lines represent the negative edge

**Figure S11**

*Map Developed During 1^st^ System Mapping Workshops with Nutrition Specialists in Poland*


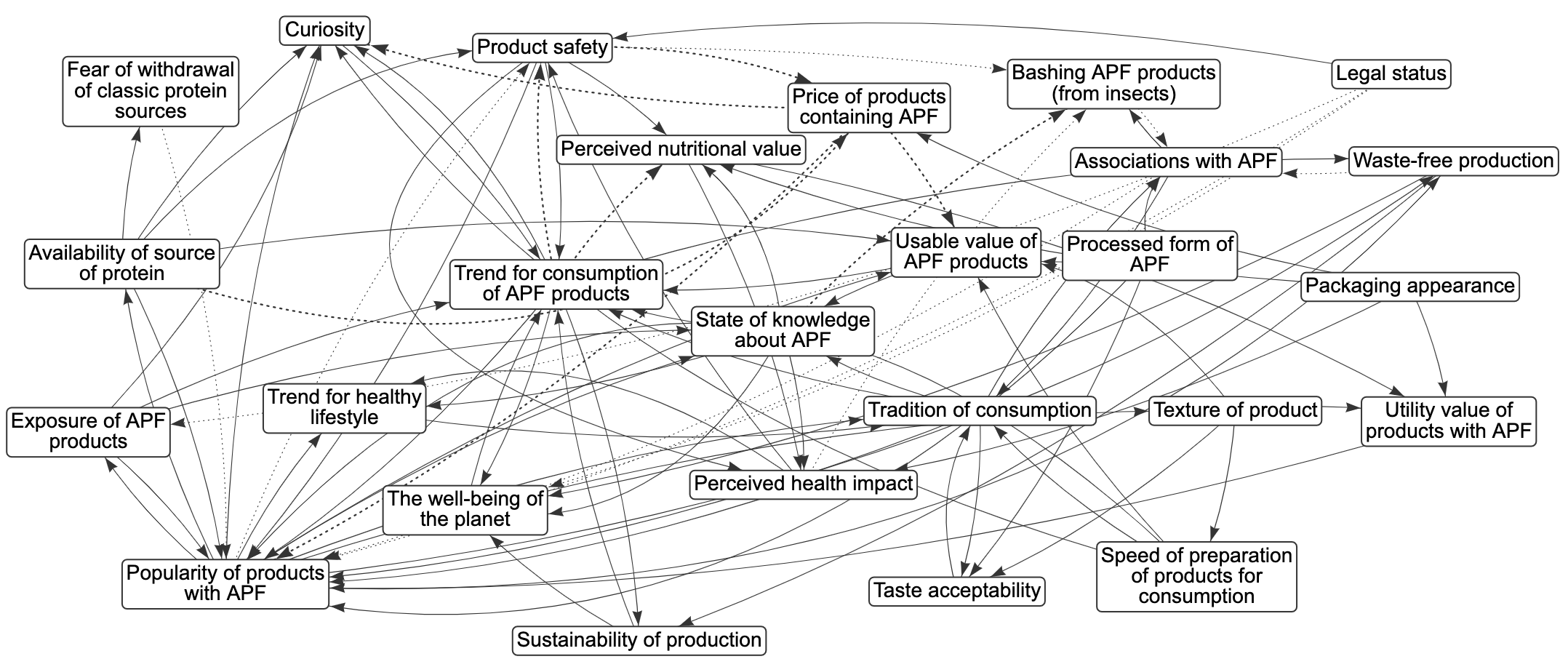


*Note.* Solid lines represent the positive edge; dotted lines represent the negative edge.

**Figure S12**

*Map Developed During 2^nd^ System Mapping Workshops with Nutrition Specialists in Poland*


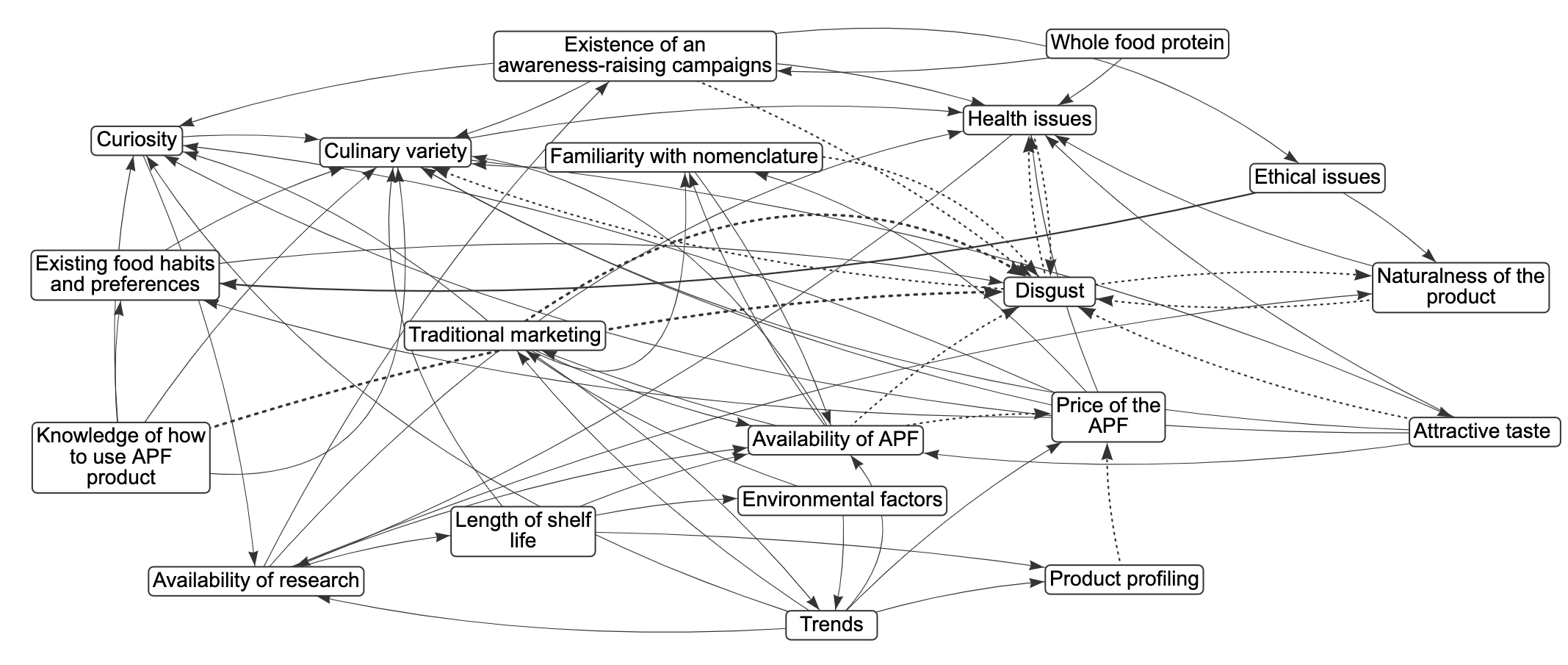


*Note.* Solid lines represent the positive edge; dotted lines represent the negative edge.

**Figure S13**

*Map Developed During 4^th^ System Mapping Workshops with Nutrition Specialists in Poland*

**Figure S13**

*Map Developed During 3^rd^ System Mapping Workshops with Nutrition Specialists in Poland*


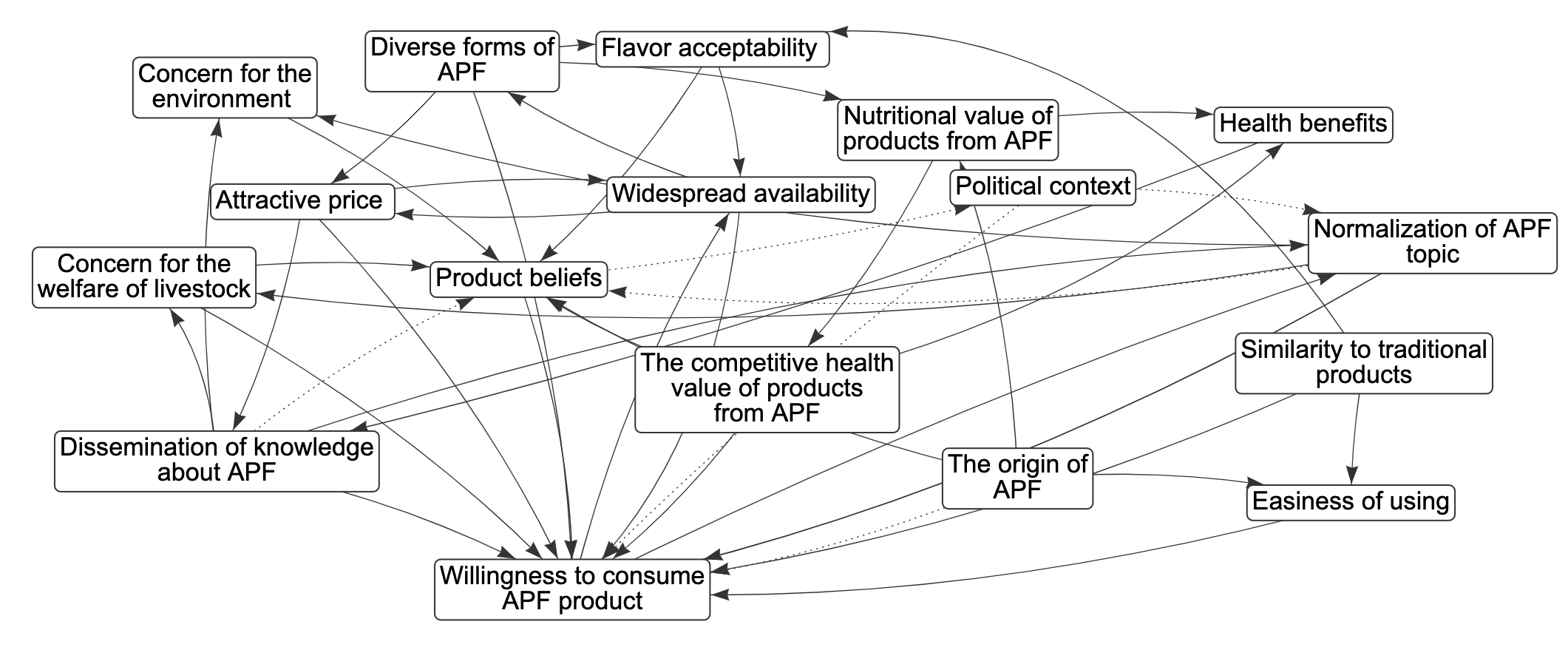


*Note.* Solid lines represent the positive edge; dotted lines represent the negative edge.

**Figure S14**

*Map Developed During System Mapping Workshops in Portugal*


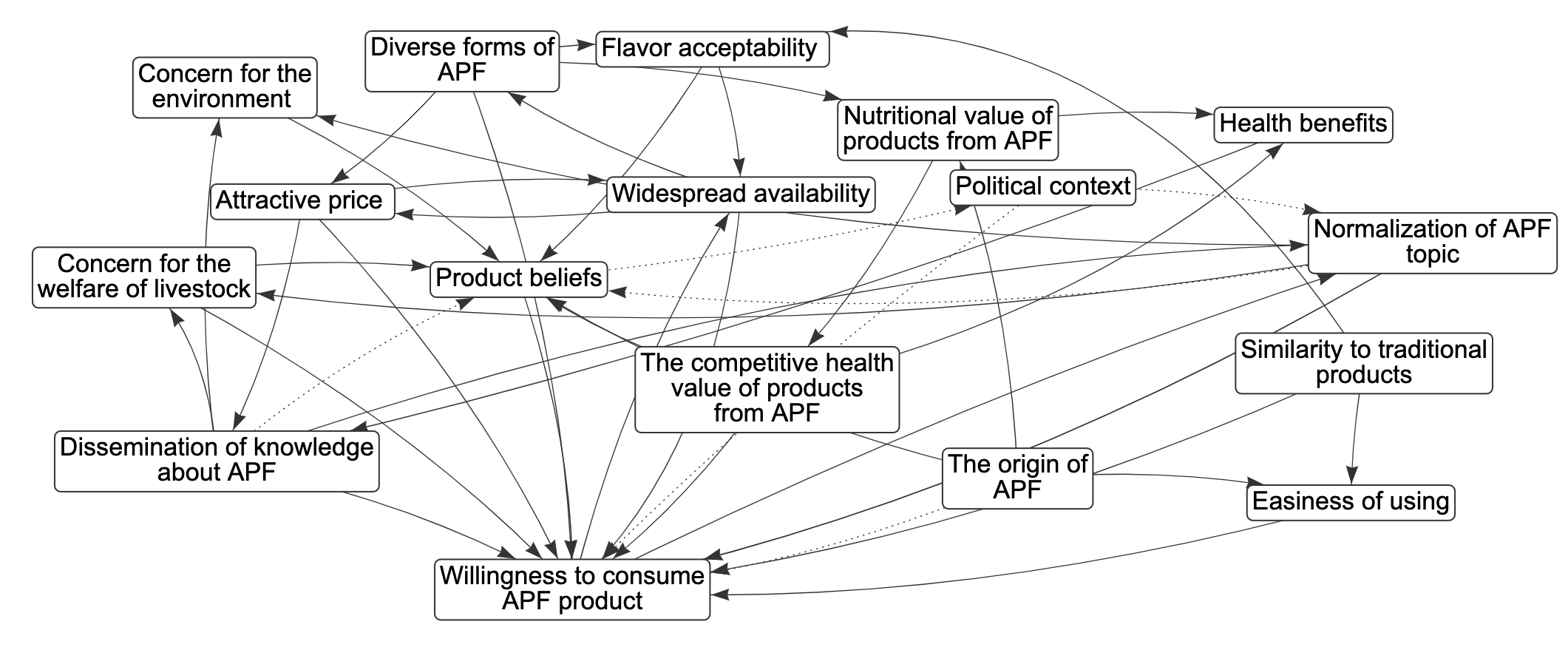


*Note.* Solid lines represent the positive edge; dotted lines represent the negative edge.

**Figure S15**

*Map Developed During System Mapping Workshops in Slovenia*


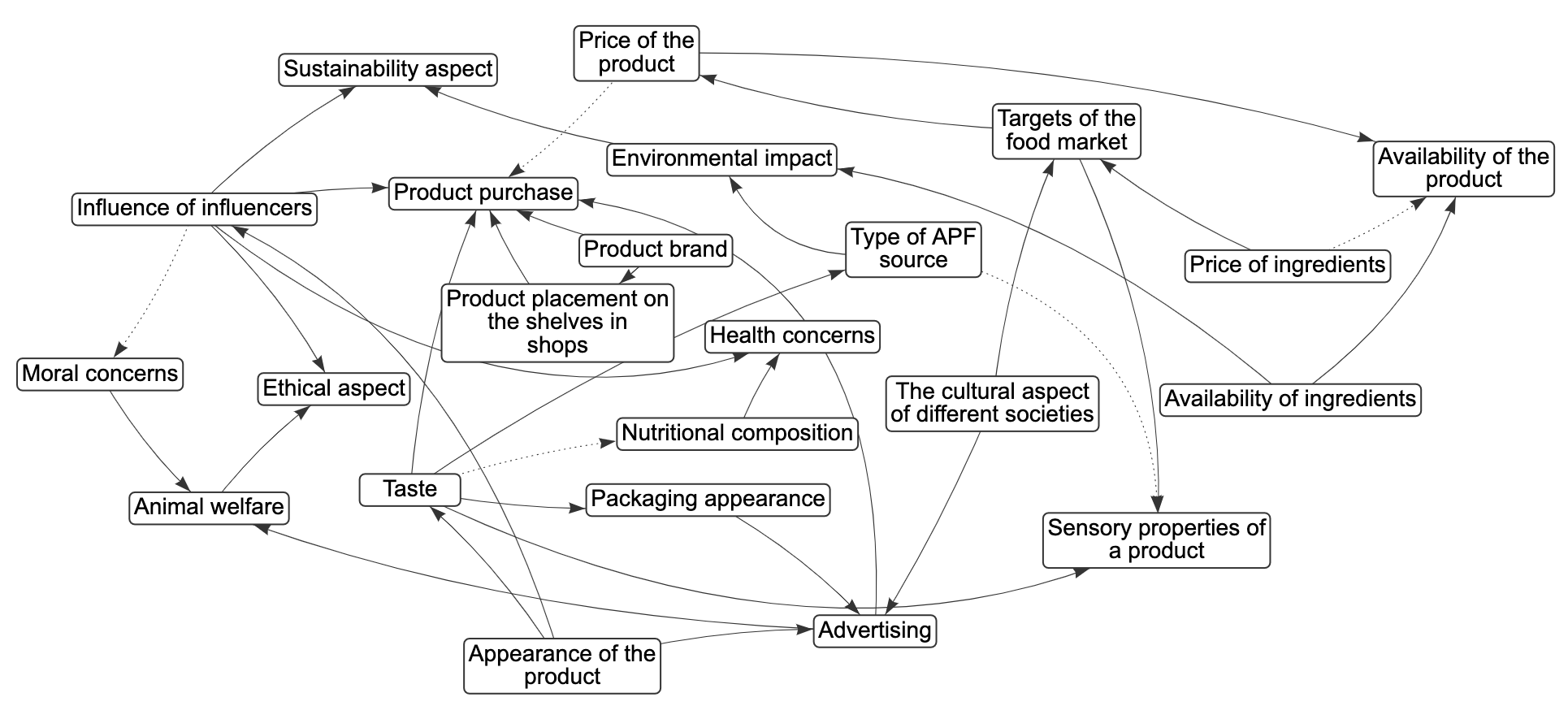


*Note.* Solid lines represent the positive edge; dotted lines represent the negative edge.

**Figure S16**

**Figure S16**

*Map Developed During System Mapping Workshops in Spain*


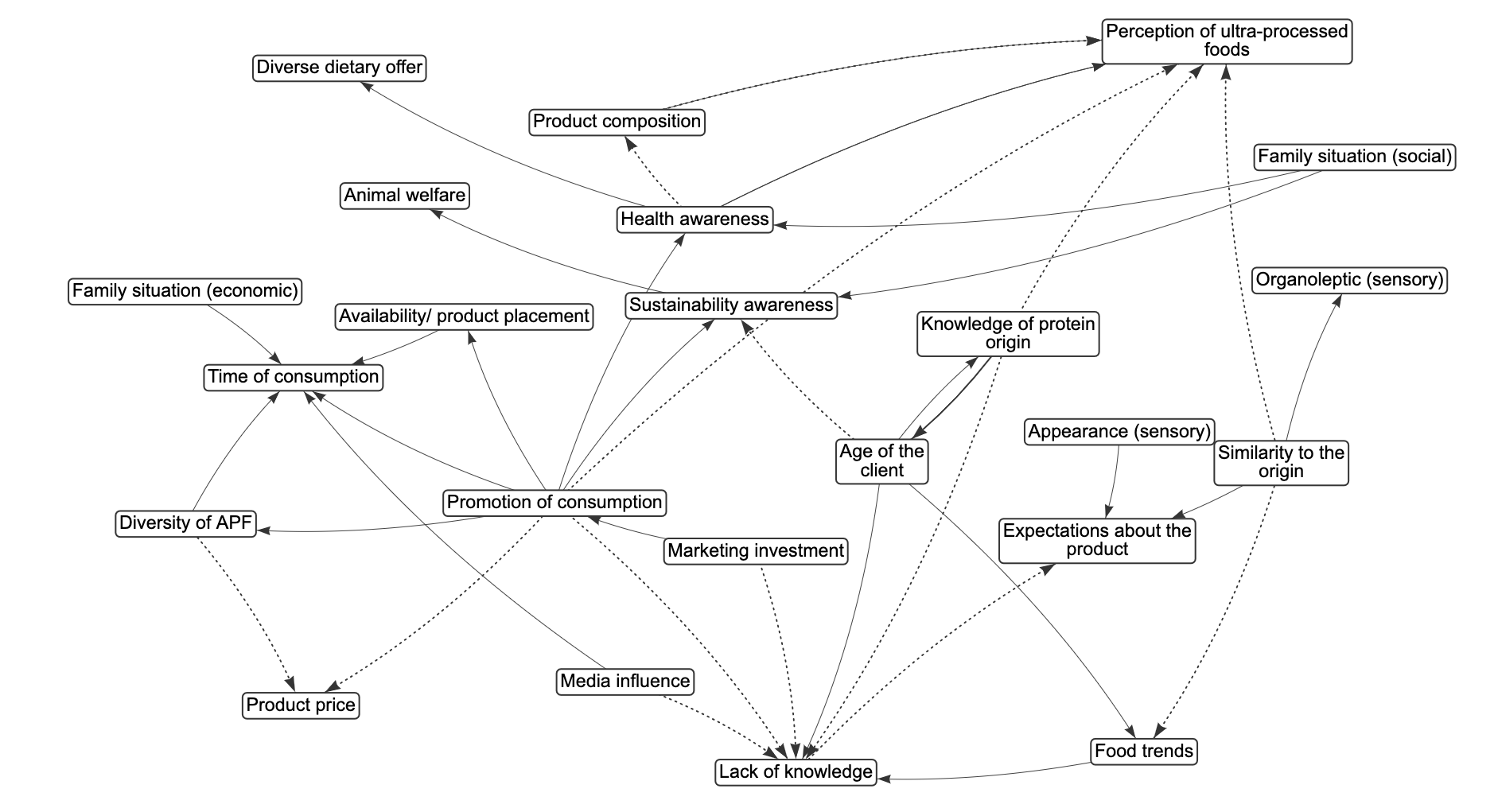


*Note.* Solid lines represent the positive edge; dotted lines represent the negative edge.

**Figure S17**

*Map Developed During System Mapping Workshops in Turkey*

*
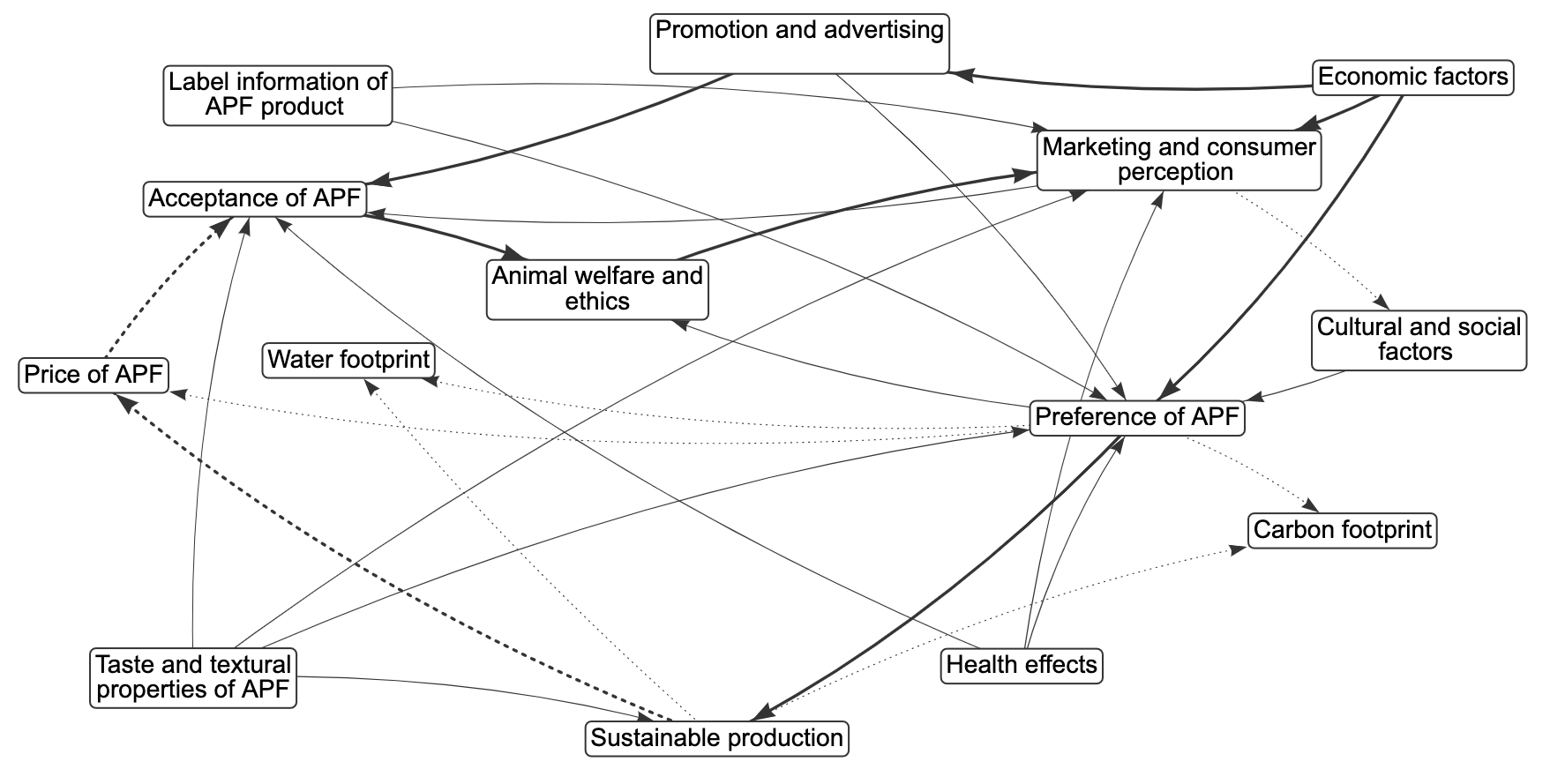
*

*Note.* Solid lines represent the positive edge; dotted lines represent the negative edge.

**Table S1**

*Definitions of Determinants Included in Each System Map*

| **Country** | **Name of the determinant in original language** | **Name of the determinant in English (translated by the local workshop moderators)** | **Definition of the determinant as provided by the stakeholders** |
| --- | --- | --- | --- |
| **Austria** |  |  |  |
|  | - | Regionality of APF production | Independence from international markets can be maintained due to regional production of APF products. |
|  | - | Health effects of APF products | Different metabolization of plant proteins may influence health status. |
|  | - | Ultra-processed foods | High level of procession in producing APF products. |
|  | - | Price | Price is a factor that may influence purchasing decisions. |
|  | - | Curiosity | Being curious about APF products. |
|  | - | Nutritional factor of plant proteins (PER) | Plant proteins are differently metabolized than animal proteins; PER = protein efficiency ratio. |
|  | - | Animal welfare and protection | The purchase of APF products helps protect animal welfare by minimalizing the purchase of animal products. |
|  | - | Sources of APF | Protein sources of APF products that do not fulfill sustainable criteria. |
|  | - | Protection of environment | Production of APF products that is lower in greenhouse gas emissions. |
|  | - | Ingredient overload in APF products | APF products contain lots of additional ingredients (e.g., preservatives, flavors, gelling agents) to ensure adequate product quality. |
|  | - | Availability of APF products | If APF products were more available, it might encourage people to buy them. |
|  | - | Olfactory perception of APF products | New olfactory factors (new taste, new smell, new mouth feel) affect the decision to buy certain product. |
| **Czech Republic** |  |  |  |
|  | - | Price | Price of APF products affects their acceptance by consumers. |
|  | - | Ethics | Plant-based products can be chosen more often than animal-based products because of their ethical aspects. |
|  | - | Health benefits | Acceptance of APF products can be higher because of their health benefits. |
|  | - | Environment | Sustainable production of APF could be more feasible than sustainable animal protein production. |
|  | - | Promotion, advertisement | Promotion of APF may influence consumers’ choices regarding APF. |
|  | - | Age | Younger people are more aware of health benefits of APF products. |
|  | - | Nutrition | Higher nutritional benefits of a product. |
|  | - | Taste preference | Dependence of taste preferences, which vary from person to person. |
|  | - | Fear of the unknown | Fear of the unknown product makes it harder to try novel food products. |
|  | - | Trends | Trends in food market influence the purchase of food products. |
| **Denmark** |  |  |  |
|  | - | Danish food culture | Danish food culture is based on meat which can be a barrier in trying APF products. |
|  | - | Perceived taste | Consumers’ experience of taste may influence their choice of product. |
|  | - | Availability | If products are visible on supermarket shelves, consumers are more likely to buy them. |
|  | - | Climate impact | Production of APF products may result in lower CO2 emissions compared to animal-based food products. |
|  | - | Knowledge of preparation | Consumers’ knowledge of how APF products can be used while cooking. |
|  | - | Health benefits | If people are aware of health benefits of APF, they may be more encouraged to use them. |
|  | - | Price | The price that consumers must pay for a product. |
|  | - | ‘Normalization’ of new products | People may feel that they are compromising if the product is highlighted as ‘different’, e.g., CO2-neutral, vegan etc. USP (unique selling proposition) is the essence of what makes your product or service better than those of competitors. |
|  | - | Socially acceptable | - |
|  | - | Energy content | Knowledge of how many insects are equivalent to a portion of chicken, for example. |
|  | - | Family suitability | A challenge for every member of a family to accept and eat APF products. |
|  | - | Lifestyle | Some consumers actively choose APF based on climate considerations and a desire for self-sufficiency, for example. |
|  | - | Storytelling | Good storytelling of APF (small, committed, and dedicated local producers) product may catch the attention of a buyer. |
|  | - | Consistency/colour | - |
|  | - | Marketing | The fit of a marketing strategy for a specific target audience. |
|  | - | Product knowledge | Knowledge of the ingredients and nutritional composition of APF product. |
|  | - | Animal welfare | Buying APF products may reduce interest in buying meat products, and it will positively impact animal welfare. |
|  | - | Trend | When APF products are promoted in the media, they often gain visibility. |
|  | - | Shelf life | Easiness and length of storage compared to other food products. |
|  | - | Food safety | - |
|  | - | Perceived naturalness of products | The level of natural ingredients and composition of a product. |
|  | - | Security of supply | APF can be a viable source of food for a growing population. |
| **France** |  |  |  |
|  | - | Taste and savors | Level of tastiness of APF product. |
|  | - | Process transparency | Clear explanation of how products are made, step by step. |
|  | - | Cultural challenge | Knowledge of APF products within a specific culture. |
|  | - | Naming | Specific naming of a product. |
|  | - | Price | Level of accessibility of a product to a consumer. |
|  | - | Affordability (process) | Cost of the technology used in production of APF product. |
|  | - | Public policy strategy | Level of public policy engagement in promotion and education of APF products. |
|  | - | Use and habits | Establishment of certain food habits. |
|  | - | Education | Level of knowledge on how to cook APF products and what dishes can be prepared with them. |
|  | - | Sustainability | Environmental impact of APF products. |
|  | - | Animal welfare | - |
|  | - | Health and wellness | Nutritional benefits of APF products. |
|  | - | Naturality | Amount of natural ingredients in a product. |
|  | - | Product shape, size and texture | Appearance and packaging of APF products. |
|  | - | Trust and credibility | Level of trust in producer and APF products. |
|  | - | Prescriptors strategy | - |
|  | - | Participation of the consumer | Consumer participation in co-creation of a product |
|  | - | Context of consumption | Place where APF product is consumed. |
|  | - | Consumer and eater profiles | Taking into consideration specific consumer profiles, such as young or elderly people. |
|  | - | Representation/perception | Visual and perceptual representation of a product. |
| **Germany** |  |  |  |
|  | Ethik | Ethics | Presence of values important for a certain individual. |
|  | Umweltaspekte | Environmental aspects | Level of importance of an environmental aspect for an individual. |
|  | Gesundheit | Health | Interest in APF products due to their health benefits. |
|  | wahrgenomenne Gesundheit | Perceived health | Perceived beneficial APF’s influence on individuals’ health. |
|  | - | Convenience | Level of convenience in preparation of an APF product. |
|  | Preis | Price | Product price influences purchase decisions. |
|  | Geschmack | Taste | APF products’ taste. |
|  | Sensorik | Sensorial characteristics | Sensory perception of APF products. |
|  | Verfügbarkeit | Availability | Widespread presence of products in stores. |
|  | Mediale Diskussion | Discussion on media | Amount of news that individual receives from the media about APF products. |
|  | Branding/starke Marken | Branding/strong brands | Level of popularity and familiarity with a certain brand |
|  | Branchenimage der alternativen Proteine | Image of APF industry | How APF products are represented via producers and in marketing strategies. |
|  | Vertrautheit | Familiarity | To which extent an individual is familiar with APF products. |
|  | Gewohnheit | Habits | Presence of specific eating habits in an individual. |
|  | Traditionelle Ernaehrungsgewohnheiten | Traditional diet habits | Level of influence that culture has on eating habits of individuals. |
|  | Identitaet/Selbstwahrnehmung | Identity/Self-perception | Influence of a certain identity on eating choices. |
|  | Peer-Group-Einfluss | Peer-group-influence | Presence of peer-group influence regarding food choices. |
|  | Soziale Norm | Social norms | Level to which a behavior is approved by society. |
| **Greece** |  |  |  |
|  | - | Taste | How tasteful APF product is. |
|  | - | Texture | Texture of final APF product. |
|  | - | Cultural eating habits | Strength of individual’s cultural habits in choosing certain foods. |
|  | - | Price | Price of the final APF product. |
|  | - | Toxicity of APF | - |
|  | - | Ethical considerations | Level of ethical considerations regarding animal welfare and environmental protection. |
|  | - | Marketing | Visibility of promotion of products to overcome stereotypes. |
|  | - | Familiarization with APF | Level of how an individual is familiar with APF product in the context of cultural background. |
|  | - | Availability | Availability of APF products. |
|  | - | Age | Consumers age. |
|  | - | Sustainability of APF | Level of sustainability of the final APF product. |
|  | - | Safety legislation for APF | Safety of APF product. |
|  | - | Health impact | Impact of APF on consumers’ health. |
|  | - | Consumer awareness | How much consumers are aware of APF products, their sustainability and health effect. |
|  | - | Health-based eating habits | Presence of eating restrictions in individual that can limit their food choices |
|  | - | Educational level | How much an individual is educated on food choices. |
|  | - | Labelling | Presence of information about sustainability on the label of food product. |
| **Italy** |  |  |  |
|  | - | Healthy choices | Tendency to buy food products that have positive health-related impact. |
|  | - | Socio-economic impact | Positive impact of APF production on social and economic factors (e.g., new jobs possibilities). |
|  | - | Environmental impact | Product’s impact on the environment. |
|  | - | Protein intake | How much an APF product can replace animal-based protein. |
|  | - | Curiosity | Level of consumer’s curiosity and willingness to buy APF products. |
|  | - | Visibility of APF products | Visibility of APF products in advertisements and on supermarket shelves. |
|  | - | Availability of APF delivery | Availability of APF in food delivery apps. |
|  | - | Advertising | Good advertisement and valuable marketing strategy of APF products. |
|  | - | Animal welfare | Possibility to replace animal-based food with APF for ethical reasons. |
|  | - | Food safety | Safety of APF products. |
|  | - | Nutritional benefits | The number of nutritional benefits of APF products. |
|  | - | Price | How expensive APF products are. |
|  | - | Food neophobia | Consumers’ perception of disgust towards new food products. |
|  | - | Food taste | A good food taste, texture and smell of APF product. |
| **Norway** |  |  |  |
|  | - | Packaging of APF | Material for packaging of APF product. |
|  | - | APF store placement | - |
|  | - | Novelty of different APF | - |
|  | - | Gradual adaption towards APF for humans | - |
|  | - | Regulations of products with APF | - |
|  | - | Availability of products with APF | - |
|  | - | Amino acid profile in APF | - |
|  | - | Protein substitution for a traditional diet | - |
|  | - | Hybrid products of traditional and APF | - |
|  | - | Marketing of APF | - |
|  | - | Overview of food recipes for APF | Presence of tutorials on how to prepare meals with APF products and available recipes. |
|  | - | Price of traditional proteins | - |
|  | - | Promotion of APF | - |
|  | - | Professionalism in the production of APF | - |
|  | - | Industrial scale production of APF | - |
|  | - | Degree of processing of APF | - |
|  | - | Locally oriented (Norwegian) production of APF | - |
|  | - | Food safety related to APF | - |
|  | - | Climate, nature and environmental effects from APF | - |
|  | - | Association to origin of APF | Associations that people have with certain APF products, e.g., insects. |
|  | - | Good sensory properties of APF | Both taste, smell and texture. |
|  | - | Price of APF | - |
|  | - | Perceived healthiness of APF | - |
|  | - | Convenience to use in a regular diet | - |
|  | - | Diet for Norwegians not oriented around APF | - |
|  | - | Animal welfare in production of traditional proteins | - |
|  | - | Health consequences for the consumers | - |
|  | - | Mainstream marketing of APF | - |
|  | - | Availability in food-serving outlets | The availability of APF in cafeteria, institutions, food service and out of home – sectors. |
|  | - | Alternative marketing of APF | If marketing of APF products is tailored for specific groups e.g., sports, elderly people. |
|  | - | Online shopping for APF | - |
|  | - | Regulatory development of APF | Regular updates of APF products on the market. |
|  | - | Consumer perception of APF | - |
|  | - | Shelf life of products with APF | - |
| **Poland** |  |  |  |
|  | Zdrowy skład produktu | Healthy APF product composition | Refers to ingredients included in a product that promote health. |
|  | Przejrzysta informacja o składzie | Transparent ingredient information | Ingredient information is clearly visible on the packaging. |
|  | Świadomość dot. zastępstwa | Substitution awareness | Refers to the general public’s awareness of the possibility of replacing animal protein with other forms of protein. |
|  | Świadomość dot. dobra zwierząt | Animal welfare awareness | Public awareness of the overuse of animals for meat production. |
|  | Świadomość dot. ekologii | Environmental awareness | People’s knowledge of the impact of animal meat production on environment (e.g., gas emissions) compared to plant-based alternatives. |
|  | Jasne oznaczenie produktu dot. ekologii/ dobra zwierząt | Clear product labeling regarding health/ecology/animal welfare | Transparent labels on products, providing essential information about their impact on health, environment, and animal rights; guiding consumers towards informed decisions by highlighting healthier or eco-friendly alternatives. |
|  | Znane/ tradycyjne produkty (dla konsumentów) zawierające APF | Familiar and well-known consumer brands containing APF | Inclusion of APF sources in products by established and recognized brands. |
|  | Edukacja wczesna dot. APF | Early education on APF | Integration of knowledge about APF sources into early educational programs (targeting both children and their parents). |
|  | Normalizacja konsumpcji APF | Normalization of APF consumption | Normalization of APF consumption by making APF products widely available. |
|  | Cena produktów APF | Pricing of APF products | If pricing of APF products is equalized to well-known products in the market, such as meat. |
|  | Atrakcyjna oprawa graficzna opakowań | Attractive packaging | Attractive, appealing, desirable APF products packaging. |
|  | Dostępność w sklepach/ marketach | Availability in stores/markets | Availability of products in both large and small stores in urban and rural areas. |
|  | Otwartość na próbowanie APF | Openness to trying APF | Curiosity and openness about trying new APF products. |
|  | Zakres preferencji smakowych | Range of taste preferences | Range of individuals taste preferences; a narrow range of taste preferences makes it more challenging to choose APF as consumers may be less willing to try new or unfamiliar flavors. Conversely, a broad range of taste preference facilitates the selection of APF. |
|  | Różnorodność produktów z APF | Variety of products with APF | A greater variety of APF product options. |
|  | Bezpieczne warunki produkcji APF | Safe production conditions of APF | Assurance of health and safety standards in the manufacturing process of APF (need for strict rules and clear methods in producing APF to make sure people can trust that these foods are safe and of good quality). |
|  | Promocja APF przez autorytety/ influencerów | Promotion of APF by entities/influencers | Involvement of well-known entities/influencers in the field of nutrition advocating for the choice of APF. |
|  | Wiek | Age | Age of consumer. |
|  | Starzejące się społeczeństwo | Aging society | Older people may be more hesitant toward APF products. |
|  | Promocja tradycyjna | Traditional promotion | Refers to promotion of APF products through conventional advertising channels such as print media (newspapers, magazines) and broadcast media (TV, radio). |
|  | Technologia produkcji APF | APF production technology | Modern, widespread, and advanced methods used in production of alternative proteins. |
|  | Edukacja (ustawiczna) | Education (continuous) | Ongoing educational efforts to inform and engage individuals about APF at every stage of development and age. |
|  | Konkurencja między producentami | Competition among APF producers | APF producers competing against each other; competition makes producers work harder to improve what they sell, making these foods more appealing and affordable for everyone. |
|  | Aprobata społeczna | Social approval | Belief that people have about behavior of others, and the desire to conform to their actions. The more a product is consumed, the more socially acceptable it becomes. |
|  | Względy etyczne | Ethical considerations | Consideration of animal welfare. |
|  | Negatywna postawa wobec APF | Negative attitude towards APF | Negative attitudes and beliefs. Personal attitude towards eating specific products containing APF, e.g., eating insects may trigger negative attitudes. |
|  | Wykorzystanie odpadków | Waste usage | Reusing products few times in a row. |
|  | Wysokość ceny APF | Price of APF | Price of APF product. |
|  | Wartości odżywcze | Nutritional values | Good quality ingredients in APF product. |
|  | Jakość wewnętrzna APF | High quality ingredients in APF | No pesticides or preservatives, accurate way of preserving product. |
|  | Dostępność APF | Availability of APF | High availability of APF products. |
|  | Popularność APF w społeczeństwie | Popularity of APF in the community | Commonness of consuming APF products among people. |
|  | Urozmaicenie żywienia | Nutritional diversity | Diet rich in diverse sources of proteins. |
|  | Smak APF | Taste of APF | Taste preferences of individual. |
|  | Stosunek do własnej diety | Attitude and perceived importance of own diet | Caring about one’s diet, keeping a healthy diet to maintain good health. |
|  | Tradycja w Polsce | Tradition in Poland | Strong religious, cultural and social attitudes about eating. |
|  | Względy ekologiczne | Ecological considerations | The extent to which producers care about the environment during production process. |
|  | Odczuwany wstręt | Perceived disgust | Characteristic of a product that discourage to eat APF. |
|  | Jakość zewnętrzna APF | External quality of the APF | Aesthetic of a product. |
|  | Marketing produktu | Product marketing | Promotion of APF product. |
|  | Pozytywna postawa wobec APF | Positive attitude towards APF | Positive attitude and beliefs, individual attitude towards eating APF products, e.g., not eating meat is more ethical. |
|  | Tradycja spożycia | Tradition of consumption | Tradition of consuming products in the family, region, and country (Poland). |
|  | Dostępność źródła białka | Availability of source of protein | Availability of products or raw materials related to APF. |
|  | Cena produktów zawierających APF | Price of products containing APF | Price of APF products comparable to classic protein sources. |
|  | Akceptowalność smaków | Taste acceptability | An individual characteristic of the taste of the finished product containing APF. |
|  | Dobro planety | The well-being of the planet | Care for the environment. |
|  | Stan wiedzy na temat APF | State of knowledge about APF | Consumer’s knowledge about APF. |
|  | Szybkość przygotowania produktów do spożycia | Speed of preparation of products for consumption | Ease of self-preparation of dishes from products containing APF at home. |
|  | Postrzegana wartość odżywcza | Perceived nutritional value | Consumers’ perception of nutritional value of a given APF product compared to a classic protein source. |
|  | Skojarzenia z APF | Associations with APF | Perceptions about APF; for example, insects can be associated with pests which can make it difficult to accept APF as a product for consumption when one feels revulsion. |
|  | Popularność produktów z APF | Popularity of products with APF | Awareness that one’s consumption of products with APF influences and encourages others to buy APF products. |
|  | Forma APF | Processed form of APF | Processed products/unprocessed sources of APF. |
|  | Zrównoważona produkcja | Sustainability of production | Positive and safe impact on the environment. |
|  | Bezpieczeństwo produktu | Product safety | Information on whether or not APF products contain pesticides, etc. |
|  | Postrzegany wpływ na zdrowie | Perceived health impact | How a person perceives the product in terms of safety for their own health. |
|  | Wartość użytkowa produktów APF | Usable value of APF products | What consumers can make from an APF product; for example, a certain dish for dinner or sweet baked goods. |
|  | Moda na zdrowy styl życia | Trend for healthy lifestyle | Associating APF products with healthy lifestyle. |
|  | Moda na spożycie produktów z APF | Trend for consumption of APF products | Influence of known figures on the desire to buy APF products which is not associated with nutritional values. |
|  | Wygląd opakowania | Packaging appearance | Attractiveness of packaging and visible product composition on the package. |
|  | Konsystencja produktu | Texture of product | Preference for a specific texture of APF. |
|  | Pomysł na użycie w diecie produktów z APF | Utility value of products with APF | Knowledge of variety of recipes in which APF products can be used. |
|  | Obawa przed stopniowym wycofywaniem klasycznych źródeł białka | Fear of withdrawal from classic protein sources | Concern that when alternative products will be popular, they will exclude products that are familiar (products with classic protein sources). |
|  | Ciekawość | Curiosity | Desire to try new/other products that contain APF. |
|  | Hejt na produkty z APF (z insektów) | Bashing APF products (made from insects) | Negative public perceptions/opinions/ hate- comments in social media, dealing with the consumption of APF products from, for example, insects. |
|  | Ekspozycja produktów z APF przez autorytety | Exposure of APF products | Exposure of APF products by prominent, popular people on TV or social media. A form of trend and continuous exposure of consumers to see these products on TV or social media. |
|  | Status prawny | Legal status | Legal application/implementation of new foods which affects the supply and popularity of products and positively affects their safety and legality. |
|  | Produkcja bezodpadowa | Waste-free production | Producing protein with no or limited waste which affects sustainable production. |
|  | Dotychczasowe nawyki i preferencje żywieniowe | Existing food habits and preferences | Strength of one’s habits and food preferences. |
|  | Atrakcyjny smak | Attractive taste | Tasty, encouraging flavor of APF product. |
|  | Cena APF | Price of the APF | Price of APF products. |
|  | Istnienie kampanii uświadamiających | Existence of awareness-raising campaigns | Emergence of APF campaigns which can make potential consumers more aware of its existence. |
|  | Kwestie zdrowotne | Health issues | Existence of health recommendations aimed to reduce animal protein in diets. |
|  | Trend | Trends | Promotion of APF by influencers/known figures on social media. |
|  | Wiedza jak zastosować produkt | Knowledge of how to use APF product | Knowledge of how to prepare and use products in recipes. Ready recipes containing APF. |
|  | Dostępność produktów z APF | Availability of APF | Presence of APF products in local stores. |
|  | Urozmaicenie kulinarne | Culinary variety | Readiness to diversify dishes by adding APF products. |
|  | Kwestie etyczne | Ethical issues | Existence of a campaign to make people aware of the suffering of animals used in animal protein or dairy production. |
|  | Pełnowartościowe białko | Whole food protein | One serving of an APF product contains approximately the same amount of protein as meat. |
|  | Dostępność badań | Availability of research | Availability of studies that prove safety of APF products. |
|  | Czynniki środowiskowe | Environmental factors | Information on the use of water, carbon footprint and energy in the production of APF vs. traditional protein sources. |
|  | Wstręt | Disgust | Aversion towards APF products. |
|  | Marketing tradycyjny | Traditional marketing | Presenting and promoting APF through radio and TV to reach various audiences. |
|  | Oswojenie z nazewnictwem | Familiarity with nomenclature | Encouraging, familiar names of APF products. |
|  | Profilowanie produktów | Product profiling | Matching APF products to the affluence of consumers. |
|  | Ciekawość | Curiosity | Interest in trying new products. |
|  | Długość czasu przechowywania | Length of shelf life | Expiration date of a product; how long a product can be stored at home. |
|  | Naturalność produktu | Naturalness of the product | Non-processed products, no pesticides or preservatives. |
|  | Pochodzenie APF | The origin of APF | Origin of certain protein sources. |
|  | Kontekst polityczny | Political context | Influence of politics on protein selection approach. |
|  | Powszechna dostępność | Widespread availability | Ease of purchasing APF products in markets/stores. |
|  | Troska o środowisko | Concern for the environment | Level of one’s concern for the environment. |
|  | Chęć spożycia produktu z APF | Willingness to consume APF product | Desire/wish to consume substantial amounts of protein from alternative sources without disgust. |
|  | Przekonania dot. produktów | Product beliefs | Attitudes/beliefs/frequent aversion to APF products based on knowledge. |
|  | Akceptowalność smaków | Flavor acceptability | Attractiveness of taste of APF for the consumer compared to traditional products. |
|  | Wartość odżywcza produktów z APF | Nutritional value of products from APF | Good nutritional composition of a product, high amount of protein and no preservatives. |
|  | Podobieństwo do tradycyjnych produktów | Similarity to traditional products | Similarity of APF products to well-known products. |
|  | Atrakcyjna cena | Attractive price | Similarity of price of products from APF to traditional products. |
|  | Łatwość zastosowania | Easiness of using | Convenience of using or adding products from APF to meals. |
|  | Upowszechnienie wiedzy o APF | Dissemination of knowledge about APF | Amount of knowledge about APF. |
|  | Troska o dobro zwierząt hodowlanych | Concern for the welfare of livestock | Concern for the welfare of animals destined for meat. |
|  | Normalizacja tematu APF | Normalization of APF topic | Normalization of APF topic in media, TV and campaigns. |
|  | Konkurencyjna wartość zdrowotna produktów z APF | The competitive health value of products from APF | Greater nutritional value of APF products than of original/traditional products. |
|  | Korzyści zdrowotne | Health benefits | Public perception of health/environmental values of APF products. |
|  | Różnorodna forma APF | Diverse forms of APF | Variety of forms of products with APF (e.g., burgers, gyros, sausages, etc.) increasingchances of finding a suitable product. |
| **Portugal** |  |  |  |
|  | - | Socio-economic factors | Promotion of social and economic sustainability. |
|  | - | Environmental sustainability | Level of environmental impact of APF products. |
|  | - | Organoleptic characteristics | Smell, taste and texture of APF products. |
|  | - | Disgust | Disgust for APF products. |
|  | - | Local employment development | Locality of APF products. |
|  | - | Cultural aspects | Strength of cultural influence on eating habits. |
|  | - | Ethical issues in production | Presence of unethical activities that are undertaken during production process. |
|  | - | High price of APF | Expensive price of APF products. |
|  | - | Product availability | Availability of APF products. |
|  | - | Concern for animal welfare | Consumers’ care for animal welfare. |
|  | - | Food security | When all people, at all times, have physical and economic access to sufficient safe and nutritious food that meets their dietary needs and food preferences for an active and healthy life. |
|  | - | Trends | Higher exposure of APF in society, media etc. |
|  | - | Food neophobia | Aversion to new food. |
|  | - | Nutritional profile of APF | Nutritional profile of APF products. |
|  | - | Health impact | Positive or negative impact of APF on health. |
|  | - | Trust | Consumer trust in APF products. |
|  | - | Ultra-processed foods | Perception of APF as unnatural and highly processed product. |
|  | - | Illiteracy about APF | Lack of knowledge and information about APF. |
|  | - | Nutrition and health claims | Nutrition and health claims made on labels of APF products. |
|  | - | Perceived positive impact on health | Consumer’s perceived positive impact on health. |
|  | - | Perceived negative impact on health | Consumer’s perceived negative impact on health. |
| **Slovenia** |  |  |  |
|  | Cenovna dostopnost končnega produkta | Price of the product | How much an APF product costs. |
|  | Razpoložljivost končnega izdelka | Availability of the product | Availability of plant-based proteins in supermarkets, smaller stores. |
|  | Ciljno tržišče | Targets of the food market | Specific goals of the market that are related to sustainability. |
|  | Cenovna dostopnost surovin | Price of ingredients | Price level of ingredients used in APF products. |
|  | Razpoložljivost surovin | Availability of ingredients | Availability of ingredients used in APF products in a country. |
|  | Vpliv na okolje | Environmental impact | Product’s impact on the environment. |
|  | Trajnostni vidik (trajnostne študije) | Sustainability aspect | Importance of lowering carbon footprint. |
|  | Vpliv vplivnežev | Influence of influencers | Level of influence that an influencer or a specific company has on an individual. |
|  | Blagovna znamka izdelka | Product brand | Consumer’s trust and acceptance of a certain brand. |
|  | Pozicioniranje izd. na policah | Product placement on the shelves in shops | Planning product placement in a way that can encourage consumers to buy APF products. |
|  | Moralni pomisleki | Moral concerns | Consumer’s level of acceptance for APF. |
|  | Etični vidik | Ethical aspect | Negative feelings towards animal proteins. |
|  | Dobrobit živali | Animal welfare | Plant-based protein products are considered to cause less harm to animals. |
|  | Oglaševanje | Advertising | Correct advertisements of products. |
|  | Videz izdelka | Appearance of the product | Visual aspects of a product; if it is appealing and attractive for a consumer. |
|  | Okus | Taste | Taste of APF products. |
|  | Videz embalaže izdelka | Packaging appearance | Visibility and attractiveness of packaging of APF products. |
|  | Hranilna sestava | Nutritional composition | Fiber content, protein value, energy value, and (saturated) fat composition in APF products. |
|  | Vpliv beljakovin na zdravje | Health concerns | Health benefits of APF products. |
|  | Senzorične lastnosti izdelkov | Sensory properties of a product | How much an APF product is sensory appealing. |
|  | Kulturni vidik različnih družb | The cultural aspect of different societies | Level of cultural influence. |
|  | Vrsta alternativnega vira beljakovin | Type of APF source | Acceptance of certain plant-based proteins, such as soy, is much more negative compared to other plant-based proteins. |
| **Spain** |  |  |  |
|  | Oferta dieta variada | Diverse dietary offer | APF products can provide some nutrients that traditional products do not. |
|  | Factor novedad | Food trends | Innovative products attract attention and many people consume them out of curiosity. |
|  | Percepcion de ultraprocesado | Perception of ultra-processed foods | New products made with alternative proteins may create the perception in the consumer that they are ultra-processed products because several ingredients must be included to achieve the final product. |
|  | Composición | Product composition | Composition of a product understood as the quantity of ingredients and additives it contains. |
|  | Conocimiento de procedencia de fuente proteica | Knowledge of protein origin | Awareness of APF products. |
|  | Desconocimiento | Lack of knowledge | Lack of awareness of the existence of APF and their potential health or environmental benefits. |
|  | Visibilidad mediatica | Media influence | Visibility of APF products in communication channels, social networks and television advertisements mainly. |
|  | Inversión marketing | Marketing investment | Investment in advertising by a company to advertise new products. For example, a company with traditional products launches a new line of novel foods but does not advertise them. |
|  | Organoleptico (sensorial) | Organoleptic (sensory) | Organoleptic characteristics of the product: texture, palatability, taste, flavor. |
|  | Expectativas del producto | Expectations about the product | Consumer’s initial expectations or judgement of this type of product. |
|  | Precio del producto | Product price | Final price of the product. |
|  | Situación familiar (social) | Family situation (social) | Structure of the family unit and its components. Whether the food provider is aware of these new proteins or not, or whether the food provider has to take care of children or only himself/herself. |
|  | Situación familiar (economica) | Family situation (economic) | Family financial situation. |
|  | Edad de comprador | Age of the client | Age of food providers or household members. |
|  | Diversidad de oferta | Diversity of APF | Diversity of new APF products, not only meat analogues. |
|  | Promocion de consumo | Promotion of consumption | Promotion of consumption of this kind of products by institutions. |
|  | Disponibilidad/Inclusión | Availability/product placement | Availability of this kind of products in supermarkets and included with the rest of the traditional products. |
|  | Concienciación sostenibilidad | Sustainability awareness | Consumer awareness of potential sustainability benefits. |
|  | Aspecto (sensorial) | Appearance (sensory) | First sight perception of the product and its presentation (packaging). Visual impact, attractiveness. |
|  | Concienciación salud | Health awareness | Consumer awareness of potential health benefits of APF products. |
|  | Momento de consumo | Time of consumption | Consumption opportunities of the products (time of consumption) and their inclusion in the traditional diet or cuisine of the country. |
|  | Similtud al origen | Similarity to the origin | Similarity to the product it replaces or imitates by including the APF in an existing matrix or creating a new matrix of product. |
|  | Bienestar animal | Animal welfare | Reduction of consumption of traditional animal products. |
| **Turkey** |  |  |  |
|  | - | Acceptance of APF | Acceptance of APF products as substitutes for animal-based products. |
|  | - | Animal welfare and ethics | Inadequate production conditions that are harmful to animals. |
|  | - | Marketing and consumer perception | Marketing of APF products and their image in the media. |
|  | - | Economic factors | Costs of animal-based products production. |
|  | - | Preference of APF | Plant-based products can be preferred for their health effects, environmental conditions and ethical issues more than animal-based products. |
|  | - | Cultural and social factors | Strong cultural and social tradition of eating. |
|  | - | Carbon footprint | Plant-based production results in a lower carbon footprint than animal production. |
|  | - | Health effects | Positive health effects of eating APF products. |
|  | - | Sustainable production | Effectiveness of sustainable production of APF products. |
|  | - | Taste and textural properties of APF | Taste and texture of the APF products. |
|  | - | Price of APF | Price of APF products. |
|  | - | Water footprint | Plant-based production results in a lower water footprint than animal production. |
|  | - | Label information of APF product | Explanatory and transparent label information of APF products affects consumer’s preferences. |
|  | - | Promotion and advertising | Stronger promotion and advertising of APF products. |

**Table S2**

*The Leverage Points Identified in Network Analysis in Respective Countries*

| **Country** | **Degree** | **Betweenness centrality** | **Closeness centrality** | **Eigenvector centrality** |
| --- | --- | --- | --- | --- |
| **Austria** | Curiosity = 4  Health effects of APF products = 4  Ingredient overload in APF products = 4 | Curiosity = 34  Availability of APF products = 30.5  Animal welfare and protection = 10.5 | Availability of APF products = 0.52  Curiosity = 0.52  Animal welfare and protection = 0.42  Health effects of APF products = 0.42  Ingredient overload in APF products = 0.42  Price = 0.42 | Ingredient overload in APF products = 0.49  Health effects of APF products = 0.49  Nutritional factor of plant proteins (PER) = 0.41  Ultra-processed foods = 0.41 |
| **Czech Republic** | Fear of the unknown = 5  Price = 4 | Fear of the unknown = 28  Price = 21  Health benefits = 8 | Fear of the unknown = 0.64  Price = 0.56  Health benefits = 0.45 | Fear of the unknown = 0.58  Price = 0.49  Promotion = 0.45 |
| **Denmark** | Family suitability = 4 | Family suitability = 53  Food safety = 36  Knowledge of preparation = 35 | Family suitability = 0.47  Knowledge of preparation = 0.43  Food safety = 0.39  Availability = 0.39 | Family suitability = 0.49  Danish food culture = 0.38  Knowledge of preparation = 0.38 |
| **France** | Use and habits = 10  Education = 7  Representation / perception = 5 | Use and habits = 88.8  Naturality = 50.7  Process transparency = 38 | Use and habits = 0.59  Education = 0.53  Representation / perception = 0.53 | Use and habits = 0.51  Education = 0.45  Representation / perception = 0.35 |
| **Germany** | Familiarity = 7  Availability = 8  Social norms = 8 | Familiarity = 3.2  Social norms = 7.6  Availability = 5.6 | Familiarity = 0.60  Social norms = 0.64  Availability = 0.62 | Familiarity = 0.32  Availability = 0.36  Social norms = 0.36 |
| **Greece** | Familiarization with APF = 11  Educational level = 7  Sustainability APF = 6  Price = 6  Consumer awareness = 6 | Familiarization with APF = 35.4  Sustainability APF = 21.2  Educational level = 20.9 | Familiarization with APF = 0.67  Educational level = 0.62  Sustainability APF = 0.59 | Familiarization with APF = 0.47  Educational level = 0.37  Age = 0.26 |
| **Italy** | Advertising = 8  Curiosity = 7  Environmental impact = 6 | Advertising = 12.7  Curiosity = 12  Food safety = 4.9 | Advertising = 0.72  Curiosity = 0.68  Environmental impact = 0.62 | Advertising = 0.43  Curiosity = 0.38  Environmental impact = 0.32  Protein intake = 0.32 |
| **Norway** | Regulations of products with APF = 6  Marketing of APF = 6  Regulatory development for APF = 5  Shelf life of products with APF = 5 | Regulations of products with APF = 101.5  Marketing of APF = 86.6  Degree of processing of APF = 66.8 | Regulations of products with APF = 0.42  Degree of processing of APF = 0.40  Marketing of APF = 0.39  Nature and environmental effects from APF = 0.39 | Regulations of products with APF = 0.28  Nature and environmental effects from APF = 0.27  Degree of processing of APF = 0.26 |
| **Poland (1st workshop)** | Popularity of APF in the society = 12  Positive attitude towards APF = 11  Price of APF = 9  Availability of APF = 9 | Positive attitude towards APF = 27.5  Popularity of APF in the society = 14.2  Price of APF = 11.4 | Positive attitude towards APF = 0.76  Price of APF = 0.70  Popularity of APF in the society = 0.70  Nutritional diversity = 0.70 | Nutritional diversity = 0.35  Popularity of APF in the society = 0.35  Positive attitude towards APF = 0.35 |
| **Poland (2nd workshop)** | Social approval = 4  Normalization of APF consumption = 4  Education (continuous) = 4 | Openness to trying APF = 100.3  Pricing of APF products = 91.8  Promotion of APF by entities/influencers = 72.8 | Openness to trying APF = 0.39  Promotion of APF by entities/influencers = 0.37  Pricing of APF products = 0.35  Education (continuous) = 0.35 | Education (continuous) = 0.44  Normalization of APF consumption = 0.37  Social approval = 0.37 |
| **Poland (3rd workshop)** | Popularity of products with APF = 24  Trend for consumption of APF products = 15  Product safety = 11 | Popularity of products with APF = 120  Trend for consumption of APF products = 37.4  Tradition of consumption = 20 | Popularity of products with APF = 0.81  Trend for consumption of APF products = 0.64  State of knowledge about APF = 0.63 | Popularity of products with APF = 0.47  Trend for consumption of APF products = 0.32  State of knowledge about APF = 0.30 |
| **Poland (4th workshop)** | Culinary variety = 13  Disgust = 12  Availability of APF = 11 | Culinary variety = 19.4  Disgust = 19.1  Health issues = 16.4 | Culinary variety = 0.70  Disgust = 0.66  Curiosity = 0.63  Price of the APF = 0.63 | Culinary variety = 0.37  Disgust = 0.33  Availability of APF = 0.32 |
| **Poland (5th workshop)** | Willingness to consume APF product = 15  Product beliefs = 9  Normalization of APF topic = 8 | Willingness to consume APF product = 46.2  Product beliefs = 18.4  Dissemination of knowledge about APF = 11.6 | Product beliefs = 0.70  Dissemination of knowledge about APF = 0.64  Normalization of APF topic = 0.59 | Willingness to consume APF product = 0.47  Dissemination of knowledge about APF = 0.33  Normalization of APF topic = 0.32 |
| **Portugal** | Nutritional profile of APF = 5  Trends = 5  Trust = 5 | Trends = 73.4  Trust = 60.9  Ethical issues in production = 52.8 | Trends = 0.44  Trust = 0.42  Illiteracy about APF = 0.41 | Trust = 0.44  Trends = 0.40  Illiteracy about APF = 0.36 |
| **Slovenia** | Influence of influencers = 6  Product purchase = 6  Taste = 6 | Product purchase = 80.3  Influence of influencers = 61.6  Taste = 51.3 | Product purchase = 0.54  Taste = 0.48  Influence of influencers = 0.47 | Product purchase = 0.42  Taste = 0.39  Influence of influencers = 0.33 |
| **Spain** | Promotion of consumption = 9  Perception of ultra-processed foods = 7  Lack of knowledge= 7 | Promotion of consumption = 108.7  Lack of knowledge = 56.9  Perception of ultra-processed foods = 48 | Promotion of consumption = 0.60  Perception of ultra-processed foods = 0.51  Lack of knowledge = 0.51 | Promotion of consumption = 0.50  Lack of knowledge = 0.37  Perception of ultra-processed foods = 0.28 |
| **Turkey** | Preference of APF = 11  Marketing and consumer perception = 7  Sustainable production = 5 | Preference of APF = 39  Marketing and consumer perception = 9.5  Sustainable production = 3.7 | Preference of APF = 0.87  Sustainable production = 0.62  Marketing and consumer perception = 0.62 | Preference of APF = 0.49  Marketing and consumer perception = 0.35  Taste = 0.30 |

**Table S3**

*Feedback Loops Developed in System Mapping Workshops in Austria*

| **No.** | **Feedback loops description** |
| --- | --- |
| 1 | Curiosity **(-) -->** Reduction of ingredient overload in APF products **(+) ->** Ultra-processed foods **(-) -->** Nutritional factor of plant proteins (PER) **(+) ->** Health effect of APF products **(+) ->** Curiosity |
| 2 | Curiosity **(-) -->** Ingredient overload in APF products **(+) -->** Ultra-processed foods **(-) -->** Health effect of APF products **(+) ->** Curiosity |
| 3 | Curiosity **(-) -->** Ingredient overload in APF products **(-) -->** Nutritional factor of plant proteins (PER) **(+) ->** Health effect of APF products **(+) ->** Curiosity |
| 4 | Curiosity **(-) -->** Ingredient overload in APF products **(-) -->** Health effect of APF products **(+) ->** Curiosity |
| 5 | Protection of environment **(-) -->** Sources of APF **(+) ->** Animal welfare and protection **(+) ->** Protection of environment |

*Note.* APF – alternative protein food; (+)-> a positive edge, (-)-> a negative edge.

**Table S4**

*Feedback Loops Developed in System Mapping Workshops in Germany*

| **No.** | **Feedback loops description** |
| --- | --- |
| 1 | Availability **(+) ->** Social norms **(+) ->** Familiarity **(+) ->** Habits **(+) ->** Taste **(+) ->** Acceptance of APF **(+) ->** Availability |
| 2 | Availability **(+) ->** Social norms **(+) ->** Peer-group-influence **(+) ->** Identity/Self-perception **(+) ->** Environmental aspects **(+) ->** Acceptance of APF **(+) ->** Availability |
| 3 | Availability **(+) ->** Social norms **(+) ->** Identity/Self-perception **(+) ->** Environmental aspects **(+) ->** Acceptance of APF **(+) ->** Availability |
| 4 | Availability **(+) ->** Social norms **(+) ->** Familiarity **(+) ->** Habits **(+) ->** Acceptance of APF **(+) ->** Availability |
| 5 | Availability **(+) ->** Social norms **(+) ->** Familiarity **(+) ->** Image of APF industry **(+) ->** Acceptance of APF **(+) ->** Availability |
| 6 | Availability **(+) ->** Social norms **(+) ->** Familiarity **(+) ->** Taste **(+) ->** Acceptance of APF **(+) ->** Availability |
| 7 | Availability **(+) ->** Social norms **(+) ->** Peer-group-influence **(+) ->** Identity/Self-perception **(+) ->** Acceptance of APF **(+) ->** Availability |
| 8 | Availability **(+) ->** Social norms **(+) ->** Peer-group-influence **(+) ->** Identity/Self-perception **(+) ->** Environmental aspects **(-) -->** Availability |
| 9 | Availability **(+) ->** Habits **(+) ->** Familiarity **(+) ->** Image of APF industry **(+) ->** Acceptance of APF **(+) ->** Availability |
| 10 | Availability **(+) ->** Habits **(+) ->** Familiarity **(+) ->** Taste **(+) ->** Acceptance of APF **(+) ->** Availability |
| 11 | Availability **(+) ->** Familiarity **(+) ->** Habits **(+) ->** Taste **(+) ->** Acceptance of APF **(+) ->** Availability |
| 12 | Availability **(+) ->** Social norms **(+) ->** Identity/Self-perception **(+) ->** Environmental aspects **(-) -->** Availability |
| 13 | Availability **(+) ->** Social norms **(+) ->** Identity/Self-perception **(+) ->** Acceptance of APF **(+) ->** Availability |
| 14 | Availability **(+) ->** Social norms **(+) ->** Familiarity **(+) ->** Acceptance of APF **(+) ->** Availability |
| 15 | Availability **(+) ->** Social norms **(+) ->** Peer-group-influence **(+) ->** Acceptance of APF **(+) ->** Availability |
| 16 | Availability **(+) ->** Habits **(+) ->** Familiarity **(+) ->** Acceptance of APF **(+) ->** Availability |
| 17 | Availability **(+) ->** Habits **(+) ->** Taste **(+) ->** Acceptance of APF **(+) ->** Availability |
| 18 | Availability **(+) ->** Familiarity **(+) ->** Habits **(+) ->** Acceptance of APF **(+) ->** Availability |
| 19 | Availability **(+) ->** Familiarity **(+) ->** Image of APF industry **(+) ->** Acceptance of APF **(+) ->** Availability |
| 20 | Availability **(+) ->** Familiarity **(+) ->** Taste **(+) ->** Acceptance of APF **(+) ->** Availability |
| 21 | Availability **(-) -->** Price **(+) ->** Sensory characteristics **(+) ->** Acceptance of APF **(+) ->** Availability |
| 22 | Availability **(-) -->** Price **(+) ->** Taste **(+) ->** Acceptance of APF **(+) ->** Availability |
| 23 | Availability **(+) ->** Social norms **(+) ->** Acceptance of APF **(+) ->** Availability |
| 24 | Availability **(+) ->** Habits **(+) ->** Acceptance of APF **(+) ->** Availability |
| 25 | Availability **(+) ->** Familiarity **(+) ->** Acceptance of APF **(+) ->** Availability |
| 26 | Availability **(-) -->** Price **(-) -->** Acceptance of APF **(+) ->** Availability |

*Note.* APF – alternative protein food; (+)-> a positive edge, (-)-> a negative edge.

**Table S5**

*Feedback Loops Developed in System Mapping Workshops in Greece*

| **No.** | **Feedback loops description** |
| --- | --- |
| 1 | Availability **(+) ->** Marketing **(+) ->** Consumer awareness **(+) ->** Health-based eating habits **(+) ->** Familiarization with APF **(+) ->** Availability |
| 2 | Availability **(+) ->** Marketing **(+) ->** Consumer awareness **(+) ->** Familiarization with APF **(+) ->** Availability |
| 3 | Availability **(+) ->** Marketing **(+) ->** Familiarization with APF **(+) ->** Availability |
| 4 | Availability **(-) -->** Price **(-) -->** Familiarization with APF **(+) ->** Availability |

*Note.* APF – alternative protein food; (+)-> a positive edge, (-)-> a negative edge.

**Table S6**

*Feedback Loops Developed in System Mapping Workshops in Italy*

| **No.** | **Feedback loops description** |
| --- | --- |
| 1 | Healthy choices **(-) -->** Environmental impact **(-) -->** Socio-economic impact **(+) ->** Price **(+) ->** Food safety **(+) ->** Advertising **(+) -> V**isibility of APF products **(+) ->** Curiosity **(+) ->** Healthy choices |
| 2 | Healthy choices **(-) -->** Environmental impact **(-) -->** Socio-economic impact **(+) ->** Price **(+) ->** Food safety **(+) ->** Advertising **(+) -> V**isibility of APF products **(+) ->** Healthy choices |
| 3 | Healthy choices **(-) -->** Environmental impact **(-) -->** Socio-economic impact **(+) ->** Price **(+) ->** Food safety **(+) ->** Advertising **(+) ->** Curiosity **(+) ->** Healthy choices |
| 4 | Environmental impact **(-) -->** Socio-economic impact **(+) ->** Price **(+) ->** Food safety **(+) ->** Advertising **(+) ->** Visibility of APF products (+) -> Curiosity (-) --> Environmental impact |
| 5 | Healthy choices **(-) -->** Environmental impact **(-) -->** Socio-economic impact **(+) ->** Price **(+) ->** Food safety **(+) ->** Advertising **(+) ->** Healthy choices |
| 6 | Environmental impact **(-) -->** Socio-economic impact **(+) ->** Price **(+) ->** Food safety **(+) ->** Advertising **(+) ->** Curiosity **(-) -->** Environmental impact |
| 7 | Healthy choices **(-) -->** Environmental impact **(-) -->** Advertising **(+) -> V**isibility of APF products **(+) ->** Curiosity **(+) ->** Healthy choices |
| 8 | Healthy choices **(-) -->** Environmental impact **(-) -->** Socio-economic impact **(+) ->** Price **(-) -->** Availability of APF delivery **(+) ->** Healthy choices |
| 9 | Healthy choices **(-) -->** Environmental impact **(-) -->** Advertising **(+) ->** Availability of APF delivery **(+) ->** Healthy choices |
| 10 | Healthy choices **(-) -->** Environmental impact **(-) -->** Advertising **(+) ->** Curiosity **(+) ->** Healthy choices |
| 11 | Environmental impact **(-) -->** Advertising **(+) -> V**isibility of APF products **(+) ->** Curiosity **(-) -->** Environmental impact |
| 12 | Healthy choices **(-) -->** Environmental impact **(-) -->** Advertising **(+) ->** Healthy choices |
| 13 | Environmental impact **(-) -->** Advertising **(+) ->** Curiosity **(-) -->** Environmental impact |
| 14 | Healthy choices **(-) -->** Environmental impact **(-) -->** Socio-economic impact **(+) ->** Healthy choices |

*Note.* APF – alternative proteins; (+)-> a positive edge, (-)-> a negative edge.

**Table S7**

*Feedback Loops Developed in System Mapping Workshops in Norway*

| **No.** | **Feedback loops description** |
| --- | --- |
| 1 | Professionalism in the production of APF **(+) ->** Regulatory development of APF **(+) ->** Professionalism in the production of APF |

*Note.* APF – alternative proteins; (+)-> a positive edge, (-)-> a negative edge.

**Table S8**

*Feedback Loops Developed in 1^st^ System Mapping Workshops with Adolescents in Poland*

| **No.** | **Feedback loops description** |
| --- | --- |
| 1 | Social approval **(+) ->** Normalization of APF consumption **(+) ->** Social approval |

*Note.* APF – alternative protein food; (+)-> a positive edge, (-)-> a negative edge.

**Table S9**

*Feedback Loops Developed in 2^nd^ System Mapping Workshops with Adolescents in Poland*

| **No.** | **Feedback loops description** |
| --- | --- |
| 1 | Popularity of APF in the community **(-) -->** Product marketing **(+) ->** Availability of APF **(-) -->** Ecological considerations **(-) -->** Price of APF **(-) -->** Tradition in Poland **(-) -->** Popularity of APF in the community |
| 2 | Popularity of APF in the community **(-) -->** Product marketing **(+) ->** Availability of APF **(-) -->** Ecological considerations **(-) -->** Price of APF **(-) -->** Popularity of APF in the community |
| 3 | Popularity of APF in the community **(-) -->** Product marketing **(+) ->** Price of APF **(-) -->** Tradition in Poland **(-) -->** Availability of APF **(+) ->** Popularity of APF in the community |
| 4 | Popularity of APF in the community **(+) ->** Availability of APF **(-) -->** Ecological considerations **(-) -->** Price of APF **(-) -->** Tradition in Poland **(-) -->** Popularity of APF in the community |
| 5 | Popularity of APF in the community **(-) -->** Product marketing **(+) ->** Price of APF **(-) -->** Availability of APF **(+) ->** Popularity of APF in the community |
| 6 | Popularity of APF in the community **(+) ->** Availability of APF **(-) -->** Ecological considerations (perceiving the product as less ecological) **(-) -->** Price of APF (prices become higher due to low sales) **(-) -->** Popularity of APF in the community |
| 7 | Popularity of APF in the community **(-) -->** Product marketing **(+) ->** Price of APF **(-) -->** Tradition in Poland (people feel threatened that their eating culture is threatened/replaced by APF) **(-) -->** Popularity of APF in the community |
| 8 | Availability of APF **(-) -->** Ecological considerations **(-) -->** Price of APF **(-) -->** Tradition in Poland **(-) -->** Availability of APF |
| 9 | Popularity of APF in the community **(-) -->** Product marketing **(+) ->** Availability of APF **(+) ->** Popularity of APF in the community |
| 10 | Popularity of APF in the community **(-) -->** Product marketing **(+) ->** Price of APF **(-) -->** Popularity of APF in the community |
| 11 | Price of APF **(-) -->** Availability of APF **(-) -->** Ecological considerations **(-) -->** Price of APF |
| 12 | Availability of APF **(-) -->** Ecological considerations **(+) ->** Waste usage **(+) ->** Availability of APF |

*Note.* APF – alternative protein food; (+)-> a positive edge, (-)-> a negative edge.

**Table S10**

*Feedback Loops Developed in 1^st^ System Mapping Workshops with Nutrition Specialists in Poland*

| **No.** | **Feedback loops description** |
| --- | --- |
| 1 | Popularity of products with APF **(+) ->** Exposure of APF products **(+) ->** State of knowledge about APF **(+) ->** Perceived health impact **(+) ->** Product safety **(+) ->** Trend for consumption of APF products **(+) ->** Popularity of products with APF |
| 2 | Popularity of products with APF **(+) ->** Exposure of APF products **(+) ->** State of knowledge about APF **(+) ->** Trend for consumption of APF products **(-) -->** Product safety **(+) ->** Perceived health impact **(+) ->** Popularity of products with APF |
| 3 | Popularity of products with APF **(+) ->** Tradition of consumption **(+) ->** State of knowledge about APF **(+) ->** Perceived health impact **(+) ->** Product safety **(+) ->** Trend for consumption of APF products **(+) ->** Popularity of products with APF |
| 4 | Popularity of products with APF **(+) ->** Tradition of consumption **(+) ->** State of knowledge about APF **(+) ->** Trend for consumption of APF products **(-) -->** Product safety **(+) ->** Perceived health impact **(+) ->** Popularity of products with APF |
| 5 | Popularity of products with APF **(+) ->** State of knowledge about APF **(+) ->** Perceived health impact **(+) ->** Product safety **(+) ->** Trend for consumption of APF products **(+) ->** Popularity of products with APF |
| 6 | Popularity of products with APF **(+) ->** State of knowledge about APF **(+) ->** Trend for consumption of APF products **(-) -->** Product safety **(+) ->** Perceived health impact **(+) ->** Popularity of products with APF |
| 7 | Popularity of products with APF **(+) ->** Exposure of APF products **(+) ->** State of knowledge about APF **(+) ->** Perceived health impact **(+) ->** Product safety **(+) ->** Popularity of products with APF |
| 8 | Popularity of products with APF **(+) ->** Exposure of APF products **(+) ->** State of knowledge about APF **(+) ->** Trend for consumption of APF products **(-) -->** Product safety **(+) ->** Popularity of products with APF |
| 9 | Popularity of products with APF **(+) ->** Exposure of APF products **(+) ->** Trend for consumption of APF products **(-) -->** Product safety **(+) ->** Perceived health impact **(+) ->** Popularity of products with APF |
| 10 | Popularity of products with APF **(+) ->** Tradition of consumption **(+) ->** State of knowledge about APF **(+) ->** Perceived health impact **(+) ->** Product safety **(+) ->** Popularity of products with APF |
| 11 | Popularity of products with APF **(+) ->** Tradition of consumption **(+) ->** State of knowledge about APF **(+) ->** Trend for consumption of APF products **(-) -->** Product safety **(+) ->** Popularity of products with APF |
| 12 | Popularity of products with APF **(+) ->** Tradition of consumption **(+) ->** Trend for consumption of APF products **(-) -->** Product safety **(+) ->** Perceived health impact **(+) ->** Popularity of products with APF |
| 13 | Popularity of products with APF **(+) ->** State of knowledge about APF **(+) ->** Perceived health impact **(+) ->** Product safety **(+) ->** Popularity of products with AP |
| 14 | Popularity of products with APF **(+) ->** State of knowledge about APF **(+) ->** Trend for consumption of APF products **(-) -->** Product safety **(+) ->** Popularity of products with APF |
| 15 | Popularity of products with APF **(+) ->** Exposure of APF products **(+) ->** State of knowledge about APF **(+) ->** Perceived health impact **(+) ->** Popularity of products with APF |
| 16 | Popularity of products with APF **(+) ->** Exposure of APF products **(+) ->** State of knowledge about APF **(+) ->** Trend for consumption of APF products **(+) ->** Popularity of products with APF |
| 17 | Popularity of products with APF **(+) ->** Exposure of APF products **(+) ->** Trend for consumption of APF products **(-) -->** Product safety **(+) ->** Popularity of products with APF |
| 18 | Popularity of products with APF **(+) ->** Availability of the source of protein **(+) ->** Product safety **(+) ->** Trend for consumption of APF products **(+) ->** Popularity of products with APF |
| 19 | Popularity of products with APF **(+) ->** Availability of the source of protein **(+) ->** Product safety **(+) ->** Perceived health impact **(+) ->** Popularity of products with APF |
| 20 | Popularity of products with APF **(+) ->** Tradition of consumption **(+) ->** State of knowledge about APF **(+) ->** Perceived health impact **(+) ->** Popularity of products with APF |
| 21 | Popularity of products with APF **(+) ->** Tradition of consumption **(+) ->** State of knowledge about APF **(+) ->** Trend for consumption of APF products **(+) ->** Popularity of products with APF |
| 22 | Popularity of products with APF **(+) ->** Tradition of consumption **(+) ->** Trend for consumption of APF products **(-) -->** Product safety **(+) ->** Popularity of products with APF |
| 23 | Popularity of products with APF **(+) ->** State of knowledge about APF **(+) ->** Perceived health impact **(+) ->** Popularity of products with APF |
| 24 | Popularity of products with APF **(+) ->** State of knowledge about APF **(+) ->** Trend for consumption of APF products **(+) ->** Popularity of products with APF |
| 25 | Popularity of products with APF **(+) ->** Exposure of APF products **(+) ->** Trend for consumption of APF products **(+) ->** Popularity of products with APF |
| 26 | Popularity of products with APF **(+) ->** Availability of the source of protein **(+) ->** Product safety **(+) ->** Popularity of products with APF |
| 27 | Popularity of products with APF **(-) -->** Product safety **(+) ->** Trend for consumption of APF products **(+) ->** Popularity of products with APF |
| 28 | Popularity of products with APF **(-) -->** Product safety **(+) ->** Perceived health impact **(+) ->** Popularity of products with APF |
| 29 | Popularity of products with APF **(+) ->** Tradition of consumption **(+) ->** Trend for consumption of APF products **(+) ->** Popularity of products with APF |

*Note.* APF – alternative protein food; (+)-> a positive edge, (-)-> a negative edge.

**Table S11**

*Feedback Loops Developed in 2^nd^ System Mapping Workshops with Nutrition Specialists in Poland*

| **No.** | **Feedback loops description** |
| --- | --- |
| 1 | Availability of research **(+) ->** Length of shelf life **(+) ->** Availability of APF **(+) ->** Traditional marketing **(+) ->** Trends **(+) ->** Price of the APF **(+) ->** Curiosity **(+) ->** Availability of research |
| 2 | Availability of research **(+) ->** Length of shelf life **(+) ->** Availability of APF **(+) ->** Traditional marketing **(+) ->** Trends **(+) ->** Curiosity **(+) ->** Availability of research |
| 3 | Availability of research **(+) ->** Traditional marketing **(+) ->** Trends **(+) ->** Availability of APF **(-) -->** Price of the APF **(+) ->** Curiosity **(+) ->** Availability of research |
| 4 | Availability of research **(+) ->** Availability of APF **(+) ->** Traditional marketing **(+) ->** Trends **(+) ->** Price of the APF **(+) ->** Curiosity **(+) ->** Availability of research |
| 5 | Availability of research **(+) ->** Availability of APF **(+) ->** Traditional marketing **(+) ->** Trends **(+) ->** Curiosity **(+) ->** Availability of research |
| 6 | Availability of research **(+) ->** Length of shelf life **(+) ->** Availability of APF **(+) ->** Traditional marketing **(+) ->** Curiosity **(+) ->** Availability of research |
| 7 | Availability of research **(+) ->** Length of shelf life **(+) ->** Availability of APF **(+) ->** Traditional marketing **(+) ->** Trends **(+) ->** Availability of research |
| 8 | Availability of research **(+) ->** Length of shelf life **(+) ->** Availability of APF **(-) -->** Price of the APF **(+) ->** Curiosity **(+) ->** Availability of research |
| 9 | Availability of research **(+) ->** Traditional marketing **(+) ->** Trends **(+) ->** Price of the APF **(+) ->** Curiosity **(+) ->** Availability of research |
| 10 | Availability of research **(+) ->** Traditional marketing **(+) ->** Availability of APF **(-) -->** Price of the APF **(+) ->** Curiosity **(+) ->** Availability of research |
| 11 | Availability of research **(+) ->** Traditional marketing **(+) ->** Trends **(+) ->** Curiosity **(+) ->** Availability of research |
| 12 | Availability of research **(+) ->** Availability of APF **(+) ->** Traditional marketing **(+) ->** Curiosity **(+) ->** Availability of research |
| 13 | Availability of research **(+) ->** Availability of APF **(+) ->** Traditional marketing **(+) ->** Trends **(+) ->** Availability of research |
| 14 | Availability of research **(+) ->** Availability of APF **(-) -->** Price of the APF **(+) ->** Curiosity **(+) ->** Availability of research |
| 15 | Availability of research **(+) ->** Traditional marketing **(+) ->** Curiosity **(+) ->** Availability of research |
| 16 | Availability of research **(+) ->** Traditional marketing **(+) ->** Trends **(+) ->** Availability of research |
| 17 | Availability of research **(+) ->** Existence of the awareness-raising campaigns **(+) ->** Curiosity **(+) ->** Availability of research |
| 18 | Traditional marketing **(+) ->** Trends **(+) ->** Availability of APF **(+) ->** Traditional marketing |

*Note.* APF – alternative protein food; (+)-> a positive edge, (-)-> a negative edge.

**Table S12**

*Feedback Loops Developed in 3^rd^ System Mapping Workshops with Nutrition Specialists in Poland*

| **No.** | **Feedback loops description** |
| --- | --- |
| 1 | Widespread product availability **(+) ->** Diverse forms of APF **(+) ->** Attractive price **(+) ->** Dissemination of knowledge about APF **(+) ->** Normalization of APF topic **(+) ->** Willingness to consume APF product **(+) ->** Widespread product availability |
| 2 | Widespread product availability **(+) ->** Attractive price **(+) ->** Dissemination of knowledge about APF **(+) ->** Normalization of APF topic **(+) ->** Willingness to consume APF product **(+) ->** Widespread product availability |
| 3 | Widespread product availability **(+) ->** Diverse forms of APF **(+) ->** Attractive price **(+) ->** Dissemination of knowledge about APF **(+) ->** Willingness to consume APF product **(+) ->** Widespread product availability |
| 4 | Widespread product availability **(+) ->** Attractive price **(+) ->** Dissemination of knowledge about APF **(+) ->** Willingness to consume APF product **(+) ->** Widespread product availability |
| 5 | Widespread product availability **(+) ->** Diverse forms of APF **(+) ->** Attractive price **(+) ->** Willingness to consume APF product **(+) ->** Widespread product availability |
| 6 | Widespread product availability **(+) ->** Diverse forms of APF **(+) ->** Flavor acceptability **(+) ->** Widespread product availability |
| 7 | Widespread product availability **(+) ->** Diverse forms of APF **(+) ->** Willingness to consume APF product **(+) ->** Widespread product availability |
| 8 | Widespread product availability **(+) ->** Diverse forms of APF **(+) ->** Attractive price **(+) ->** Widespread product availability |
| 9 | Widespread product availability **(+) ->** Attractive price **(+) ->** Willingness to consume APF product **(+) ->** Widespread product availability |

*Note.* APF – alternative protein food; (+)-> a positive edge, (-)-> a negative edge.

**Table S13**

*Feedback Loops Developed in System Mapping Workshops in Spain*

| **No.** | **Feedback loops description** |
| --- | --- |
| 1 | Knowledge of protein origin **(+) ->** Age of the client **(+) ->** Knowledge of protein origin |

*Note.* APF – alternative protein food; (+)-> a positive edge, (-)-> a negative edge.

**Table S14**

*Feedback Loops Developed in System Mapping Workshops in Turkey*

| **No.** | **Feedback loops description** |
| --- | --- |
| 1 | Marketing and consumer perception **(-) -->** Cultural and social factors **(+) ->** Preference of APF **(+) ->** Sustainable production **(-) ->** Price of APF **(-) -->** Acceptance of APF **(+) ->** Animal welfare and ethics **(+) ->** Marketing and consumer perception |
| 2 | Marketing and consumer perception **(-) -->** Cultural and social factors **(+) ->** Preference of APF **(-) -->** Price of APF **(-) -->** Acceptance of APF **(+) ->** Animal welfare and ethics **(+) ->** Marketing and consumer perception |
| 3 | Marketing and consumer perception **(-) -->** Cultural and social factors **(+) ->** Preference of APF **(+) ->** Animal welfare and ethics **(+) ->** Marketing and consumer perception |
| 4 | Marketing and consumer perception **(+) ->** Acceptance of APF **(+) ->** Animal welfare and ethics **(+) ->** Marketing and consumer perception |

*Note.* APF – alternative protein food; (+)-> a positive edge, (-)-> a negative edge.
