# Supplementary material for: Mapping determinants of alternative protein food intake across 13 European countries: food system stakeholders’ perspectives
Source: Int J Behav Nutr Phys Act. 2026 Feb 19;23:28. doi: 10.1186/s12966-026-01891-3 (PMC13032592; doi:10.1186/s12966-026-01891-3)

**Mapping Determinants of Alternative Protein Food Intake Across 13 European Countries: Food System Stakeholders’ Perspectives**

**Additional file 1
System Mapping Workshops Manual**

**Table of contents**

1. System mapping workshops manual…………………………………………………... 2

2. Materials for stakeholders (sent 2 weeks before the workshop) ……………………… 21

Determinants of Choosing Alternative Proteins by Consumers

July, 2023

**Acknowledgements:** This manual was developed by Ewa Kulis and Zofia Szczuka based on a COCREATE Systems Thinking & Project Design Meeting in London (March 2019) led by Steve Allender, Andrew Brown, and Josh Hayward from the Global Obesity Centre at Deakin University.

The scripts presented in this manual were documented and developed with the support of the research project “From niche to mainstream – alternative proteins for everybody and everywhere”, funded by the European Union under Grant Agreement No 101083961.

**Understanding Determinants of Choosing Alternative Proteins by Consumers**

- 1. **Roles of Facilitation Team (Richardson & Andersen, 1995)**

The workshop will be conducted by **two persons in each country**: **a facilitator** and **a modeller**.

***The facilitator:*** plays a crucial role in leading the workshop. This includes responsibilities such as starting and ending the meeting, developing system maps, introducing concepts from system dynamics, asking key questions and translating participants’ statements into phrases that are easier for the modeller to use. Additionally, the facilitator is responsible for translation of materials provided by SWPS University for the stakeholders and maintaining communication with them before and after the session.

**General Facilitation Tips:**

- **Manage power dynamics**. If someone is dominating the conversation, you can address it by saying: “Can someone who hasn't spoken yet share their thoughts?” This helps ensure equal participation and creates space for others to contribute.
- **“Parking lot”** refers to a technique used during discussions or meetings where any off-topic ideas, questions, or concerns raised by participants are noted down for future discussion. It allows the facilitator to acknowledge the input without derailing the current agenda and ensures that important points are not forgotten.
- **Prioritize participants' points of view**. The role of the facilitator is to ask questions, encourage reflection, and create an inclusive environment where everyone feels heard and valued.
- **If someone says something truly offensive, address it.** As a facilitator, you are responsible for maintaining a respectful and safe space for all participants.
- **Consider social identities** when facilitating discussions. Recognize that participants may have different backgrounds, experiences, and perspectives based on factors such as race, gender, age, or socioeconomic status. Creating an inclusive environment involves being aware of these identities and ensuring that all participants feel comfortable expressing their views without fear of judgment.

***The modeller:*** operates the STICKE software (drawing the constructs and connections between the constructs), shares the STICKE screen, develops the model, and helps the group reflect on the model structures that emerge during the session, documents important information shared by participants (including the information that has been overlooked by the facilitator). Additionally, **the modeller** provides support to the facilitator by assisting with the translation of materials provided by SWPS University for the participants.

- 1. **Rules of engagement**

**Be Respectful:** All views must be treated with respect, and efforts should be made to promote mutual understanding amongst the participants. Given the multi-stakeholder and intergenerational nature of the Workshop, participants will be reminded that one opinion does not take precedence over another, regardless of age, sex, socioeconomic status, level of education, or other relevant factors.

**Be open and transparent**: The workshop is a space to share, listen, and learn. To promote a participatory and productive dialogue, all participants should have an equal opportunity to voice their opinions and views. Stakeholders should also be transparent about their interests and motivations for participating in the Workshop (including any conflicts of interest).

**Be sensitive to risk and assure safe expression of opinions:** In certain situations, expressing views may involve risks. Moderators and participating stakeholders have a responsibility to take every precaution necessary to minimize the risk of patronizing or exploiting others or of any other negative consequence of their participation.

**Promote empowerment for all:** All participants should feel that their participation was meaningful and that they could influence the structure, process, and outcomes of the dialogue.

- 1. **Research questions for the workshop**

Preparation for the workshop (the first and second questions are also the key questions asked to the participating stakeholders):

**1)**   What factors influence consumers' choices regarding alternative protein-based foods? [According to the stakeholders’ opinion.]

**2)**    How are these factors interrelated?

**3)**    Using a complex system approach to alternative protein choices: Do these factors form feedback loops that drive a change in a system (e.g., move alternative proteins from a niche to the mainstream) or do they maintain the status quo of the system (no change)?

**4)**    What are the similarities and differences between countries and stakeholder groups?

**Evidence base list of „factors”**

- Beliefs, knowledge, and actions of the food system actors (policy makers, lobbyists, consumers, e.g., “moving towards more sustainable food consumption is important to me,” “I worry about financial losses related to changes in the production systems”).

- Key food policies (e.g., financial instruments, education, labelling, advertising, public procurement, food composition, etc.).

- Infrastructure characteristics (e.g., referring to food production, processing, retail systems characteristics?).

- Economic factors (e.g., including national economy, costs of living/disposable income in families, economic impact on food producers and distribution chains).

- Major events that can affect consumers’ choices, such as pandemics, wars, technological developments (AI), political elections creating „windows of opportunity” for policy changes.

- Technological factors, particularly the presence or absence of technological innovations or adoption, and information technology development.

- Environmental and cultural factors (e.g., climate change, sustainability trends, local and organic food trends, food ethics, e.g., animal welfare).

**Note!** Some of these factors may be mentioned by the stakeholders, while others may not. They may also provide a completely different set of factors. It is up to the stakeholders in the workshop.

- 1. **Before the workshop - timeline**

Preparation for the workshop:

*By September 2023*

- The facilitator and modeller translate the informed consents, the handouts for the stakeholders (in both long and short versions), and the post-workshop survey (approx. 20 questions).
- The facilitator and modeler adapt the presentation shared by the team from Poland (titled LIKE-A-PRO_System_Mapping) for their workshops at the local level.

*September 2023*

- The facilitator and modeller set the workshop date and time.
- The facilitator and modeller learn how to use the STICKE software and thoroughly comprehend each step of the workshop. It is also recommended to conduct a trial workshop to test all the procedures and ensure smooth execution in practice.
- The facilitator sends the invitations to the stakeholders.
- The facilitator sends the information materials (with brief information about what the workshop is about, informed consent, etc.).
- The facilitator/modeller purchases workshop promotion materials with LIKE-A-PRO logo/ thank you gifts for the participants.
- The facilitator sends the first batch of the workshop promotional materials to the stakeholders who agreed to take part in the workshop (to keep them engaged).

*1 week before the workshop (October –November 2023)*

- The facilitator sends a reminder to the participants about the workshop via e-mail, including the following information:
- Date and time.
- A link to the online meeting platform. Google Meet is recommended but not required.
- Information that every stakeholder must have access to a large screen (computer, laptop) because it is necessary for viewing the model and map of factors that will be created during the workshops and will consist of multiple boxes, texts, and arrows. This cannot be done on a phone or tablet.
- Request that participants ensure they have a stable internet connection.
- The facilitator also sends the long version of the handouts about alternative proteins to the stakeholders via email.
- The facilitator and modeller verify the proper functioning of the STICKE software and rehearse each step of the workshop once again.
- The modeller prepares a draft email to be sent to the stakeholders, containing the handouts (the same which were sent one week before the workshop), in order to have it ready to be sent at the beginning of the workshop.

*During the workshop*

- The modeller should have a list of email addresses of the participants.
- It is recommended that the facilitator and the modeller be in the same room when conducting the workshop (to consult each other during the workshop and create the final version of the system map).
- The modeller shares the STICKE screen.
- The modeller is responsible for saving the final version of the system map in a .pdf, using this format: 3 first letters of the country, the date of the workshop in the format: dd_mm_year_, and a title: LIKE-A-PRO, e.g., POL_01-09-2023_LIKE-A-PRO

Two people from the SWPS support team will be available before, during, and after the local workshops to assist the local facilitator/modeller in case any issues arise.

Ideally, the workshop should be recorded to facilitate note-taking for each factor included in the map. For example, if the map includes “advertising policies,” it is important to document what stakeholders meant and how they defined it. Please note that participants should be informed in advance and given the option to provide consent to being recorded.

- 1. **Introduction to Group Model Building Session**

| **Steps** | **1. The** **facilitator** welcomes the participants, thanks them for attending, introduces themselves and the modeller, presents the agenda and time break times, explains the purpose of the session, and asks if all participants are ready to begin.  **The modeller:** Shares the presentation screen (Slide 1, Slide 2, and Slide 3).  ***For example:*** “Good morning, everyone! Welcome to today's LIKE-A-PRO workshop on alternative protein choices. Thank you all for attending and taking the time to join us. My name is [Facilitator's Name], and I will be leading this session with [Modeller's Name]. This workshop is a part of the LIKE-A-PRO project, which aims to facilitate sustainable and healthy diets by shifting promising alternative proteins and products from niche to mainstream – making them more available, accessible, and acceptable to all population groups. Alternative proteins are those obtained from sources other than conventional animal-based sources. In particular, LIKE-A-PRO is concerned about proteins from alternative sources such as: RAPESEED KERNEL, MEALWORM, KRILL, MICROBIAL, CULTIVATED MUSHROOM, FERMENTED FUNGAL PROTEIN, PEA.  Before we begin, let’s quickly review the agenda for today. We have a packed schedule ahead of us, and I want to ensure that we make the most of our time. We will be covering the following key points:  - *Exploring the key determinants of alternative protein-based food choices by consumers:* We will delve into the factors that influence consumers when it comes to selecting alternative protein-based food options.  - *Finding connections between different factors:* We will identify and analyze the relationships between the various factors that impact alternative protein-based food choices by consumers.  - *Reviewing the model:* Toward the end of the session, we will review the model we have created, taking a closer look at its components and examining how each factor fits into the larger picture.  The workshop will last about [duration of the workshop]. Throughout our time together, we have scheduled a break at [time of the break]. So, the purpose of today’s workshop is to collaboratively create a comprehensive map of the factors that influence alternative protein-based foods choices by consumers. By the end, we aim to have a clearer understanding of these factors and their interconnections. Before we begin, I want to make sure everyone is ready. If you have any questions or need any clarifications, please feel free to ask.”  **The tips for the facilitator:**   - make sure everyone is present, - make sure that everyone is visible, - if needed, makes sure everyone knows how to use the platform (e.g., how to turn on/ off a microphone, camera, raise a hand).   2. **The facilitator** begins the ice-breaker game. Each participant introduces themselves by stating their name and surname, as well as the stakeholder group they represent (e.g., producer, retail, policy maker, consumer, or advertiser). Next, each participant responds to the question “Never have I ever…” (e.g., “Never have I ever been singing in a choir/ eaten 3-D printed food”). If there is a person who has done it (for example, has sung in a choir), this person is the next to introduce themselves. When no one shows up, the just-introduced person points to the next participant. **The modeller:** Shares screen with the presentation (Slide 4).  ***For example:*** “As we begin our workshop, I believe it’s important for us to get to know each other. To facilitate this, we’re going to start with an ice-breaker game. The objective is for each participant to answer the question ‘Never have I ever...’, introduce themselves, explain which stakeholder group they represent (such as producer, retailer, policy maker, consumer, advertiser), and make their own ‘Never have I ever...’ statement.  Here’s how it works: I will begin by making a statement starting with ‘Never have I ever.’ For example, ‘Never have I ever sung in a choir.’ If any of you have sung in a choir before, please raise your hand or use the ‘raise your hand’ feature on the online platform and introduce yourself. Share your name and state the stakeholder group you represent. Then, take your turn to make your own ‘Never have I ever’ statement to continue the game.  The game will continue until everyone has had a chance to introduce themselves. Feel free to be creative and have fun with your statements.  Now, are the rules clear? If you have any questions, please let me know. Otherwise, let’s get started. My name is XX XX (Facilitator’s name and surname) and I represent (name of the organization represented by the facilitator). Never have I ever eaten a 3-d printed food (…).”  **The tips for the facilitator:**   - make sure that everyone is visible, - make sure everyone has introduced themselves. |
| --- | --- |

- 1. **Key determinants**

| **Steps** | 1. **The** **modeller** sends an e-mail to the stakeholders at the beginning of the session, containing the handouts. The draft of this email has been prepared by the modeller in advance. 2. **The** **facilitator** asks the stakeholders to familiarize themselves with the provided handouts, specifically focusing on the first 8 pages, and gives them ten minutes for this task. Stakeholders are asked to keep their cameras on during this task. If anyone finishes before the 10-minute mark, they indicate this in the chat.   ***For example*:** “Now, I would like to give you some time to go through the handouts that have just been sent to your emails by [Modeller's Name]. As you may recall, these handouts were also sent to you before today's workshop. While I hope most of you are already familiar with them, I'd like us to take the next 10 minutes to review them, focusing especially on the first 8 pages. It’s a good opportunity to refresh our memories. Please keep your cameras on during this task. If any of you finishes before the 10-minute mark, please indicate this in the chat.”     1. **The** **facilitator** instructs the stakeholders that they will first work on their own and prompts them to consider the factors that affect consumers’ choices of alternative proteins using a prompting question **‘In your opinion, what factors influence consumers' choices regarding alternative protein-based foods?’** It is important to ask this particular question because it aligns with the workshop's objectives and ensures consistency across all research teams involved in the LIKE-A-PRO research project. Furthermore, having a standardized research question across teams allows for meaningful comparisons and enhances the overall quality of the results. Also, emphasize that stakeholders are asked to identify factors they personally consider most important.   Stakeholders are encouraged to write down at least five factors. They are given fifteen minutes to identify these factors and reflect on their relative importance. Then, they are asked to organize them hierarchically, placing the more important factors higher in the hierarchy and the less important ones lower.  **The modeller** displays the slide with the task discussed by the facilitator [Slide 5].  ***For example*:** “Now, we will take a few moments to explore the various factors that influence consumers’ choices when it comes to alternative protein-based foods. I want you to consider both the barriers and facilitators that come into play. So, here's the question for you: In your opinion, what factors influence consumers’ choices regarding alternative protein-based foods? Please take some time to reflect on this question and write down at least five factors that come to mind. The factors should be:   - Clear and specific (For example, instead of using ‘Food Quality’ we should use ‘Perceived Taste of Alternative Proteins’). - Concise (For example, instead of using ‘The perceived cost-effectiveness ratio of alternative proteins compared to traditional meat products,’ we should use ‘Price of Alternative Proteins’). - Should not include adjectives that imply increasing/decreasing (For example, instead of using ‘Higher Availability of Alternative Proteins’ we should use ‘Availability of Alternative Proteins’). - When possible, default to the positive/neutral version of the variable (For example, choose ‘Support for Alternative Protein Research’ rather than ‘Lack of Support’).   You have fifteen minutes to complete this task. Once you’ve identified what you believe to be the key factors influencing alternative protein choices, please organize them in a hierarchical order, with the more important factors placed higher in the hierarchy and the less important ones placed lower. When the 15 minutes are up, we will come back together and engage in a round of sharing to discuss the factors that you have identified and I will inform you about the last 3 minutes of the task.  If you finish before the 15-minute mark, please indicate this in the chat. The task is displayed on the screen.”  **The tips for the facilitator:**   - Inform participants about the last 3 minutes remaining for the exercise. Also, provide an update when there is only one minute left and ask a question: “Does anyone need two more minutes?”, ensuring everyone has sufficient time to complete the exercise.      1. **The facilitator** leads a round of sharing, where stakeholders take turns sharing their factors.   **The modeller:** During the sharing session, the **modeller** is adding the variables suggested into STICKE on the “connection circle”. What’s more, the modeller shares the screen displaying factors added in the circle in the STICKE. The modeller is also responsible for documenting clear definitions for the factors mentioned by the stakeholders. This ensures that the meaning of the listed factors is captured accurately and without any ambiguity.  **The tips for the facilitator and the modeller:**   - The facilitator points to the first person and then reaches out person by person to be sure that all stakeholders presented the factors. - The facilitator asks the stakeholders for the first factor (on the top of the list) and then takes a second round of sharing the factors. - The modeller adjusts font size to make the factors visible on the shared screen. - The facilitator and modeller try to remember all the definitions of the factors mentioned by the stakeholders to capture the meaning of the factors. - The modeller is prepared to write down all the definitions of the factors. The definitions are not displayed for the stakeholders but immediately noted by the modeller.   ***For example*: “**Thank you all for taking the time to identify and organize the factors influencing consumers’ choices of alternative protein-based food. Now, let’s move on to the next step. Each of you will have the opportunity to share the factors you have identified. Please start by sharing the factors that are at the top of your list, the ones you consider to be the most important. Share your factor by typing it in the chat, and provide a brief explanation or description for each factor as you share it. Remember, we are interested in both the barriers and facilitators that influence consumers’ choices. Let’s begin with [stakeholder’s name]. Please share the first factor from your list.”  5. **The modeller** puts the factors into the circle (using STICKE) and shares the screen displaying the factors in the circle in the STICKE. The modeller, with the help of the facilitator, needs to ensure that the factors in the connection circle are clear, specific and concise. They should not include adjectives that imply increasing/decreasing; when possible, they should default to the positive/neutral version of the variable. This will help to create meaningful connections between variables in the next exercise.  Figure 1  *Example of the Connection Circle from STICKE Software*    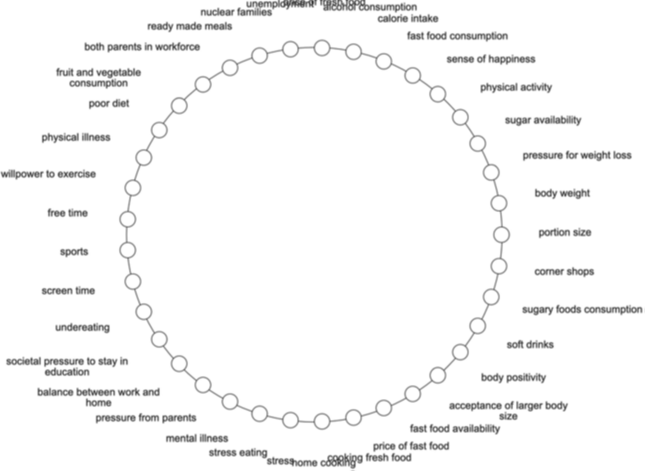 |
| --- | --- |

- 1. **Connection Circles and Diagram View in STICKE**

| **Steps** | 1. **The facilitator** introduces the connection circle script (linking the factors).   ***For example:*** “The next task is to link your factors from the ‘connection circle’ using the STICKE program. The main goal of the connection circle exercise is to find the interrelations between factors that affect consumer choices of alternative protein-based food in your country. In particular, to understand how these factors affect each other.”  **Figure 2**  *Example of the Connection Circle*  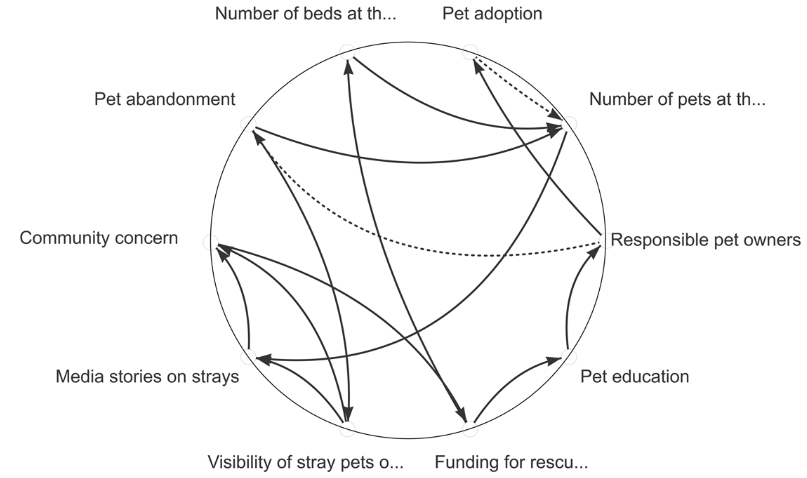  *Note.* *Retrieved from Howard et al. (2020)*     1. **The facilitator** explains the directions of the arrows from the STICKE software and how they show the interrelations between factors. At the same time, **the modeller** shows on the screen the respective examples of positive and negative connections (with corresponding arrows) from the presentation [Slide 6 and Slide 7].   ***For example:*** “Before we begin, let me briefly explain how we’ll use STICKE to show the connections between the factors you’ll identify. We’ll link the factors with two types of arrows, depending on the direction of their relationships.   We will use two types of arrows:  - a **solid arrow**, showing a change in the same direction; we call it a positive connection. It means that if one factor increases, then the other factor also increases or if one factor decreases then the other factor also decreases. [respectively when discussing directions, the facilitator shows both thumbs up👍👍 or both thumbs down 👎👎]”  **The** **modeller** starts sharing their screen and shows the presentation with an example of a positive association [Slide 6].  **The facilitator** explains: “Let’s use an example to illustrate this. If the factor ‘access to the alternative protein-based products’ increases, then the factor ‘purchase of the alternative protein-based products’ also increases [Facilitator demonstrates both thumbs up👍👍]  Or if the factor ‘access to the alternative protein-based products’ decreases, then the factor ‘purchase of the alternative protein-based products’ also decreases. [Facilitator demonstrates both thumbs down 👎👎].  So, in both of these cases it shows a change in the same direction: increase-increase [both thumbs up👍👍] or decrease-decrease [both thumbs down 👎👎]. And, as you can see in the printscreen on the slide from our presentation, we used solid arrow to represent this positive association in STICKE”.  **The** **modeller** stops displaying the screen to ensure that the facilitator is visible to the stakeholders.  **The facilitator** says: “On the other hand, **a dashed arrow** shows a change in the opposite direction; we call it a negative connection. It means that if one factor increases then the other factor decreases, or if one factor decreases then the other factor increases” (respectively when discussing directions, the facilitator shows one thumb up/down and another one in the opposite direction 👍👎 or 👎👍).”  **The modeller** shares their screen once again and shows the presentation with an example for the negative association [Slide 7].  **The facilitator** explains: “Let’s demonstrate this by using the same example. So, one of you might say that if the factor ‘access to the alternative protein-based products’ increases, then the factor ‘purchase of the alternative protein-based products’ decreases [Facilitator demonstrates one thumb up👍 and one thumb down👎]  Or if the factor ‘access to the alternative protein-based products’ decreases, then the factor ‘purchase of the alternative protein-based products’ increases [Facilitator demonstrates one thumb down👎 and thumb up👍]  So, in both of these cases it shows a change in the opposite direction: increase-decrease [one thumb up👍 and one thumb down👎] or decrease-increase [one thumb down👎 and thumb up👍]. And, as you can see in the printscreen on the slide from our presentation, we used a dashed arrow to represent this negative association in STICKE.  Is everything clear so far? If you have any questions or if anything is unclear about this exercise, feel free to let me know. I'll be happy to provide further explanations.”     1. **The modeller** stops showing the presentation and starts showing the connection circle view from the STICKE.      1. **The facilitator** gives the participants 5 minutes to think about connections between the factors presented in the circle. Then, they request each stakeholder to provide their ideas. During this process, the facilitator remembers to ask about the direction of these connections, and the stakeholders indicate the direction with their thumbs. The facilitator goes through two rounds of exploring the connections between the factors shown in the circle. However, if needed, the facilitator allows another round of the discussion for those willing to add more connections. The facilitator ensures that most of the factors receive connections during this task. If there is any factor that doesn't have any connection, and there are no propositions on how to link it, then it can be left unconnected. **The modeller** draws respective arrows in the STICKE.   ***For example:*** “You will have five minutes to think about the connections between the factors presented in the connection circle.”  After 5 minutes, the facilitator announces:  “5 minutes have passed, let’s start sharing your ideas. [Name of the Stakeholder 1], could you begin by identifying two factors that are interrelated? Please use your thumbs to show the direction of the connections between factors.”  **The tips for the facilitator:**   - Take two rounds for sharing examples of the connections. Point to people one by one [Stakeholder 1, Stakeholder 2 Stakeholder 3, ... etc.) to be sure that everyone has spoken. - *Prompting the next examples:*   - “The next person please”;  - “[Stakeholder’s name] it is your turn now. We’d love to hear more from you”;  - “Next connection, please! What do you say, [Stakeholder’s name]?”;  - “[Stakeholder’s name], curious to know if you’ve spotted any new links between these factors?”;  - “[Stakeholder’s name], do you see any other connections between factors”;  - [Stakeholder’s name], I wonder what your point of view is? Any other connections you’d like to share?”;  - “Could you please add one more connection, [Stakeholder’s name]?”     1. **The facilitator** prompts the next turn of the examples. **The modeller** draws respective arrows in the STICKE.   ***For example:*** “To make sure we capture all the connections, let’s start a new round of your suggestions. The next turn for providing examples goes to [Name of the Stakeholder 1]. Would you like to share one more connection between two factors?”     1. After the second round of examples, **the facilitator** opens the floor to discussion, allowing stakeholders to share any additional connections they find important. If there are one or two factors that still need to be connected, the facilitator draws participants’ attention to those factors and encourages them to make the connections (see “The tips” section for guidance). This ensures that all relevant connections are thoroughly explored and discussed. **The modeller** draws respective arrows.   ***For example:*** “We’ve finished the second round of the connections. If anyone wants to share or has discovered another interrelation between factors, please feel free to do so now.”    **The tips for the facilitator:**   - If the stakeholder doesn’t find a new connection during the next round of discussion, the Facilitator switches to the next person: e.g., “[Stakeholder’s name] do you see any more connections? How about you [Stakeholder’s name]? [Stakeholder’s name] it is your turn.” - Helpful phrases for the facilitator during connection circle exercise:   - paraphrasing/clarifying: “I understand that you have in mind the situation when (...)”  - asking for the mechanism: “How does it work?”; “How are these factors directed?” “How do these factors affect each other?”  - summarizing: “You proposed to link xx with zz “; “The suggestion is to link xx with zz”;  - asking for the lacking connections: “Which factor might have a connection with factor AA [a factor lacking any connections]?” What about factor XX?”.  - encouraging the discussion: “Thank you, that’s an interesting point.”; “It is valuable connection”; “It’s a great example”; “I see your point of view”; “That's a good addition to the connections we have so far.””     1. Once most of the factors around the connection circle have at least one connection, **the modeller** switches to diagram view, and the picture is rearranged in STICKE. The facilitator explains what is displayed in this view.   ***For example:*** “We will now switch from the connection circle to a system map view, making it easier to read and understand how interrelated factors affect consumers’ choices of alternative protein-based food. As you can see, our connection circle has now transformed into a map of the factors. To make it easier to read, we need to rearrange the view of the factors. Let’s take a 15 min break for map cleaning. See you at xx [note the time when the break ends].”     1. **The modeller** may need to do a bit of additional cleanup to clarify the diagram. It means that the modeller may change the font, arrange the factors, shift factors for better view, put unrelated factors in one place on a map, and make it easier to read the diagram in order to show feedback loops and casual chains for the next exercise. **The facilitator** becomes familiar with the map.      1. 15-minute break: Participants take a 15-minute break to refresh themselves while **the facilitator and modeller** work to quickly clarify the map. **The modeller** downloads it to the .pdf version. **The facilitator and the modeller** should find at least one example of feedback loop and one example of causal chain:    - **casual chains** such as xx→ yy → zz [showing sequence of the links between factors: how one factor affects the other followed by the next factor].  - **feedback loops** such as  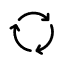 [showing that factors form a circle of connections].  The facilitator explains that feedback loops are important because they reveal the interconnectedness and complexity of systems, helping to predict behaviors, and identify intervention points.   1. After the break: **The facilitator** reminds participants that the map created before the break has been revised by the modeller. **The modeller** shows the screen with the map created by stakeholders in the STICKE.   ***For example:*** “During the break, the connection circle has been rearranged to present the factors and links as a map of factors. Now, you can see the map that illustrates how the factors you mentioned affect consumers’ choices of alternative protein-based foods. Please note that the modeller has not discarded any of your ideas, but rather has visually cleaned up the map, and made some minor improvements to ensure clarity and readability.”     1. **The** **facilitator** asks participants to identify additional connections they can see in this view. In particular, the **facilitator** emphasizes identifying causal chains and feedback loops, encouraging participants to connect several variables into chains or loops rather than focusing only on single connections. The facilitator reminds participants about directions of the associations that dashed or continuous arrows represent. This clarification is important to understand the nature of the relationships between the factors in the system map:   ***For example:*** “Your map shows that the factors xx, yy, zz form:  - casual chains such as xx→ yy → zz [explaining one example of a chain of the factors showing how one factor affects the other and so on]. It means that factors are related in sequence, one by one, and  - feedback loops such as  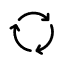 [explaining one example of a loop of the factors showing how factors form a circle of connections]. It means that factors form a circle of connections. Feedback loops are important because they show how different parts of a system connect and affect each other, helping us to predict behaviors and identify intervention points.”     1. **The modeller** sends a .pdf version of the map to all of the participants. The facilitator informs the participants about this.   ***For example:*** “The modeller has just sent you an email with the map. Please open the message and then we will move on to the next task” |
| --- | --- |

- 1. **Model Review**

| **Steps** | 1. **The facilitator** invites participants to work individually. Stakeholders are encouraged to review the map which was sent via email. The map review is planned for 10 minutes. During this task, participants have the opportunity to identify and add any new causal linkages they may find, as well as provide any comments or suggestions about the map. Participants should focus on meaningful links, especially causal chains and feedback loops, instead of just randomly connecting factors. Participants may also add any missing factors, but only if they truly believe them to be essential. **The modeller** shows the screen with the STICKE map.   ***For example:*** “Now, I would like to ask you to take a moment to look at the shared map and work individually. Please focus on how the factors included in the map affect the consumers’ choices of the alternative protein-based foods. You will have 10 minutes to think and refer to what you find good/interesting in the map, as well as any areas that you believe need improvements. If you want to add any more connections, please focus on causal chains and feedback loops, rather than adding connections anywhere. We believe we’ve included the most important determinants on the map, but if you see a crucial factor missing, please bring it up. Later, each of you will have the opportunity to share and discuss your suggestions with a group. Do you have any questions at this moment?”  **The tips for the facilitator:**   - Inform participants about the last three minutes left for the exercise. Also, provide an update when there is only one minute left and ask a question: “Does anyone need two more minutes?”, ensuring everyone has sufficient time to complete the exercise.      1. After the 10 minutes have passed, **the facilitator** encourages participants to share their ideas/changes/observations about the map. The facilitator invites any feedback from the stakeholders, fostering an open discussion and exchange of perspectives. **The modeller** shows the screen with the map in the STICKE. **The modeller** is also responsible for adding new connections between factors identified by the stakeholders.   ***For example:*** “The last task is a round-robin type discussion. You will now have a chance to share some of your observations and discuss them with a group. All changes accepted by the group will be incorporated by the modeller into the shared map. Please start by sharing your discussion points and letting others know what you want to add or remove from the map. Let’s start with the first volunteer.”  **The tip for the facilitator:**   - If nobody is willing to be active, the facilitator points to the first person and then asks each participant. - The facilitator can use the following techniques:   - summarize: “You proposed to link xx with zz “; “The suggestion is to change xx to zz”; “You want to remove yy”  - paraphrasing/clarifying: “"If I understand correctly, you mean the situation when (...)”  - ask for the mechanism: "How does it work?”; “How are these factors directed?” “Can you explain how these factors affect each other?     1. As **the facilitator** is eliciting new information from the group and guiding the discussion, **the modeler** captures the changes in STICKE, which is being projected on the screen in real time. After each proposition from each stakeholder, the facilitator asks the group about the proposed changes. **The facilitator** must ensure that everyone agrees with the changes incorporated into the map.   ***For example:*** “What do others think about proposed changes? Do others agree/disagree with new material/removal/ alteration? Does anyone want to suggest any further revisions to the map?”   (If the modeller doesn’t understand the discussed changes, the modeller asks the facilitator for clarification or repetition of the arrangements.)   1. With five minutes to go, **the facilitator** alerts the group about the approaching end of the workshop and gives an opportunity for a final discussion. **The modeller** presents the final map.   ***For example:*** “We are almost out of time, and we can make two or three quick last-minute changes before we end the workshop. It is time for any final feedback about the map.”  **The tips for the facilitator:**   - If a person speaks for an extended period, the facilitator uses “the parking lot” method to manage the discussion. For instance, they may say, “Thank you for sharing your thoughts. Please pause for the moment and see what others have to say about this” or “It is a lot of information, could you provide a short summary” or “Thank you for sharing but we need to stop here for now, you can back to this point later [giving opportunity for other stakeholders to share their thoughts]”      1. Individual questionnaire: **The modeller** sends a link to a questionnaire to all participants which includes both sociodemographic questions and questions about stakeholders’ beliefs regarding factors related to consumers' choices of alternative proteins. **The facilitator** informs the participants that the final element of the workshop is filling out a survey and emphasizes its importance.   ***For example:*** “Before we end our workshop, I would like to ask you to fill out the short questionnaire. This questionnaire is a very important complement to the workshop. It concerns potential barriers and factors facilitating a shift in the food system towards alternative protein choices. You should have received an email with ​​the survey. Please take five minutes to complete it before we close the meeting. Thank you!”  **The questionnaire includes the following questions (to be sent as google forms online questionnaire):**  - 1:       Your country (please select from the list of the countries)  - 2.              Sector and the type of organization (e.g., farming industry, ingredients industry, food processor company, marketing, education, retail); (please select from the list)  - 3.        Job title (e.g., sales manager, regional manager, innovations specialist, etc.) (please select from the list)  - 4.       Years of work in a similar position/similar organization) (please select from the list)  - 5.       Gender (please select from the list)   - 6. Age (please select from the list: 18–25; 25–35, 35–45, 45–55, 55–65, >65)  -  7.      In your opinion:  - *does the map lack any important factors*? If so, please write them down-  Rate the importance of the groups of factors determining an increase of alternative protein choices in your country:   (not important at all) -3 -2-1; 0 (neutral) +1+2 +3 (extremely important)  Beliefs, knowledge, actions of policy makers -3-2-1 0 +1+2+3  Beliefs, knowledge, actions of producers -3-2-1 0 +1+2+3  Beliefs, knowledge, actions of retail representatives -3-2-1 0 +1+2+3  Beliefs, knowledge, actions of consumers -3-2-1 0 +1+2+3  Food composition policies (e.g., nutrients and sources) -3-2-1 0 +1+2+3  Food labelling policies -3-2-1 0 +1+2+3  Advertising policies -3-2-1 0 +1+2+3  Food marketing policies (other than advertising) -3-2-1 0 +1+2+3  Public procurement provision policies (food at schools, hospitals, public administration institutions, etc.) -3-2-1 0 +1+2+3  Food retail policies (regulating what is available in retail outlets) -3-2-1 0 +1+2+3  Taxation policies -3-2-1 0 +1+2+3  Other fiscal or food prices policies (e.g., subsidies) -3-2-1 0 +1+2+3  Education policies -3-2-1 0 +1+2+3  Trade and investment agreements -3-2-1 0 +1+2+3  Infrastructure in food production -3-2-1 0 +1+2+3  infrastructure in food processing -3-2-1 0 +1+2+3  Infrastructure in retail -3-2-1 0 +1+2+3  Economic situation of the country (e.g., GDP, national budgets, etc.) -3-2-1 0 +1+2+3  Cost of living in the country -3-2-1 0 +1+2+3  Producer/retail costs of introducing novel food -3-2-1 0 +1+2+3  Disposable income/ economic situation of families -3-2-1 0 +1+2+3  Major events that can affect consumers’ choices (e.g., COVID-19 pandemics, wars in European continent) -3-2-1 0 +1+2+3  Political elections in my country -3-2-1 0 +1+2+3  Technology innovation needed for the alternative protein production -3-2-1 0 +1+2+3  Technological innovations and developments in marketing (e.g., Artificial Intelligence) -3-2-1 0 +1+2+3  Climate change -3-2-1 0 +1+2+3  Trends toward more sustainable choices -3-2-1 0 +1+2+3   Local and organic food trends, food ethics (e.g., animal welfare) -3-2-1 0 +1+2+3  Note: The questionnaire items are based on  - INFORMA food environment monitoring modules <https://www.informas.org/modules/>  - INFORMA Food Environment Policy index <https://www.jpi-pen.eu/images/reports/Food-EPI_EU_FINAL_20210305.pdf>  - CICI framework for Context and Implementation of Complex Interventions (<https://implementationscience.biomedcentral.com/articles/10.1186/s13012-017-0552-5>)   1. **The facilitator** closes the workshop.   ***For example:*** “Thank you for attending the LIKE-A-PRO workshop. To sum up, we now have the final map of the factors. The map shows how these factors are interrelated and form a system that affects consumer choices of alternative protein-based food. It visually presents the causal loops and chains of the factors that will help the researchers understand the system in which they operate. We are very pleased with the work you’ve done during the workshop. Please feel free to ask if you have any questions. Otherwise, it is time to say goodbye. Thank you once again for your valuable contributions!” |
| --- | --- |

- 1. **After the workshop**

Tasks for the **facilitator** and the **modeller**:

- Based on the discussion during the workshop, the facilitator and the modeller write down the list of factors with their definitions mentioned by stakeholders (immediately after the workshop).
- The modeller **saves the final map created during the workshop in .png and .csv versions** (immediately after the workshop).
- **The facilitator or the modeller must send the final map to the LIKE-A-PRO Polish Team (**[**zszczuka@swps.edu.pl**](mailto:zszczuka@swps.edu.pl) **or** [**ekulis@swps.edu.pl**](mailto:ekulis@swps.edu.pl)**) in the .png and .csv versions (immediately after the workshop)**. The map should be named based on the instructions on page 8 of this manual, e.g., POL_01-09-2023_LIKE-A-PRO.

**References**

Richardson, G. P., & Andersen, D. F. (1995). Teamwork in group model building. *System Dynamics Review*, *11*(2), 113–137. <https://doi.org/10.1002/sdr.4260110203>

Savona, N., Macauley, T., Aguiar, A., Banik, A., Boberska, M., Brock, J., Brown, A., Hayward, J., Holbæk, H., Rito, A. I., Mendes, S., Vaaheim, F., van Houten, M., Veltkamp, G., Allender, S., Rutter, H., & Knai, C. (2021). Identifying the views of adolescents in five European countries on the drivers of obesity using group model building. *European journal of public health*, *31*(2), 391–396. <https://doi.org/10.1093/eurpub/ckaa251>

**MATERIALS FOR STAKEHOLDERS: WORKSHOPS ON FOOD SYSTEM MAPPING**

**What is LIKE-A-PRO?**

The LIKE-A-PRO project aims to facilitate sustainable and healthy diets by shifting promising alternative proteins and products from niche to mainstream - making them more available, accessible, and acceptable to all population groups. This includes young people, adults, elderly, vulnerable groups, such as people of low socio-economic status, ethnic minorities, and those living in rural locations.

**LIKE-A-PRO has set out to….**

- Understand what determines consumer behaviour and the necessary food ecosystem governance frameworks that enable a higher uptake of alternative protein products
- Diversify the alternative protein supply & developing new alternative protein products, increasing the availability of these products in the European market while ensuring consumer acceptability.
- Empower food system actors to make alternative protein products an easy and economically viable choice via their diversified & increased market supply and ensured favourable food ecosystem conditions.
- Ensure that the project developments in alternative protein products and FEs will bring positive changes in terms of health and sustainability of the European food system, while remaining in line with regulatory frameworks and ethical requirements

**LIKE-A-PRO unites 42 dedicated partners across 17 countries**


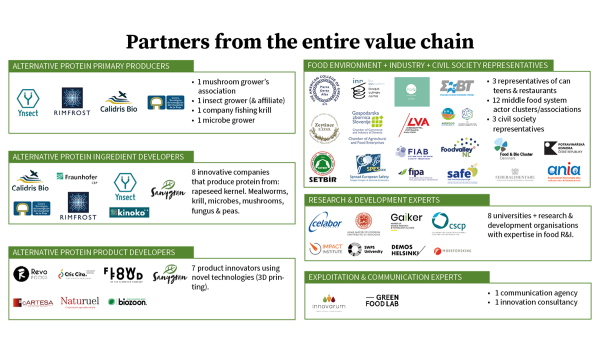


**visit us on** <https://www.like-a-pro.eu/> **for more information**

**Which alternative proteins will be discussed?**


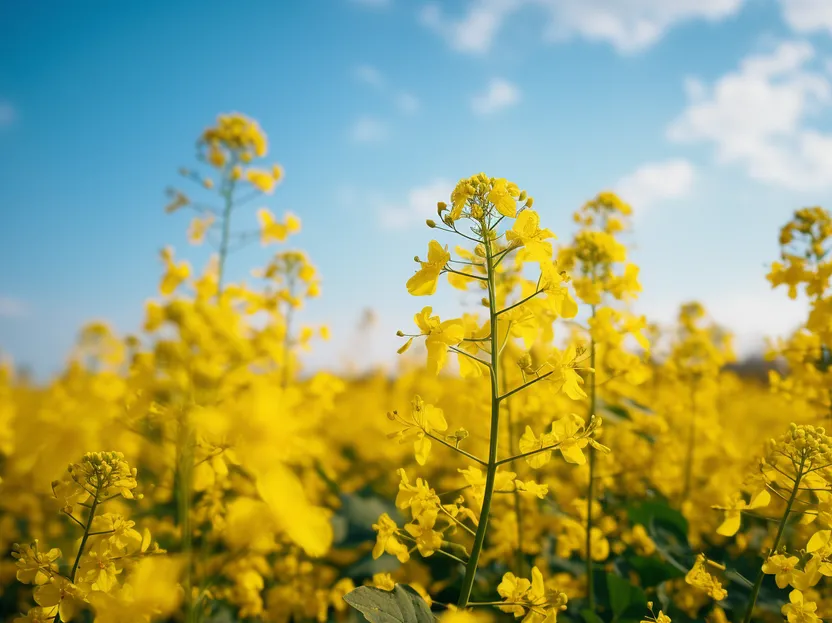


**RAPESEED KERNEL PROTEIN** (by FRAUNHOFER)

- protein concentrate obtained from rapeseed after oil extraction in a process that guarantees high protein content (55%) & quality. The LIKE-A-PRO rapeseed kernel protein has good functional properties (solubility, foaming, emulsifiability) and a very good amino acid profile. Rapeseed is a widely grown EU crop that is GMO-free.

**MEALWORM PROTEIN** (by YNSECT) –
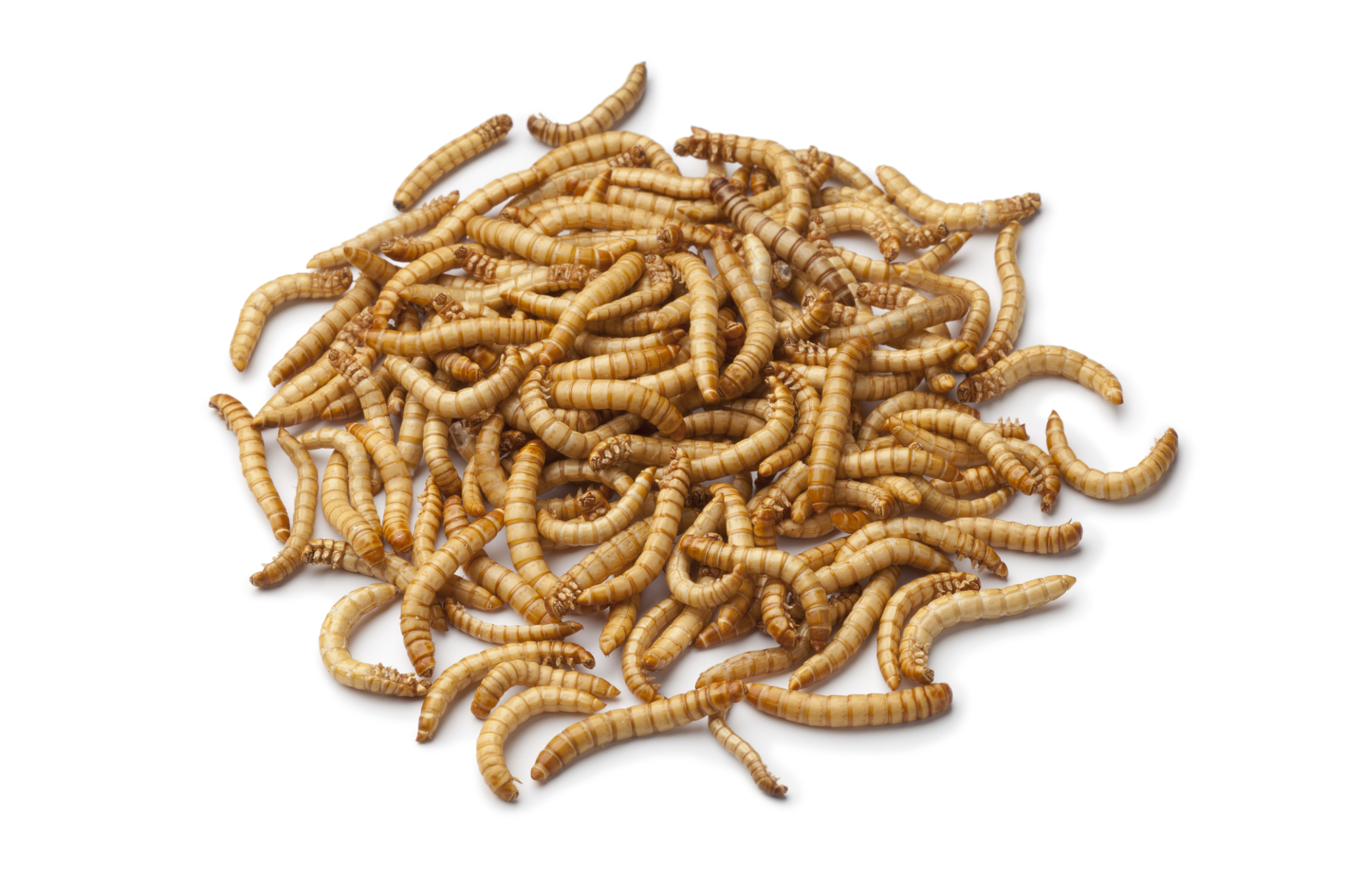


high protein concentrate (70-80%) - dossier sent to EFSA for two protein concentrates - derived from the processing of the larvae of lesser mealworm (Alphitobius diaperinus) and yellow mealworm (Tenebrio molitor). Mealworm proteins are well digestible, contain all 9 essential amino acids, are high in vitamins, minerals & fibre (chitin).

**KRILL PROTEIN** (by MOREFOSKING + RIMFROST) –
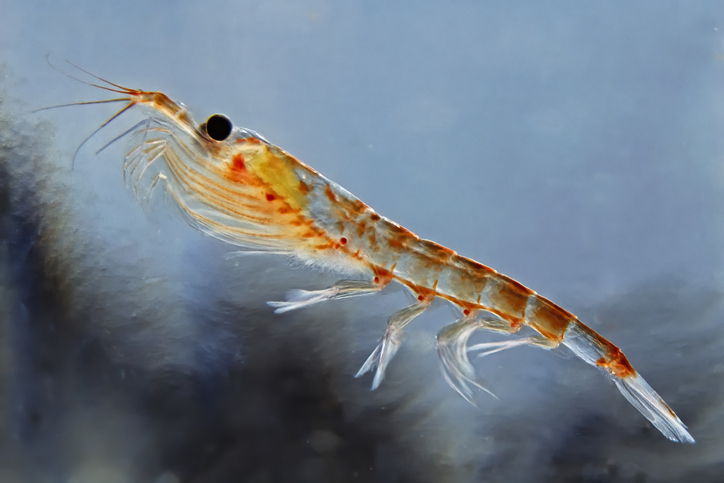


protein concentrate derived fromAtlantic krill (Euphausia superba, small crustaceans rich in high quality protein, while low in fat). Currently, krill fisheries produce oil supplements for the human consumption, while by- products (krill meal/krill shells) are underutilised (applied mainly for aquacultural or petfood). Thus, increasing krill raw material sustainable uses is a clear market need. The LIKE-A-PRO krill protein concentratehas high protein content and contains antioxidant astaxanthin and polyphenols.


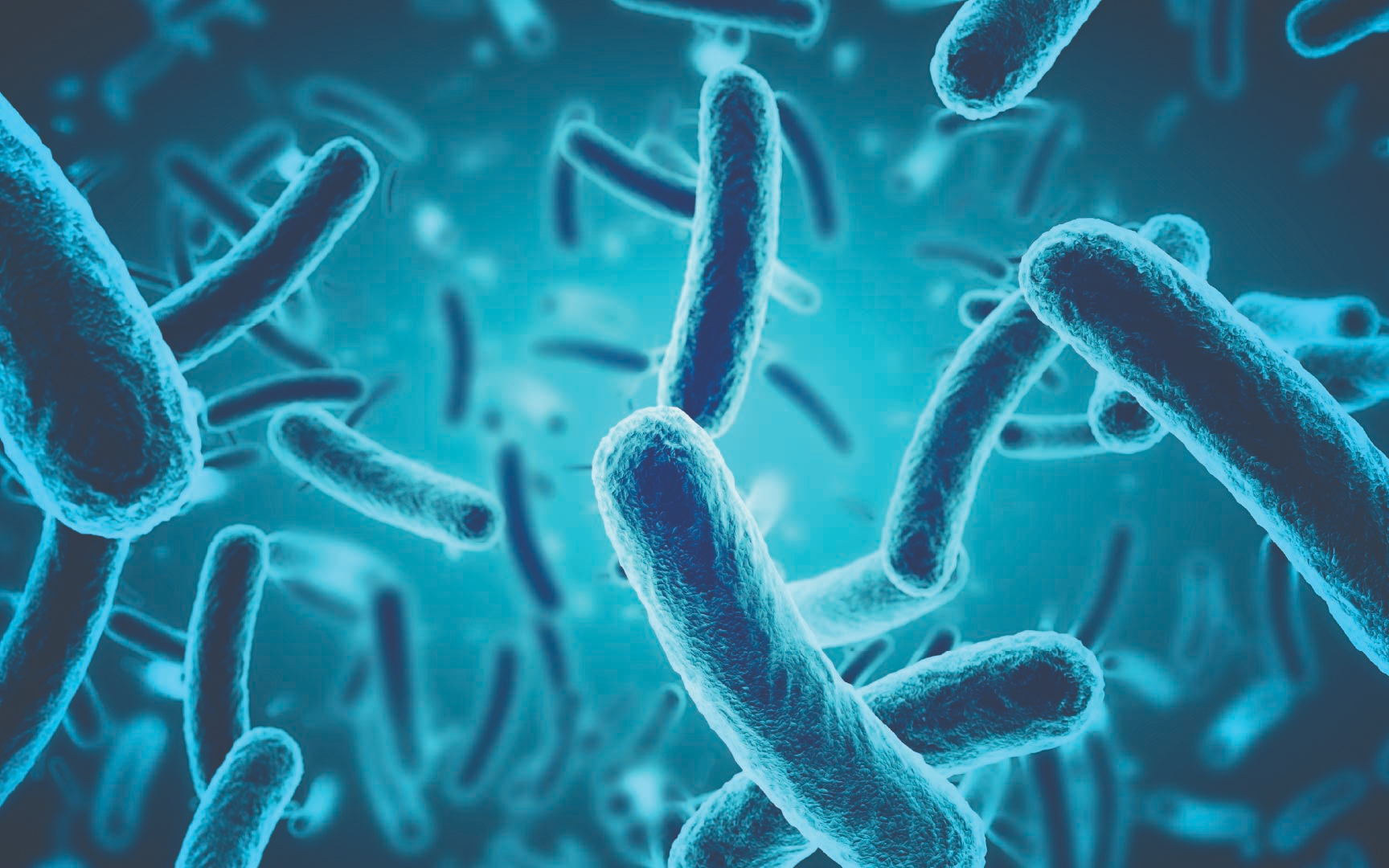


**MICROBIAL PROTEIN** (by CALIDRIS) –

microbial biomass of fast-growing Methylotrophs (proprietary natural and non-GMO strain), produced via fermentation of renewable methanol. The microbial biomass has a high protein content (65-70%), with a high amount of essential amino acids (meeting the human nutrition needs as by FAO/WHO). It has excellent functionality (e.g., emulsion capacity) allowing for clean label products and has constant quality (easy to blend in standard food formulations).
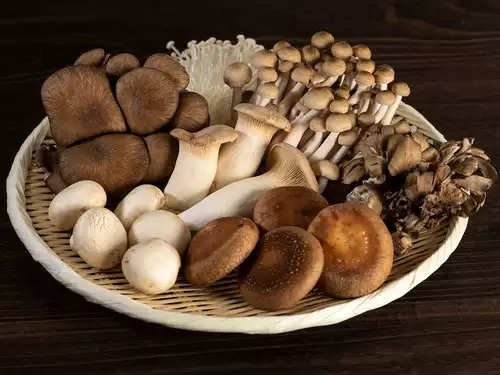


**CULTIVATED MUSHROOM PROTEIN** (by CTICH) –

protein concentrate from discarded cultivated mushrooms such as white button mushroom (Agaricus bisporus), oyster mushroom (Pleurotus ostreatus) or shiitake (Lentinula edodes). Mushroom industry by-products are managed like waste, even though they are a rich source of nutritionally useful essential amino acids.


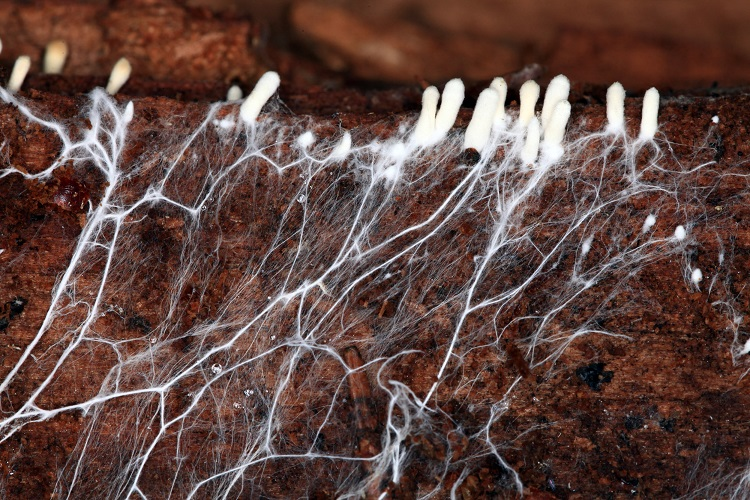


**FERMENTED FUNGAL PROTEIN** (by KINOKO) –

obtained from fermentation of the mycelium of an edible fungus on pulses such as lentils, chickpea, lupin or pea. An heirloom fungal strain is used, packed with important nutrients, minerals, fibre, and protein (contains all 9 essential amino). The fermented fungal protein is of great mouthfeel, texture, and taste.

c
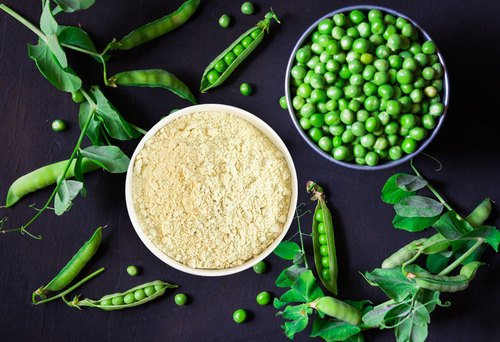


**PEA PROTEIN** (by SANYGRAN) –

protein concentrate obtained from yellow peas (Pisum sativum). It has a good essential amino acid profile and a homogeneous powder form, suitable for different food formulations.

**What is a system map?**

A system map in psychology research is a visual representation of the complex interplay among various components of a system within a specific psychological construct. It can include elements such as individuals, groups, institutions, and environmental factors, and their relationships or interactions. They help to identify key factors, their relationships, and how changes in one component might affect others.

In a system map, components of the system are often depicted as nodes, and their relationships are depicted as lines or arrows. The nature of these relationships can vary widely - they can be causal relationships, correlations, influences, or dependencies, for example.

Source: Castellani, B. (2018). Map Your Theory: An Introduction to System Dynamics Modeling for the Social and Behavioral Sciences. *Springer.*


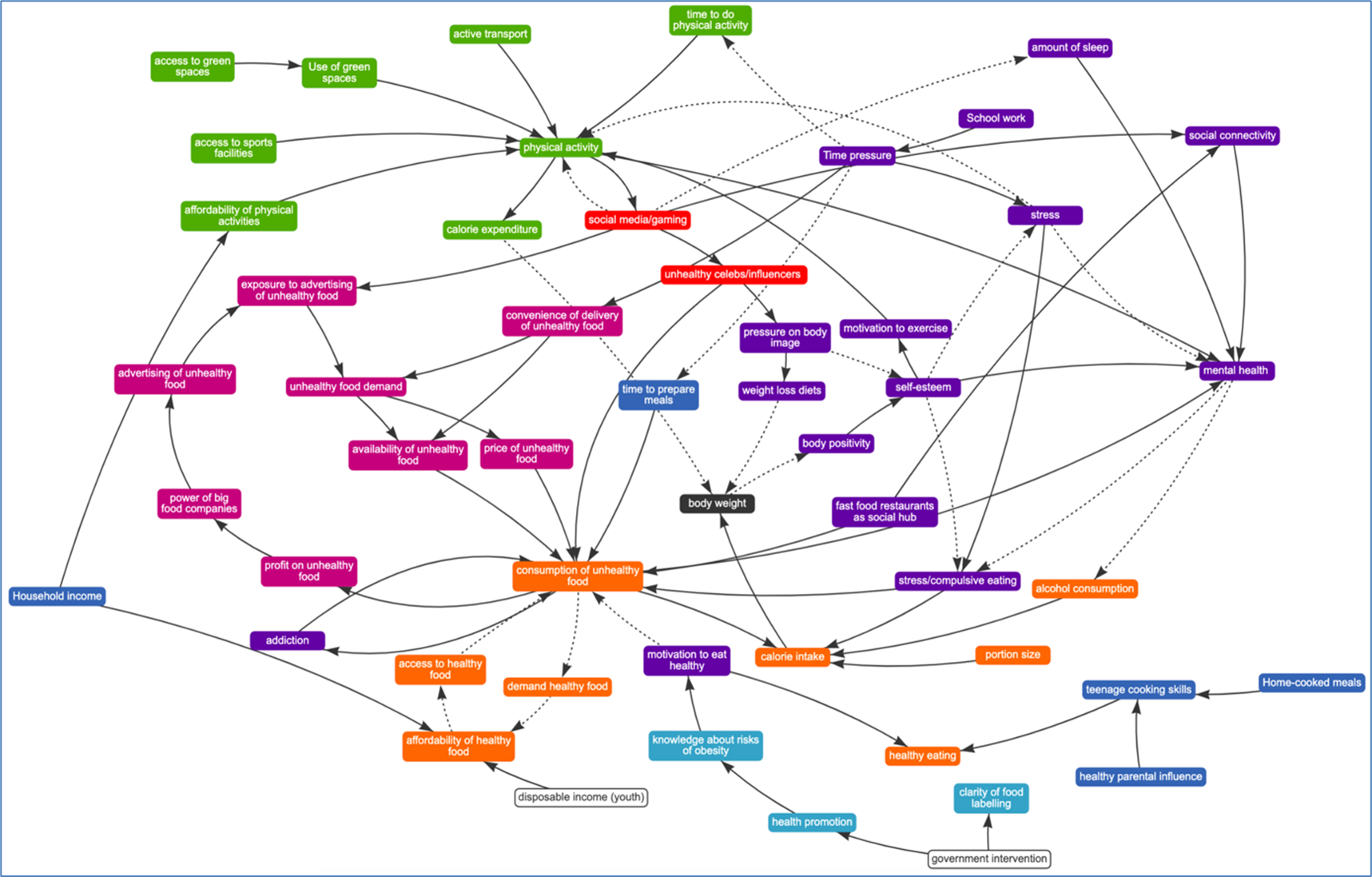


**Figure:** An example of a system map of factors that contribute to adolescent obesity. The map shows not just the factors but also the ways in which they may be causally related to each other and to obesity; solid lines - positive relationship, dotted lines - negative relationship; (taken from: Savona et al., 2021).

**What is a food system?**

Food systems comprise all the people, institutions, places, and activities that play a part in growing, processing, transporting, selling, marketing, and, ultimately, eating food. Food systems influence diets by determining what kinds of foods are produced, which foods are accessible, both physically and economically, and peoples’ food preferences. They are also critical for ensuring food and nutrition security, people’s livelihoods, and environmental sustainability.

As shown in the framework below, the different parts of the food system include food supply chains, food environments, and individual factors. Food systems also encompass crosscutting issues and drivers (factors that push or pull at the system, some being exogenous to food systems). The components, crosscutting issues, and drivers all shape food systems and can lead to both positive and negative outcomes.


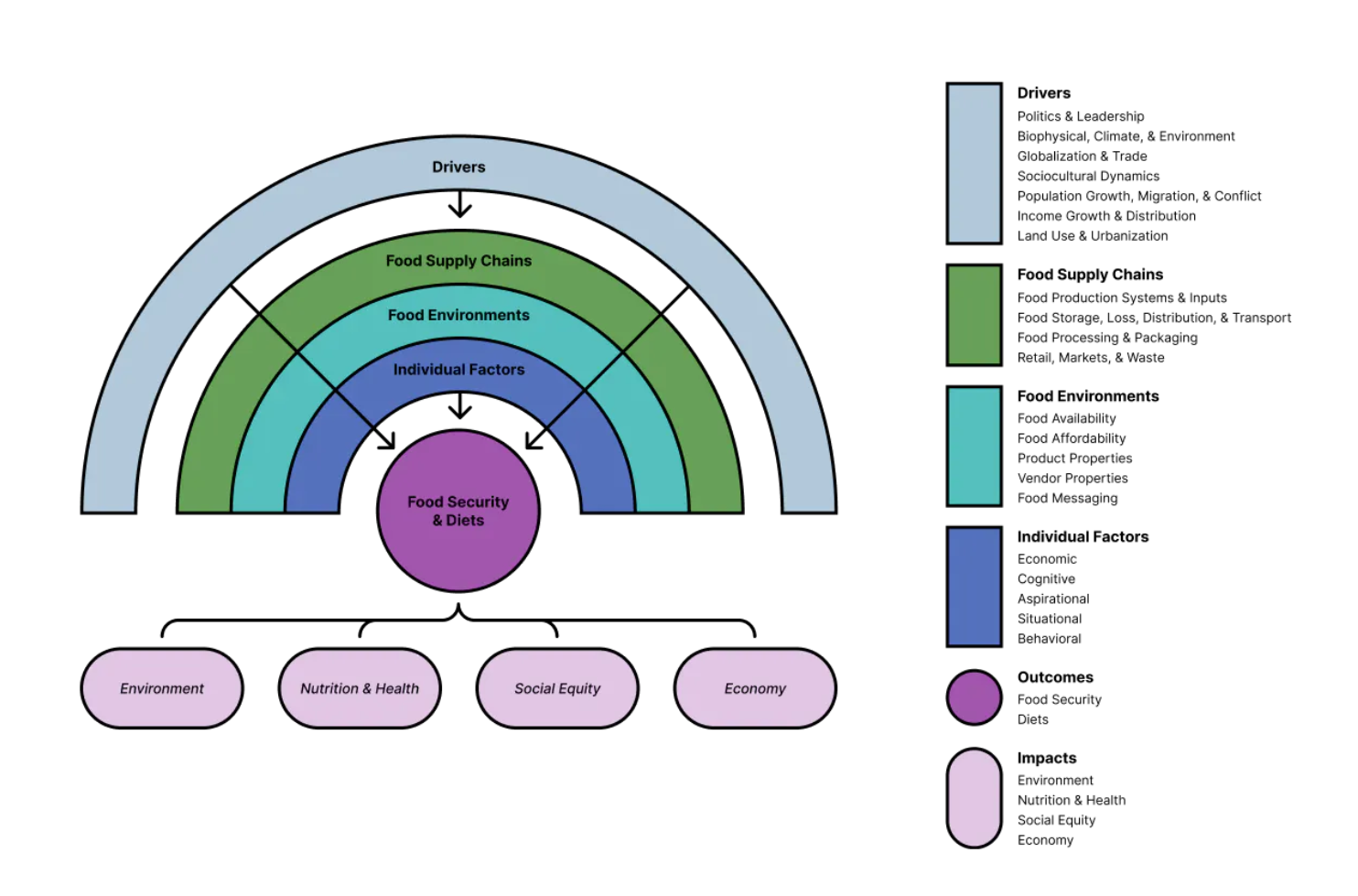


Figure source: foodsystemsdashboard.org/information/about-food-systems

**What types/group of determinants are influencing alternative protein choices?**

The adoption of alternative proteins is shaped by a multifaceted interplay of determinants, encompassing environmental concerns, health motivations, ethical considerations, cultural and social influences, technological advancements, economic factors, and the impact of advocacy and education. As these determinants continue to evolve, the landscape of alternative protein choices will likely undergo further transformations, contributing to a more sustainable and diversified global food system.

**
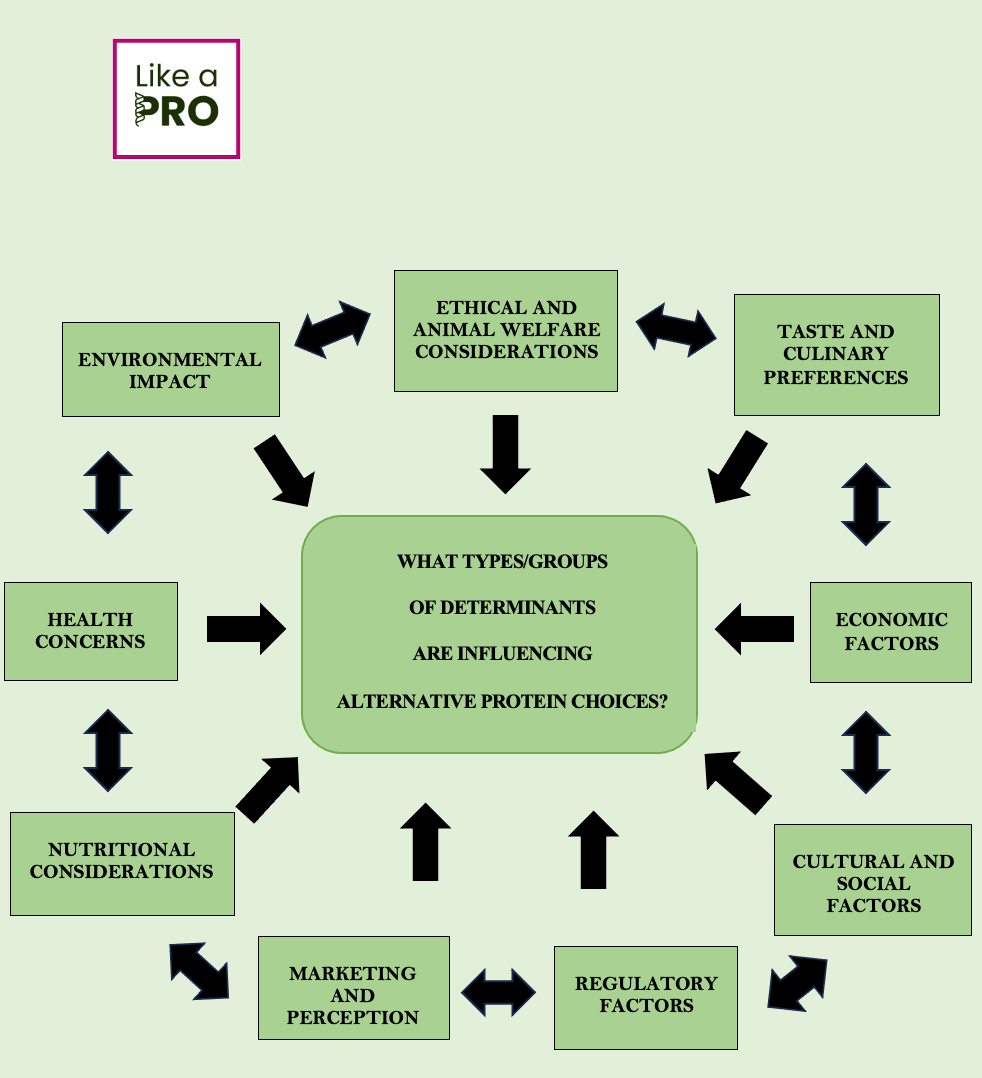
**

Figure based on Bhat et al., 2014; Bouvard et al., 2015; Craig, 2009; Dossey et al., 2016; Gochfeld & Burger, 2005; Savaiano, 2014; Tack et al., 2020, Dewan & Tamang, 2007; Lusk & Norwood, 2011; van Huis, 2013; Zeller & Pauly, 2005

**Moreover, based on the increasing amount of research, the factors that may be specific to individuals (or consumers) include:**


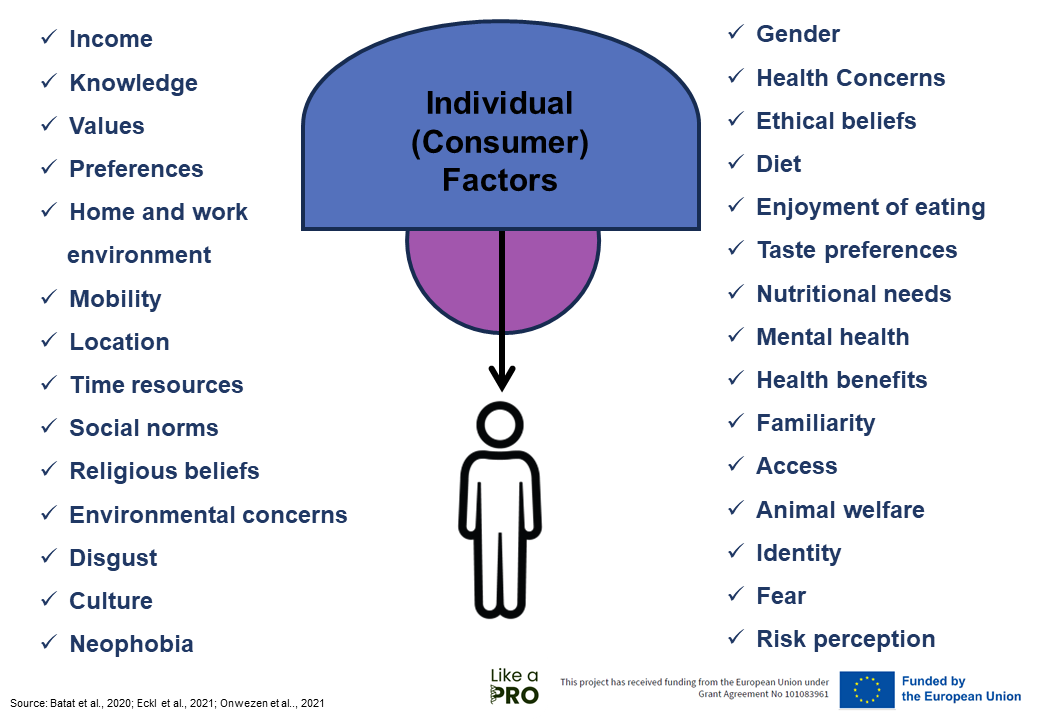

Supplement: Supplementary file 2 — Supplementary Material 2. [file 12966_2026_1891_MOESM2_ESM.docx]
